# Visible-Light-Controlled Histone Deacetylase Inhibitors for Targeted Cancer Therapy

**DOI:** 10.1021/acs.jmedchem.2c01713

**Published:** 2023-01-19

**Authors:** Laia Josa-Culleré, Amadeu Llebaria

**Affiliations:** MCS, Laboratory of Medicinal Chemistry & Synthesis, Department of Biological Chemistry, Institute for Advanced Chemistry of Catalonia (IQAC-CSIC), Jordi Girona 18-26, 08034Barcelona, Spain

## Abstract

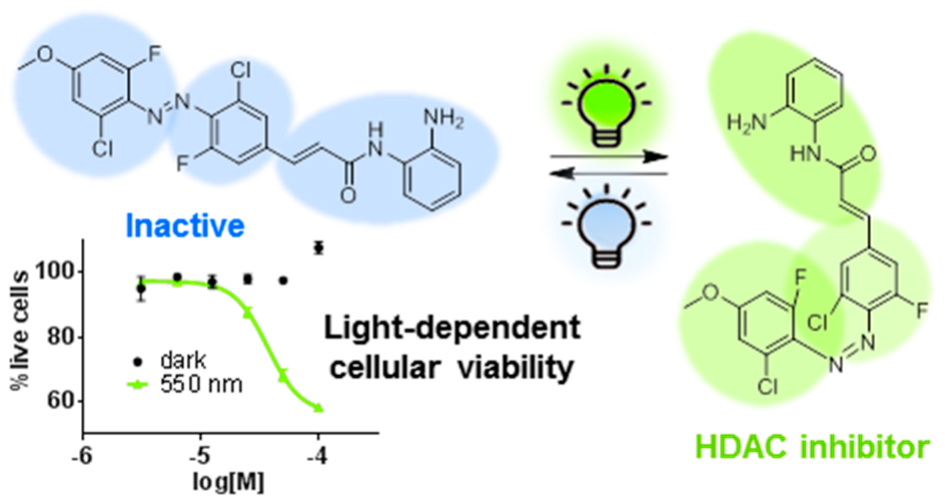

The lack of selectivity of anticancer drugs limits current chemotherapy. Light-activatable drugs, whose activity can be precisely controlled with external light, could provide a more localized action of the drugs in the tumor, thus decreasing side effects and increasing efficacy. Herein, we introduce a series of photoswitchable azobenzene histone deacetylase inhibitors (HDACis) whose activity can be controlled by external visible light. Initial HDACis isomerized under ultraviolet light and were up to >50-fold more active under illumination than in the dark in enzyme assays. These were then optimized toward compounds responding to more permeable and less harmful green light by introducing *o*-halogen atoms into the azobenzene. Selected compounds decreased cell viability only under illumination in four different cancer cell lines. Overall, we present photoswitchable HDACis with optimized activation wavelengths, which inhibit enzyme activity and cell viability only upon illumination with visible light, contributing to the still limited toolbox of photoswitchable anticancer drugs.

## Introduction

A prevailing limitation of anticancer drugs is that they often fail to fully differentiate between cancer and healthy cells. This lack of selectivity limits their therapeutic window, which both decreases treatment’s efficacy and leads to undesired side effects. In parallel to the investigation of new targeted antitumor drugs, it is important to identify novel approaches that enable a more selective and localized effect of such drugs. For this purpose, several drug delivery systems have been developed, which direct the drug to the site of interest using internal (pH,^1^ reactive oxygen species,^2^ or overexpression of enzymes^3^ and receptors^4^) or external (temperature,^5^ ultrasound,^6^ or light^7,8^) triggers. Among these, light is particularly attractive given its noninvasive and remote action, together with a precise modulation by adjusting the wavelength, intensity, and time of exposure.

Photopharmacology uses light as an external trigger to control drug activity with high spatiotemporal precision. It is based on molecules that change their structure upon illumination under particular light conditions, which is used to exercise an external control over its target receptor and finally in their biological function. The design of light-activatable molecules relies on two main approaches: caging and photoswitching. In the caging approach, a bioactive molecule is chemically modified with a photoremovable group that renders it inactive, and the active molecule is released at the target tissue upon illumination.^9^ In the case of photoswitches, a photochromic moiety is introduced within the structure of bioactive molecules; this enables reversible on/off switching between biologically active and inactive forms when illuminated.^10^ While the design of cages is simpler as it can employ well-validated drugs, the reversibility of photoswitches allows for greater control of biological function, as both the initiation and termination of drug effects and its dose can be locally controlled by light. Among the known photochromic moieties, azobenzenes are the most popular.^11−14^ They can isomerize between the more stable *trans* configuration to *cis* under illumination with a particular wavelength, which is dependent on the type and substitution of the aryl groups. This results in a large change in polarity, geometry, and end-to-end distance, which can lead to a difference in target engagement.

Photopharmacology has huge potential in the field of cancer, where a more localized effect of drugs could be achieved via illumination of the tumor area. To date, some examples of azobenzene-based molecules have been described, which show more potent anticancer activities under illumination conditions than in the dark *in vitro*(15−19) and in nematodes.^20^ However, there has been a lack of further progression to more advanced *in vivo* rodent models. On top of the standard medicinal chemistry parameters that need to be considered for the progression of small molecules to *in vivo* studies (potency, physicochemical and pharmacokinetic properties, etc.), for photoswitching molecules, photochemical properties also need to be optimized. These include the wavelength of isomerization, photostationary state (PSS) distribution, photochemical conversion efficiency, and thermal half-life of the metastable isomer.^21^ Arguably, the optimal photochemical profile of anticancer azobenzenes still needs to be determined, and for this purpose, molecular tools with a range of properties would be invaluable. As localized and dynamic control of drug action is pursued, photoisomerization parameters and kinetics must be optimized to correlate with the target receptor activity, timings and biological rythms, and physiologiocal conditions to obtain an improved therapeutic effect.

Histone deacetylases (HDACs) are promising targets in oncology, as they can reverse cancer-associated epigenetic states.^22,23^ These enzymes remove acetyl groups from the amino-terminal lysine residues on histone tails and nonhistone proteins. This leads to condensed chromatin, which limits the binding of transcription factors to promoter sequences and represses gene expression. However, the broad pharmacology and lack of specificity of HDACis limit their clinical use.^24^ Therefore, they are an ideal target for the development of photoswitchable inhibitors.

**Figure 1:**
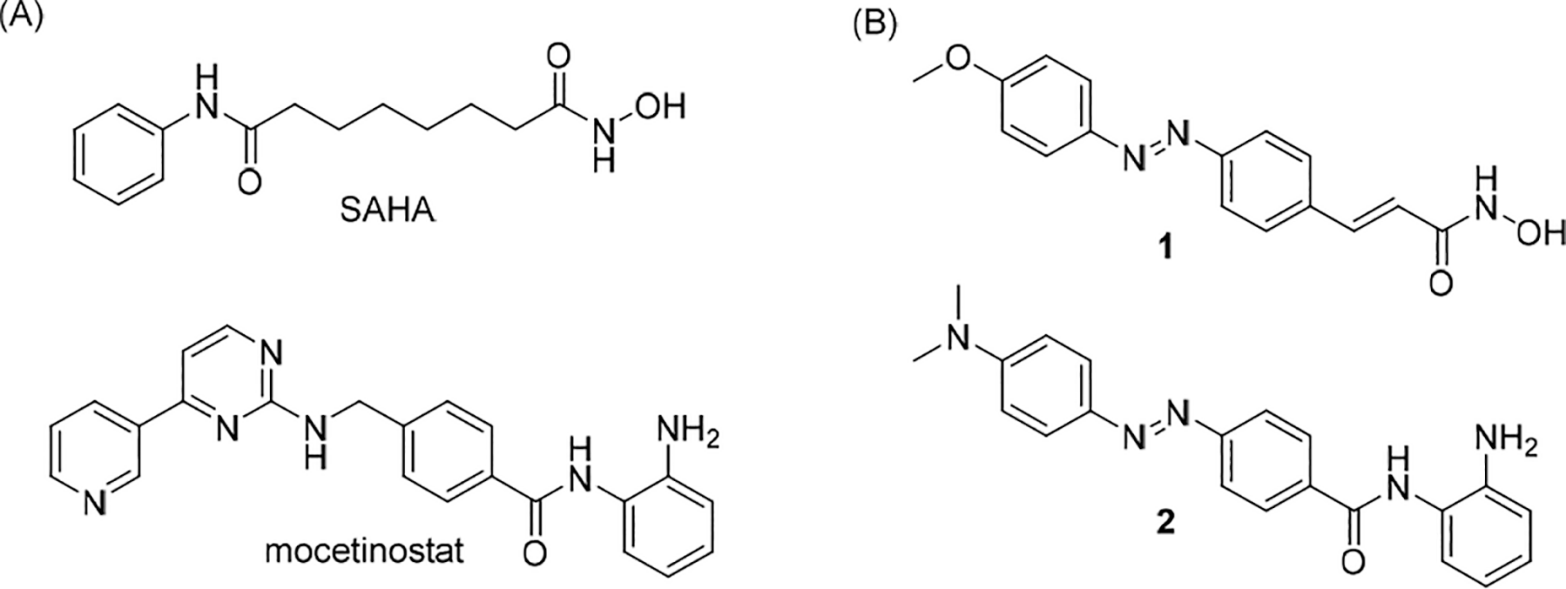
Reported (A) conventional and (B) photoswitchable HDAC inhibitors.

Most classical HDAC isozymes have a zinc-dependent active site, for which many examples of HDAC inhibitors have been described. These are divided into different structural classes, namely, hydroxamates, benzamides, cyclic peptides, and short chain fatty acids.^23,25^ The typical pharmacophore of zinc-dependent HDACis consists of three structural elements: (1) a zinc binding domain (ZBD), which chelates the zinc atom at the active site pocket of the enzyme; (2) a spacer/linker, which is usually hydrophobic and lies in the channel; and (3) a cap group, which sits on the surface of the channel and can be exploited to confer isoform selectivity.^26,27^ The most common zinc chelators are hydroxamic acids (HA) and *o*-aminoanilide (OAA), such as SAHA and mocetinostat, respectively (Figure 1A). HAs are often less selective but more potent than OAAs.^28,29^ The reported photoswitchable HDACis utilize an azobenzene as the photoswitchable moiety, which is incorporated onto the linker region (Figure 1B). The top compounds from these studies are HA **1**(18,30) and OAA **2**,^19,31^ both of which are more active in the *cis* form than in the stable *trans* form. While HA **1** isomerizes under a low wavelength of 365 nm and relaxes at a relatively slow rate (*t*_1/2_ = 4.2 h at room temperature in buffer), OAA **2** has a stronger push–pull character that increases the activation wavelength to 470 nm and decreases its half-life to 60 μs at room temperature in PBS but exerts a surprising long lasting inhibitory activity in the presence of the target HDAC protein.

In this work, we aimed to understand the SAR of the reported azobenzenes to design optimized photoswitchable HDAC inhibitors. First, we identified analogues with larger differences in enzyme activity between isomers. Second, we increased the wavelength of isomerization from the ultraviolet (UV) to the visible region. Third, we obtained compounds with robust differences in activity in cell viability assays.

## Results and Discussion

**Scheme 1:**
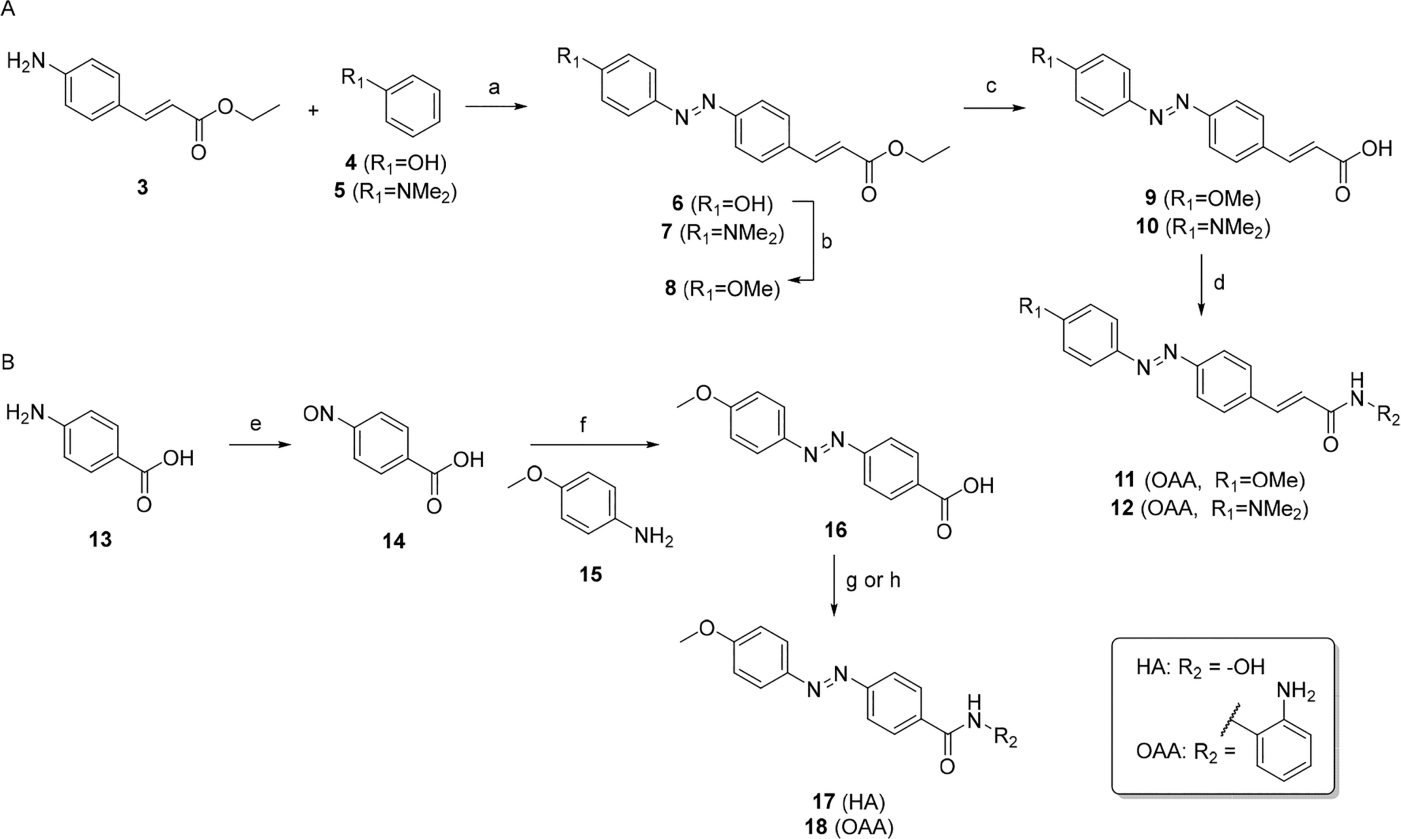
Synthesis of a First Library of Azobenzene HDACis Reagents and conditions: (a) (i) NaNO_2_, HCl, MeOH/H_2_O, −5 °C to rt, 10 min; (ii) **4** or **5**, KOH, −5 °C to rt, 18 h, 44% (quant); (b) MeI, K_2_CO_3_, acetone, 50 °C, 2 h, 85%; (c) NaOH, EtOH, rt, 8–18 h, 20–87%; (d) *o*-phenylenediamine, DIPEA, EDC, HOBt, DMF, rt, 18 h, 31–55%; (e) oxone, DCM/H_2_O, rt, 1 h, 95%; (f) DCM/AcOH, 35 °C, 18 h, 53%; (g) (i) *O*-THP-hydroxylamine, DIPEA, EDC, HOBt, DMF, rt, 18 h, 55%; (ii) HCl, 1,4-dioxane, rt, 30 min, 46%; (h) (i) *tert*-butyl (2-aminophenyl)carbamate, DIPEA, EDC, HOBt, DMF, rt, 18 h, 37%; (ii) TFA, DCM, rt, 2 h, 66%.

The reported photoswitchable HDACis differ in their ZBG, linker, and cap group. We first wanted to understand the influence of these elements on the photochemical properties and biological activity of the analogues, which would guide further optimization. For this purpose, we prepared a library of azobenzene-containing HDACis (Table 1). Analogues containing the alkene linker were accessed from acrylate **3**,^18^ which generated the azobenzene moiety via formation of the diazonium salt (Scheme 1). Further ester hydrolysis and amide coupling gave the desired compounds **11** and **12**. For HAs, we obtained higher and more consistent yields by preparing the *O*-THP-protected hydroxamate before a final deprotection to the final compounds, rather than reacting ester **7**/**8** directly with hydroxylamine. Synthesis of analogues **17** and **18** without the alkene proceeded via Mills reaction. With the small library of photoswitchable HDACis in hand, we proceeded to evaluate their photochemical properties. We determined (1) the optimal wavelength to isomerize the thermostable *trans* to the *cis* configuration, (2) the rate of back-isomerization to the *trans* configuration after illumination, and (3) the proportion of each isomer under different light conditions. These data are summarized in Table 1, and the full data set can be found in Figures S1–S8.

**Table 1:**
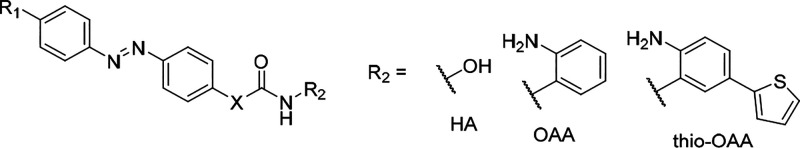

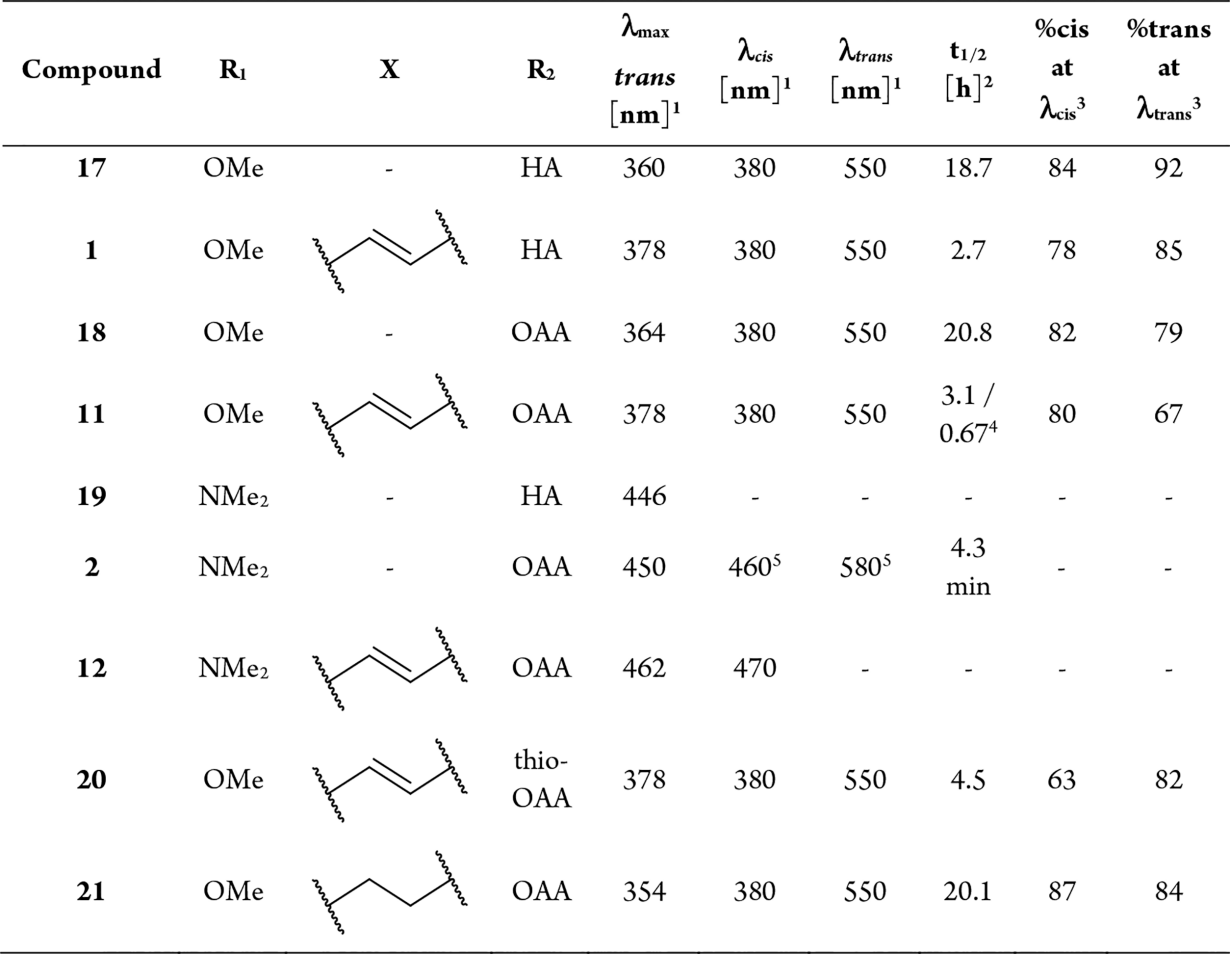
Photochemical Properties of a First Library of Azobenzene HDACis

1 Determined by measuring UV–vis spectra at 25–50 μM in DMSO.

2 Determined by measuring UV–vis spectra at 25–50 μM in DMSO over time at room temperature after illumination with λ_*cis*_.

3 Determined by HPLC at the isosbestic point of each isomeric pair (310–338 nm) after illumination of a 100 μM solution in DMSO with either λ_*cis*_ or λ_*trans*_.

4 In DMEM with 0.1% DMSO at 37 °C, measured by HPLC.

5 Using CoolLed at 217 mW/cm^2^ instead of Teleopto plates.

The λ_max_ of the *trans* isomer, and consequently the optimal wavelength of isomerization, was mainly influenced by the *para* substituent of the azobenzene. The dimethylamine analogues had λ_max_ values of 446–462 nm, while the methoxy analogues had λ_max_ values of 360–378 nm; hence, the former isomerized under blue-light irradiation and the latter under violet-light irradiation. As expected, the dimethylamine analogues have a greater push–pull character and relaxed much quicker than the corresponding methoxy, and in some cases, the relaxation was too fast to observe the absorbance spectrum of the *cis* isomer. For instance, **18** had a *t*_1/2_ of 20.8 h, while its close pair **2** had a *t*_1/2_ of 4.3 min.

The strongest effect of the alkene linker was on the back-isomerization rate, decreasing the half-life 6–7-fold as compared to that of the nonlinker or saturated analogues (**17** vs **1**, and **18** vs **11** and **20**). The alkene also induced a 14–18 nm bathochromic shift.

**Figure 2:**
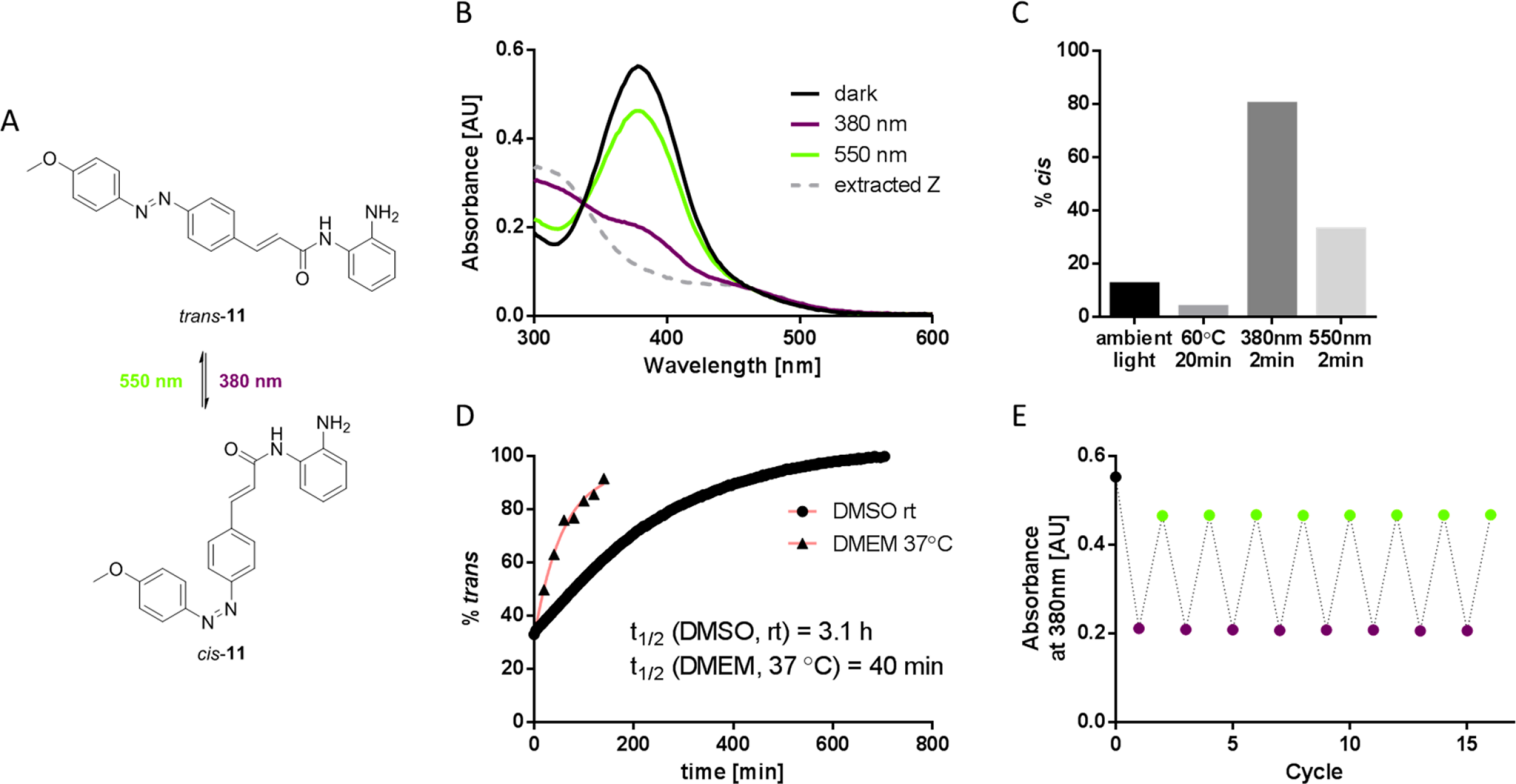
Photochemical properties of OAA **11**. (A) Chemical structures of photoisomers of **11**. (B) UV–vis spectra of **11** (25 μM) in DMSO under different light conditions; the extracted *Z* was calculated from the irradiated spectra (380 nm) and its known *E*/*Z* composition. (C) Quantification of the *E*/*Z* composition of **11** (100 μM) in DMSO after different temperature and light conditions, measured by HPLC at the isosbestic point (321 nm). (D) Half-life estimation of *cis*-**11** after irradiation (380 nm) following its absorbance at 380 nm, in DMSO at room temperature (25 μM) or DMEM at 37 °C (10 μM). (E) Absorbance of **11** (25 μM) in DMSO at 380 nm after multiple illumination cycles (380–550 nm, 2 min each). In all cases, illumination at 380 nm was performed at 11 mW/cm^2^ and that at 550 nm at 13 mW/cm^2^.

Figure 2 shows a summary of the photochemical properties of compound **11**, which later was found to give among the most promising biological performances. The optimal conditions of isomerization of **11** from *trans* to *cis* and vice versa were with near-ultraviolet (380 nm) and green light (550 nm), respectively (Figure 2B). The conversion ratio from *cis* to *trans* was high (80%), and back-isomerization occurred with moderate efficiency, recovering 67% of the *trans* configuration (Figure 2C). Thermal relaxation occurred with a *t*_1/2_ of 3.1 h in DMSO at room temperature, and under conditions for cellular assays (37 °C in DMEM with 0.1% DMSO), it decreased to 40 min (Figure 2D). **11** was stable after multiple *cis*/*trans* isomerization cycles (Figure 2E).

To study the inhibition of HDAC enzymes by the compounds, we used a fluorogenic assay with human recombinant HDAC1.^32^ The enzyme was incubated with an acetylated substrate and each inhibitor for 1 h at room temperature, under either dark or light conditions. We opted for illumination throughout the incubation period to maintain the maximum proportion of *cis* under light conditions. To obtain the maximum proportion of *trans* under the dark conditions, the stock solutions were heated to 60 °C for 20 min before the assay, which was shown to be sufficient to achieve 93.3–99.9% of *trans* (Figures S1–S8). Then, the deacetylated substrate generated a fluorescent coumarin after incubation of the mixture with trypsin at 37 °C for 20 min.

We identified appropriate illumination intensities for each wavelength, which were high enough to reach PSS but not damaging to the enzyme. For this purpose, we first determined the minimum light intensity required to isomerize the compounds (results for compound **11** are shown in Figure S19 as an example). Then, we compared the activity of the nontreated enzyme under dark and illumination conditions and determined the dose–response curves of SAHA under both conditions. In general, the chosen conditions did not affect the enzyme activity (Figure S20). In some isolated replicates, we observed small differences in absolute fluorescence values between light conditions, but in these cases, the IC_50_ values of SAHA remained unchanged (Figure S21).

**Figure 3:**
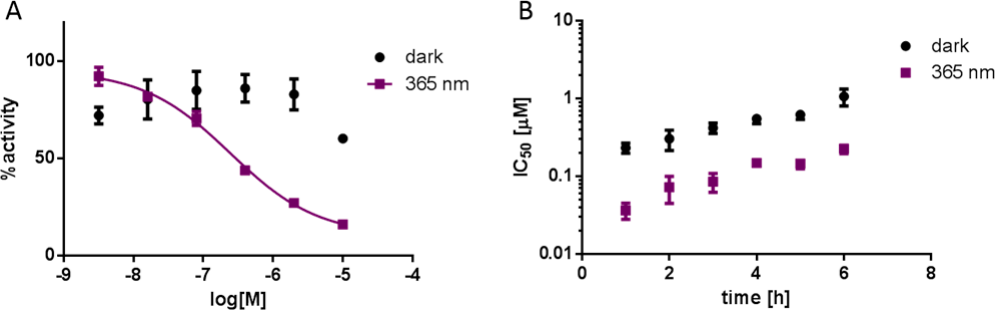
Inhibition of recombinant HDAC1 by the first library of azobenzenes. (A) Dose–response curve of **11** against HDAC1 under either dark or illumination conditions; IC_50_ (dark) > 10 μM, and IC_50_ (365 nm) = 200 nM. (B) IC_50_ values of **1** against HDAC1 following incubation with the enzyme for different times after being either kept in the dark or illuminated for 30 min. Illumination at 365 or 380 nm, 2 mW/cm^2^; stocks kept at 37 °C in the dark overnight before use.

Table 2 shows the activity of the tested compounds under dark and light conditions. The *cis* isomer of HA **1** gave the highest potency with an IC_50_ of 52 nm, consistent with previous reports showing that HAs are more potent than the corresponding OAAs.^28^ However, alkene-containing OAA **11** (Figure 3A) and thiophene-OAA **20** gave the largest difference between both isomers, being 50- and 29-fold more active under light than dark conditions, respectively. Saturated OAA **21** was also active only in the *cis* form, albeit with a potency lower than that of the corresponding alkene **11**. Interestingly, only HA **17** was more active in the *trans* form than in the *cis* form.

**Table 2:** Inhibition of Recombinant HDAC1 by the First Library of Azobenzenes^a^

	IC_50_ (μM)		
compound	dark	light	IC_50_(dark)/IC_50_(light)
**17**	0.42	2.9	0.14
**1**	0.96	0.052	18
**18**	1.2	0.20	6
**11**	>10	0.20	>50
**20**	>10	0.35	>29
**21**	>10	3.4	>2.9

a IC_50_ values under either dark or illumination (365 or 380 nm) conditions, measured by a fluorescence assay.

With an IC_50_ of 200 nM in the *cis* form, OAA **11** was chosen as the most promising photoswitchable HDACi from this small library, and it was used for further optimization.

We also wanted to assess whether enzyme binding would stabilize the more active *cis* form and extend its half-life. For this purpose, the enzyme was incubated with **1** for 6 h, either with or without preillumination of the compound, and enzyme activity was then assessed every hour. We found that the difference in activity between dark and illuminated conditions was maintained over time (Figure 3B). Considering that the *t*_1/2_ of this compound in DMSO and a buffer is 2.7 h, these results suggest that enzyme binding stabilizes the azobenzene in its less stable *cis* form.

To further optimize the compounds for future *in vivo* use, it was necessary to find inhibitors that were activated under visible light. Among the strategies that have been described to increase the isomerization wavelength of azobenzenes, we focused on the introduction of halogen atoms *ortho* to the azo moiety.^33−36^ This modification is known to separate the n → π* absorption bands of the two isomers, which often overlap around 400–500 nm, allowing their use to trigger isomerization under green light.

**Scheme 2:**
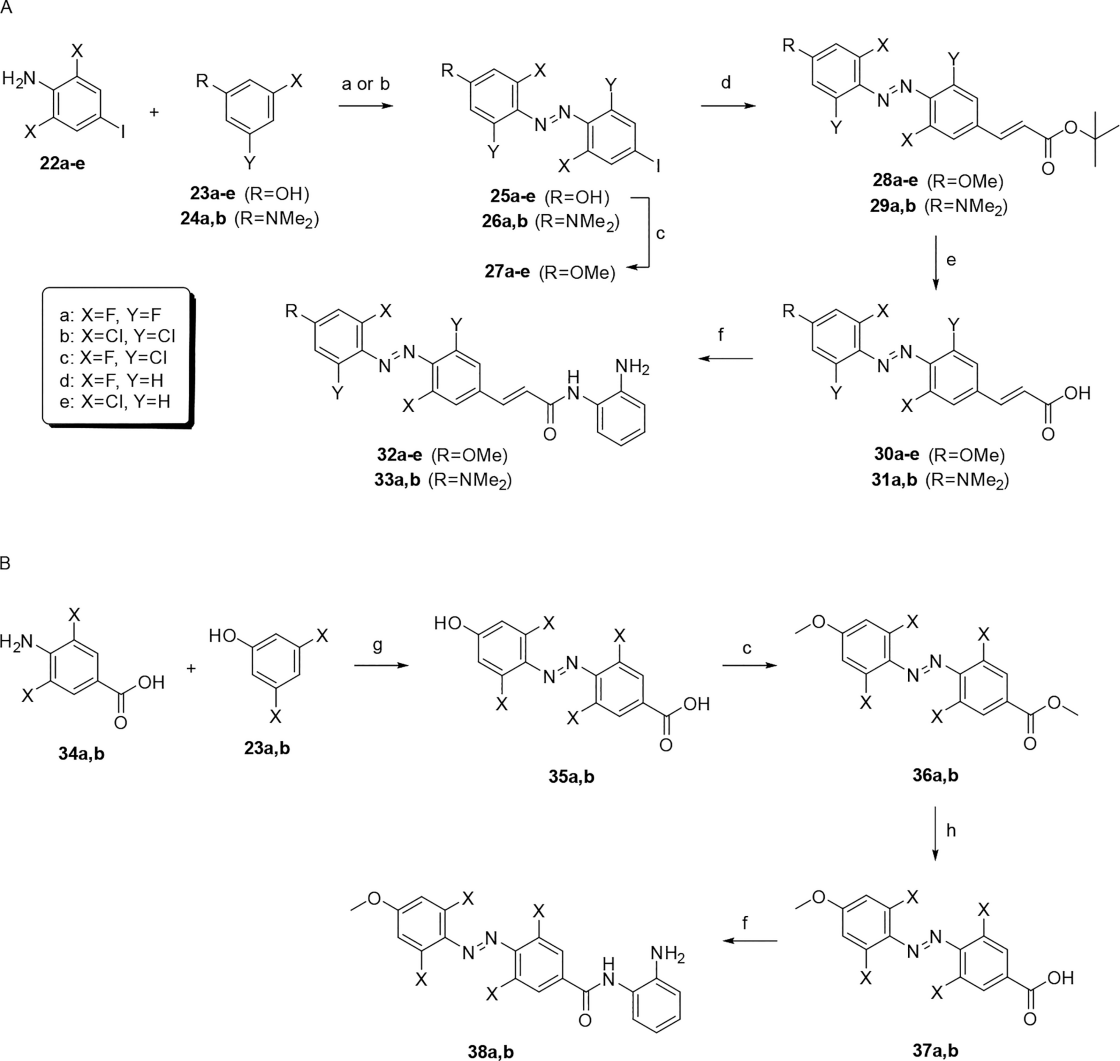
Synthesis of Halogenated Azobenzene HDACis Reagents and conditions: (a) for R = OH, (i) NaNO_2_, HCl, H_2_O, 0 °C, 20 min; (ii) **23a–e**, NaOH, 0 °C, 2 h, 25–95%; (b) for R = NMe_2_, NaNO_2_, H_2_SO_4_, AcOH, DMF, 0 °C, 2 h; (ii) **24a** or **24b**, NaOH, 0 °C to rt, 3 days, 40–74%; (c) MeI, K_2_CO_3_, acetone, 50 °C, 2–3 h, 86–95%; (d) *tert*-butyl acrylate, P(*o*-tol)_3_, Pd(OAc)_2_, Et_3_N, DMF, 100 °C, 18 h, 45–75%; (e) TFA, DCM, rt, 1 h, quant; (f) *o*-phenylenediamine, DIPEA, EDC, HOBt, DMF, rt, 18 h, 28–84%; (g) (i) NaNO_2_, HCl, H_2_O, 0 °C, 1 h; (ii) **23a** or **23b**, NaOH, K_2_CO_3_, 0 °C, 1 h; (h) NaOH, THF, MeOH, H_2_O, rt, 18 h, 40% quant.

We thus introduced di- and tetra-*ortho* fluorine and chlorine atoms to the scaffold of azobenzene **11**, affording **32a–e**. These compounds were prepared via formation of the diazonium salt of premade **22a–e**, reacting with phenol **23a–e**, respectively, to form the azobenzene moiety, followed by phenol methylation (Scheme 2A). The alkene was then introduced through a Heck reaction with *tert*-butyl acrylate, which after deprotection and amide coupling with *o*-phenylenediamine furnished the desired products.

For the sake of comparison, tetrafluoro and tetrachloro substitutions were also introduced into non-alkene OAA **18**. These compounds were also prepared via formation of the diazonium salt to give azobenzenes **35a** and **35b** (Scheme 2B). We thereafter obtained better yields by methylating both the phenol and acid groups, followed by ester hydrolysis and amide coupling to **38a** and **38b**.

It was also of interest to prepare the tetrafluoro and tetrachloro analogues with the *p*-dimethylamine. While push–pull azobenzenes with the *p*-dimethylamine have among the fastest thermal relaxation rates, tetrahalogenated azobenzenes are among the most stable, and both substitution patterns provide bathochromic shifts; thus, it was of interest to combine both features and determine the photochemical properties of the resulting compounds **33a** and **33b**. These were prepared in a manner similar to that of the corresponding *p*-methoxy **32a** and **32b**. In this case, reaction of the diazonium salts with dimethylamine **24a** and **24b** was much slower than with phenols **23a–e**.

**Figure 4:**
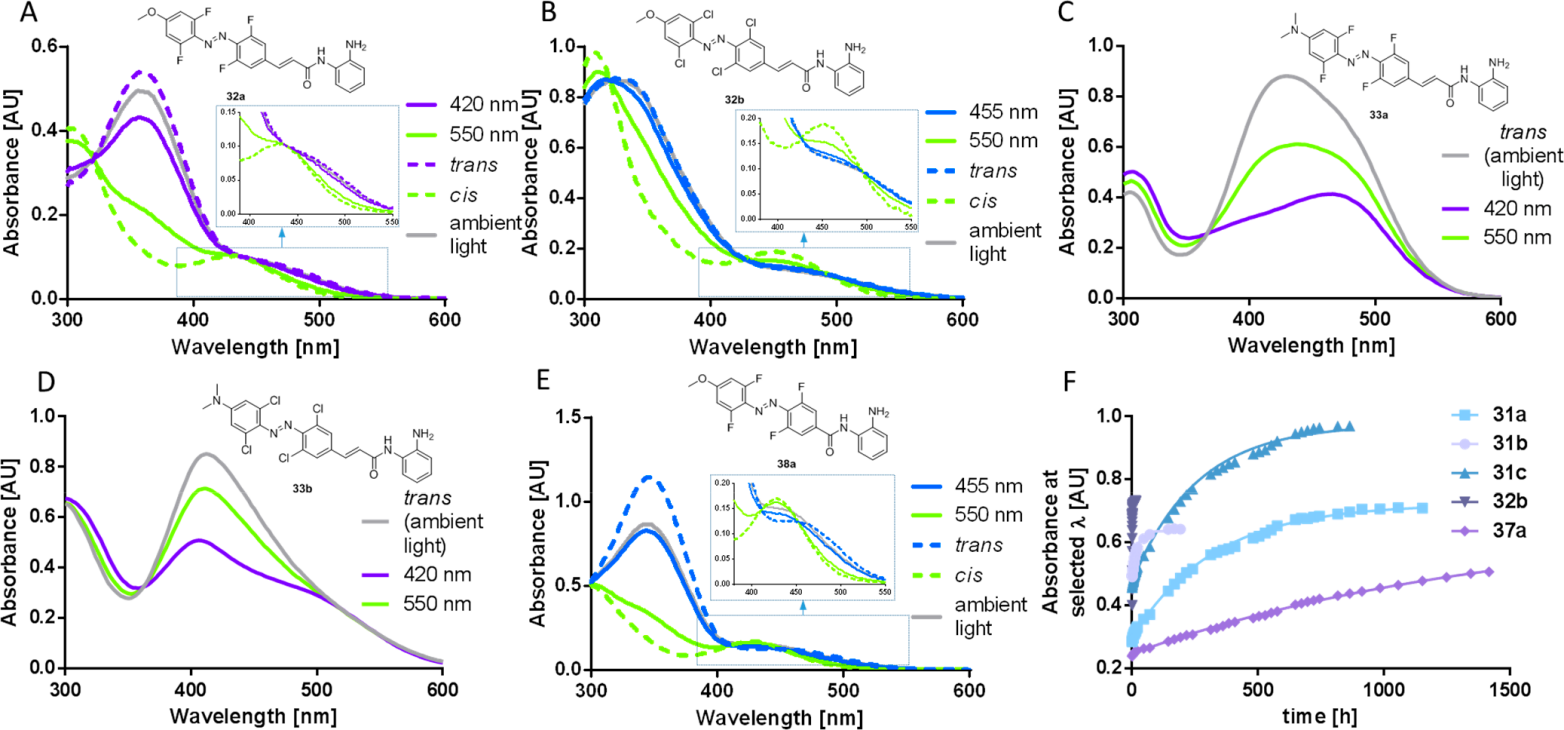
UV–vis spectra of halogenated compounds under different light conditions: (A) **32a**, (B) **32b**, (C) **33a**, (D) **33b**, and (E) **38e**. *trans* and *cis* were calculated from the compound’s irradiated spectra at two wavelengths and their known *E*/*Z* composition. (F) Half-life estimation of the *cis* isomers after irradiation (420 or 550 nm) following their absorbance at selected wavelengths, in DMSO at room temperature.

All *p*-methoxy tetrahalogenated compounds gave maximum amounts of the *cis* isomer upon illumination with green light (550 nm), and back-isomerization to *trans* proceeded with blue light (455 nm) (Figure 4). Conversely, difluoro **32d** and dichloro **32e** gave an absorption profile similar to that of the corresponding nonsubstituted **11** (Figures S12 and S13) but had slightly longer half-lives (Table 3).

**Table 3:**
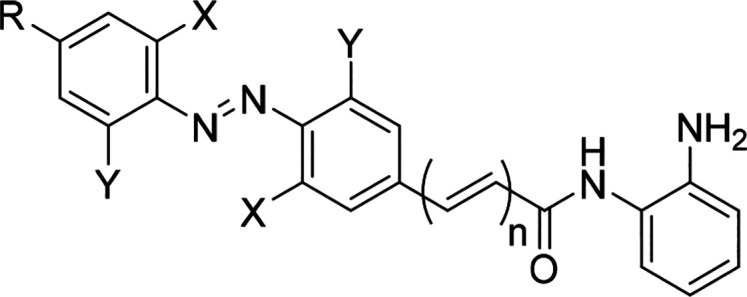
Photochemical Properties of *ortho*-Halogenated Compounds

compound	R	X	Y	*n*	λ_max_*trans* (nm)^a^	λ_max_*cis* (nm)^a^	λ_*cis*_ (nm)^a^	λ_*trans*_ (nm)^a^	*t*_1/2_^b^	% *cis* at λ_*cis*_^c^	% *trans* at λ_*trans*_^c^
**32a**	OMe	F	F	1	358	434	550	455	9.3 days	77	74
**32b**	OMe	Cl	Cl	1	338	452	550	455	13.8 h	45	88
**32c**	OMe	F	Cl	1	358	442	550	455	7.4 days	68	85
**32d**	OMe	F	H	1	388	448	380	550	9.9 h	67	59
**32e**	OMe	Cl	H	1	396	464	380	455	4.3 h	64	62
**33a**	NMe_2_	F	F	1	430		420	550	5.4 h	–	–
**33b**	NMe_2_	Cl	Cl	1	412		420	550	8.1 min	–	–
**38a**	OMe	F	F	0	348	428	550	455	42 days^d^/4.7 days^e^	84	67
**38b**	OMe	Cl	Cl	0	336	452	550	455	8.3 h	46	84
**39**^f^	OMe	F	F	1	358	438	550	455	9.1 days	72	83

a Determined by measuring UV–vis spectra at 25–100 μM in DMSO.

b Determined by measuring UV–vis spectra at 25–100 μM in DMSO over time at room temperature after illumination with λ_*cis*_.

c Determined by HPLC at the isosbestic point of each isomeric pair (290–336 nm) after illumination of a 100 μM solution in DMSO with either λ_*cis*_ or λ_*trans*_.

d Estimation, full back-isomerization not reached.

e In DMEM with 0.1% DMSO at 37 °C, measured by HPLC.

f With HA instead of OAA.

As expected, tetrafluorination had a pronounced effect on the rate of back-isomerization, with an increase in the half-life of up to 72-fold. The fluorine atoms lower the n orbital of the *Z* isomer, hence hugely increasing its thermal stability.^37^ The incorporation of difluoro–dichloro also led to a large 57-fold increase, while tetrachlorination had a small effect. Also, the presence of the alkene generally increased *t*_1/2_ as evidenced by comparing **32a** to **38a** (Figure 4F), which is consistent with the previous results. Thus, the slowest compound herein is non-alkene tetrafluoro **38a**, with a *t*_1/2_ in DMSO at room temperature of 42 days. When measured under the conditions used for cellular assays (37 °C in DMEM with 0.1% DMSO), the *t*_1/2_ was reduced to 4.7 days.

In terms of the proportion of each isomer at the PSS, the *ortho*-halogenated compounds gave moderate to high percentages of the *cis* isomer (64–84%) under illumination with λ_*cis*_ except for the tetrachloro analogues (**32b** and **38b**), where only 45–46% of the *cis* configuration was generated upon illumination with green light.

Another difference in the halogenated compounds was found in the conditions required to obtain maximum amounts of the more stable *trans* isomer. While for the nonhalogenated inhibitors heating to 60 °C for 20 min was sufficient, the tetrahalogenated compounds required 80 °C for 1–2 h.

**Figure 5:**
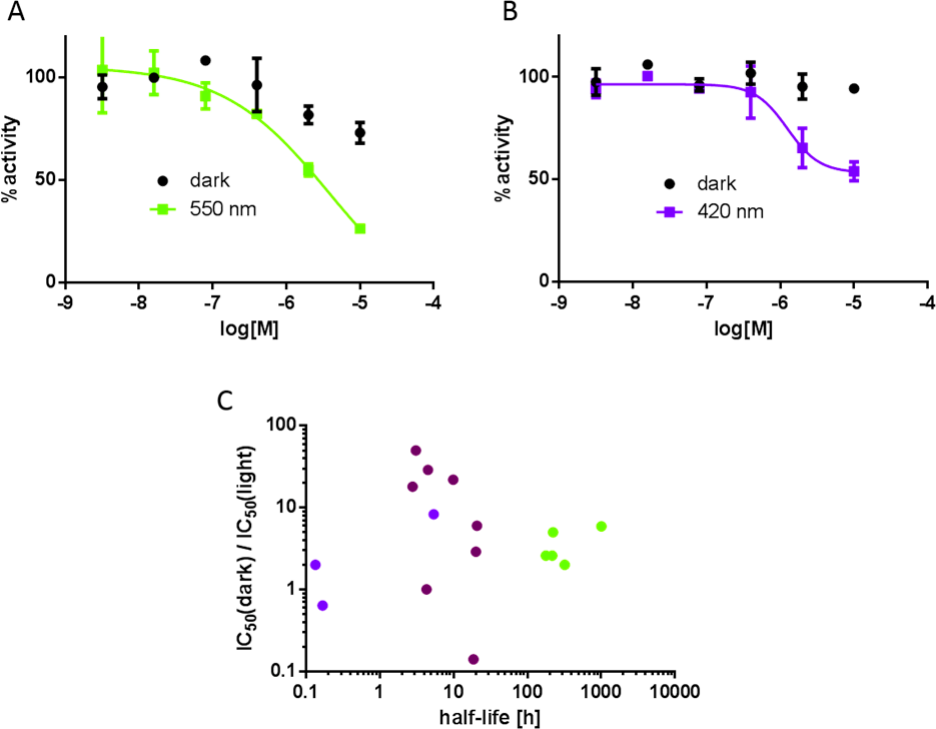
Inhibition of recombinant HDAC1 by the halogenated azobenzenes. Dose–response curves against HDAC1 of (A) **32c** (dark vs 550 nm, 6 mW/cm^2^) and (B) **33a** (dark vs 420 nm, 7 mW/cm^2^). (C) Distribution of all compounds in terms of their relative activity between isomers, half-life of *cis*, and optimal wavelength of isomerization.

We then tested the inhibition of recombinant HDAC1 by these compounds under continuous illumination. Pleasingly, most compounds were more active in the *cis* than in the *trans* form (Table 4). In particular, tetrafluoro **32a** and **33a** (Figure 5B) and difluoro–dichloro **32c** (Figure 5A) had no or little activity at the highest tested concentration (10 μM) in the dark but were active under illumination with IC_50_ values in the low micromolar range. Although these compounds had potencies lower than that of the parent **11**, they have the advantage of being activated under visible light.

**Table 4:** Inhibition of Recombinant HDAC1 by the Halogenated Azobenzenes under Dark or Illumination Conditions, Measured by a Fluorescence Assay

	IC_50_ (μM)		
compound	dark	light	IC_50_(dark)/IC_50_(light)
**32a**	>10	2.0 (550 nm)	>5.0
**32b**	na^a^	≈10 (550 nm)	
**32c**	>10	3.9 (550 nm)	>2.6
**32d**	≈10	0.46 (380 nm)	≈22
**32e**	>10	>10 (380 nm)	
**33a**	na^a^	1.2 (420 nm)	>8.3
**33b**	>10	≈5 (420 nm)	>2
**38a**	≈10	1.7 (550 nm)	≈5.9
**38b**	na^a^	na^a^(550 nm)	
**39**	0.14	0.054 (550 nm)	2.6

a Not active.

The potency of HA **39** was higher than that of OAA **32a**, but **39** exhibited a smaller difference between the two isomers, which agrees with the trends observed above for activity of HAs versus OAAs.

At this point, we had a range of photoswitchable inhibitors of recombinant HDAC1 with higher potencies under illumination and with a range of distinct photochemical properties in terms of their activation wavelengths and half-times (Figure 5C). Given the promise of HDAC inhibitors to induce apoptosis to cancer cells, we proceeded to study the effects of the best analogues in HeLa cells. We first confirmed that representative compounds were able to inhibit HDACs in cells in a whole-cell HDAC assay, which accounts for activity against class 1 and 2b HDAC isoforms (Figure S22). We then proceeded to evaluate the effect of the compounds in a viability assay against HeLa cells. The compounds were either kept in the dark or illuminated before addition onto preseeded cells and then incubated for 24–48 h before the proportion of live cells was measured with an MTS assay.

While the positive control SAHA induced cell death after treatment for 24 h, we found small effects with our compounds at this time point, and clear dose–response curves were obtained only 48 h after compound addition.

**Figure 6:**
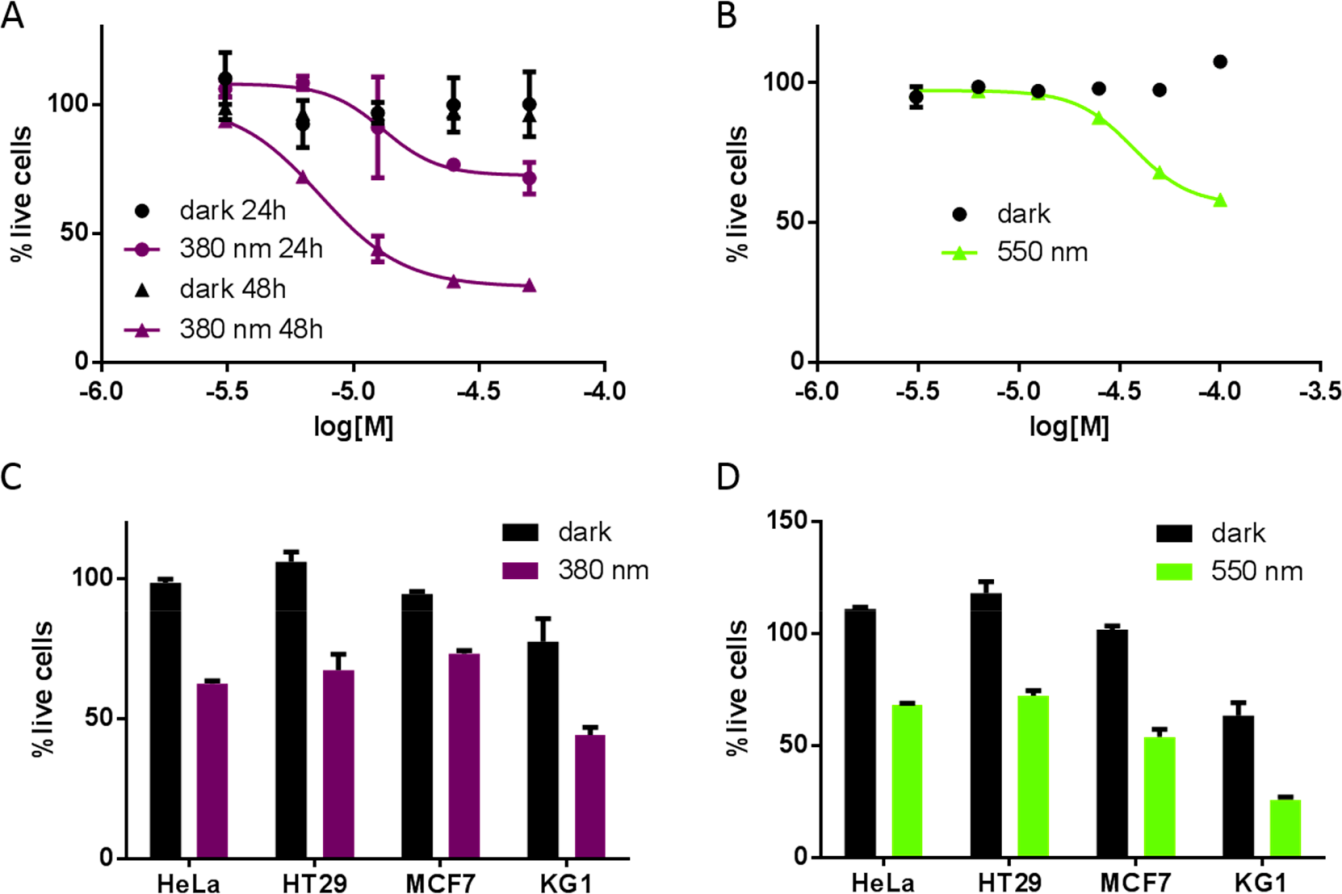
Cellular activity of **20** and **32c**, measured as the percentage of live cells relative to the 0.5% DMSO control. (A) Dose–response curves of **20** on the viability of HeLa cells (MTS assay) under either dark or preillumination (380 nm) conditions for 24 and 48 h; dark n.a., IC_50_ (365 nm, 24 h) = 13 μM, IC_50_ (365 nm, 48 h) = 7.4 μM. (B) Dose–response curve of **32c** on the viability of HeLa cells (MTS assay) under either dark or preillumination (550 nm) conditions for 48 h; dark n.a., IC_50_ (550 nm, 24 h) = 37 μM. Effect of 50 μM (C) **20** and (D) **32c** on HeLa, HT29, MCF7, and KG1 cells under either dark or preillumination (380 or 550 nm) conditions, measured by MTS (HeLa, HT29, and MCF7) and CellTiterGlo (KG1) assays.

From the UV-activatable compounds, OAAs **11** and **20** showed again the best results, with no activity in the dark up to 100 μM, and IC_50_ values under illumination of 12 and 7.4 μM, respectively, with the viability decreasing to 30% (Table 5 and Figure 6A). The observed large effect is remarkable, considering that **11** back-isomerizes under these conditions with a *t*_1/2_ of 40 min. Given that continuous exposure to HDAC inhibitors is required to achieve a full response,^29,38^ these results support the previous findings (Figure 3B) that enzyme engagement in the *cis* form is maintained for longer than its half-life in an enzyme-free solution.

**Table 5:** Effect of Selected Compounds on the Viability of HeLa Cells under Dark or Illumination Conditions, Measured by an MTS Assay

	IC_50_^a^ (μM)		
compound	dark	light	IC_50_(dark)/IC_50_(light)
**17**	≈25^b^	≈25^b^(380 nm)	≈1
**1**	≈12^b^	≈12^b^(380 nm)	≈1
**18**	na^c^	≈19^b^(380 nm)	>5.3
**11**	na^c^	12 (380 nm)	>8.3
**20**	na^c^	7.4 (380 nm)	>14
**21**	na^c^	na^c^(380 nm)	
**32a**	na^c^	37 (550 nm)	>2.7
**32c**	na^c^	37 (550 nm)	>2.7
**32d**	na^c^	na^c^(380 nm)	
**32e**	na^c^	na^c^(380 nm)	
**38a**	>100	29 (550 nm)	>3.4
**39**	6.7	13 (550 nm)	0.52

a IC_50_ corresponds to the concentration of the compound that reduces cell viability by half.

b Estimation, full dose–response not obtained.

c Not active.

Among the halogenated compounds, the findings were again consistent with the results for recombinant HDAC1, with tetrafluoro **32a** and **38a**, and difluoro–dichloro **32c**, inducing cell death only under light conditions.

HAs showed worse results in these cell assays, with no or little difference in activity between dark and illumination conditions.

The faster **2**, **33a**, and **33b** were inactive under both dark and illumination conditions. It is likely that in this case back-isomerization occurred too fast for the *cis* isomer to have enough time to engage with the target, and in this case, constant illumination would be necessary.

HDACis have been proposed as a potential therapeutic strategy against cancer stem cells (CSCs).^39,40^ We selected three cell lines that have a strong CSC phenotype (HT29,^41,42^ MCF7^43−45^ and KG1^46^) and tested the best compounds at 50 μM under dark and illumination conditions. Compounds **11**, **20**, **32a**, **32c**, and **38a** had little or no effect in the dark but significantly reduced the viability of these cells after 48 h when they were preilluminated (Figure 6C,D and Figure S23).

## Conclusions

The ability to localize the effect of anticancer drugs with high spatiotemporal precision could overcome the limitations of current chemotherapy. Photoswitching molecules are particularly attractive, as their therapeutic effect could be modulated with external light. For this purpose, photoswitches with a range of properties will be necessary to shed light on the optimal profile required for clinical progression. One of the properties that will likely be important is the wavelength of activation, as longer wavelengths in the visible spectrum are less harmful and more permeable. In this work, we aimed to contribute to building this toolbox of molecules.

Herein, we chose a well-established target for anticancer drugs, for which photoswitching molecules were already known to facilitate further optimization. Initial SAR studies identified alkene **11**, which had remarkable differences in activity in the recombinant enzyme assays, with the metastable *cis* form being >50-fold more active than the stable *trans* form, with an IC_50_ of 200 nM against HDAC1. It had a half-life of 40 min under cell medium conditions, and it isomerized under near-UV light.

To increase the wavelength of isomerization, the next round of SAR was based on adding halogen atoms *ortho* to the azobenzene group. Tetrafloro **32a** and **33a** and difluoro–dichloro **32c** were particularly attractive. Despite a drop in potency, these were still more active in the *cis* form, with >3–8-fold differences with the *trans* form. Notably, they isomerized under green and blue illumination, respectively, and had half-lives different from that of alkene **11**.

Importantly, we found a good translation of the enzyme activities onto effects in cells, particularly of the benzamide analogues. In four different cancer cell lines, our top compounds caused a significant reduction in viability upon treatment for 48 h after illumination, but no or less activity in the dark.

All in all, we have developed a small library of photoswitchable HDAC inhibitors with a range of biological and photochemical properties. These vary in terms of their activation wavelength, from near-ultraviolet to green wavelengths, and half-lives, from minutes to days. Most examples are more active in the *cis* form than in the *trans* form, with >50-fold differences in recombinant enzyme inhibition assays and >14-fold differences in cell viability assays. We expect that not only the disclosed molecules but also the findings and the explored SAR will be highly valuable in paving the way for further progression of azobenzene-based drugs as cancer therapeutics.

## Experimental Section

### General Synthesis Methods

All reagents were obtained from commercial sources and used without further purification. Reactions were conducted under an inert atmosphere of nitrogen or argon unless not using anhydrous solvents. Anhydrous solvents were obtained from a solvent purification system (PureSolv-EN) and kept under a nitrogen atmosphere. Temperatures of <25 °C were obtained using the following cooling baths: 0 °C ice/water and −5 °C ice/water with NaCl. Concentrations (*c*) in the general procedures refer to the limiting reagent and are given in millimoles per milliliter.

Analytical thin layer chromatography (TLC) was carried out on aluminum sheets coated with silica gel (Macherey-Nagel, 60F, 0.2 mm, ALUGRAM Sil G/UV254). The spots were visualized by UV irradiation (254 nm) and by staining with a KMnO_4_ solution followed by heating.

Flash column chromatography was performed on 60 silica gel (Panreac, 40–63 μm particle size) or with a Biotage Isolera One automated system with Biotage KP-C18-SH cartridges. The eluents are specified in each case.

NMR spectra were recorded on a Varian-Mercury 400 MHz spectrometer. Data are reported as follows: chemical shift, multiplicity (s, singlet; d, doublet; t, triplet; q, quartet; m, multiplet), coupling constant, and integration. Chemical shifts (δ) are reported in parts per million downfield from TMS and are referenced to the residual solvent peak. Coupling constants (*J*) are reported in hertz.

Low-resolution mass spectra (*m*/*z*) were recorded on a Quattro Micro MS detector (Waters). Selected peaks are reported in daltons, and their intensities given as percentages of the base peak. High-resolution mass spectra (HRMS) were recorded on a FIA with an ultra-high-performance liquid chromatography (UPLC) Aquity instrument (Waters) coupled to an LCT Premier Orthogonal Accelerated TOF instrument (Waters). Data from mass spectra were analyzed by electrospray ionization in positive and negative modes using MassLynx version 4.1 (Waters).

Analytical high-performance liquid chromatography (HPLC) was performed on a Thermo Ultimate 3000SD instrument (Thermo Scientific Dionex) coupled to a PDA detector and an LTQ XL ESI-ion trap mass spectrometer (Thermo Scientific) with a Sunfire C18 2.5 μm, 4.6 mm × 50 mm (Waters) column or on an ESI Quattro Micro MS detector (Waters) with a ZORBAX Extend-C18 3.5 μm, 2.1 mm × 50 mm (Agilent) column. The purity of the final compounds was determined to be >95%.

Hydroxamic acid **1**(30) and *o*-aminoanilide **2**(19) were prepared as previously reported.

Syntheses of compounds **19–21** and **39** are shown in Scheme S1.

#### Ethyl (*E*)-3-(4-((*E*)-(4-Hydroxyphenyl)diazenyl)phenyl)acrylate^18^ (**6**)

To a solution of (*E*)-ethyl 3-(4-aminophenyl)acrylate **3** (400 mg, 2.09 mmol) in MeOH (3.0 mL) and HCl (1 M, 5.7 mL, 5,7 mmol) at −5 °C was added a solution of sodium nitrite (171 mg, 2.47 mmol) in water (1.5 mL) dropwise, keeping the temperature below 0 °C. The mixture was then stirred at room temperature for 10 min and cooled to −5 °C. A solution of phenol (179 mg, 1.90 mmol) and KOH (235 mg, 4.18 mmol) in MeOH (7.0 mL) was added dropwise, keeping the temperature below 0 °C. The resulting suspension was stirred at room temperature overnight, diluted with EtOAc, washed with 1 × 1 M HCl and 1× brine, dried over anhydrous Na_2_SO_4_, filtered, and concentrated under reduced pressure to give azobenzene **6** (621 mg, 2.10 mmol, quant) as a brown solid: ^1^H NMR (400 MHz, DMSO-*d*_6_) δ 10.40 (s, 1H), 7.92 (d, *J* = 8.6 Hz, 2H), 7.86–7.78 (m, 4H), 7.72 (d, *J* = 16.0 Hz, 1H), 7.01–6.91 (m, 2H), 6.74 (d, *J* = 16.0 Hz, 1H), 4.21 (q, *J* = 7.1 Hz, 2H), 1.27 (t, *J* = 7.1 Hz, 3H); *m*/*z* (ESI+) 297.3 (MH^+^, 100%).

#### Ethyl (*E*)-3-(4-((*E*)-(4-Methoxyphenyl)diazenyl)phenyl)acrylate^18^ (**8**)

To a solution of phenol **6** (563 mg, 1.90 mmol) in acetone (28.4 mL) were added K_2_CO_3_ (2.63 g, 19.0 mmol) and methyl iodide (950 μL, 15.2 mmol), and the mixture was heated to 50 °C. After 2 h, the mixture was concentrated *in vacuo*, diluted with water, and filtered to give **8** (503 mg, 1.62 mmol, 85%) as a red solid: ^1^H NMR (400 MHz, CDCl_3_) δ 7.98–7.90 (m, 2H), 7.93–7.86 (m, 2H), 7.73 (d, *J* = 16.1 Hz, 1H), 7.69–7.62 (m, 2H), 7.07–6.97 (m, 2H), 6.51 (d, *J* = 16.0 Hz, 1H), 4.28 (q, *J* = 7.1 Hz, 2H), 3.91 (s, 3H), 1.35 (t, *J* = 7.1 Hz, 3H); *m*/*z* (ESI+) 311.3 (MH^+^, 100%).

#### (*E*)-3-(4-((*E*)-(4-Methoxyphenyl)diazenyl)phenyl)acrylic Acid (**9**)

To a solution of ethyl ester **8** (503 mg, 1.62 mmol) in ethanol (3.2 mL) was added NaOH (10 wt %, 1.3 mL, 3.2 mmol). The mixture was stirred at room temperature for 8 h, acidified with 1 M HCl, filtered, washed with water, and dried to give acid **9** (398 mg, 1.41 mmol, 87%) as a brown solid: ^1^H NMR (400 MHz, DMSO-*d*_6_) δ 7.95–7.82 (m, 6H), 7.66 (d, *J* = 16.0 Hz, 1H), 7.18–7.09 (m, 2H), 6.64 (d, *J* = 15.9 Hz, 1H), 3.87 (s, 3H); ^13^C NMR (101 MHz, DMSO-*d*_6_) δ 167.4, 162.3, 152.7, 146.3, 142.9, 136.5, 129.4, 124.8, 122.8, 120.6, 114.7, 55.7; *m*/*z* (ESI+) 283.3 (MH^+^, 100%); HRMS (*m*/*z*) [M – H]^−^ calcd for C_16_H_13_N_2_O_3_^–^ 281.0923, found 281.0932.

#### (*E*)-*N*-(2-Aminophenyl)-3-(4-((*E*)-(4-methoxyphenyl)diazenyl)phenyl)acrylamide (**11**)

To a solution of acid **9** (100 mg, 0.354 mmol) in anhydrous DMF (1.8 mL) were added benzene-1,2-diamine (38 mg, 0.35 mmol), DIPEA (93 μL, 0,53 mmol), EDC (102 mg, 0.531 mmol), and HOBt (81 mg, 0.53 mmol), and the mixture was stirred at room temperature overnight, diluted with water, extracted with 3× DCM, dried over anhydrous Na_2_SO_4_, washed with 2× brine/water, filtered, and concentrated under reduced pressure. The crude product was purified by flash column chromatography (10% to 90% EtOAc in hexane) to give benzamide **11** (72 mg, 0.19 mmol, 55%) as an orange solid: mp 200.1–200.3 °C; ^1^H NMR (400 MHz, DMSO-*d*_6_) δ 9.45 (s, 1H), 7.96–7.88 (m, 4H), 7.82 (d, *J* = 8.4 Hz, 2H), 7.64 (d, *J* = 15.7 Hz, 1H), 7.37 (d, *J* = 7.0 Hz, 1H), 7.19–7.11 (m, 2H), 7.02 (d, *J* = 15.7 Hz, 1H), 6.93 (t, *J* = 7.6 Hz, 1H), 6.76 (dd, *J* = 8.0, 1.5 Hz, 1H), 6.59 (td, *J* = 7.6, 1.5 Hz, 1H), 4.98 (s, 2H), 3.88 (s, 3H); ^13^C NMR (101 MHz, DMSO-*d*_6_) δ 163.3, 162.3, 152.4, 146.3, 141.6, 138.5, 137.2, 128.8, 125.9, 124.8, 124.7, 123.8, 123.4, 123.0, 116.3, 116.0, 114.7, 55.7; *m*/*z* (ESI+) 373.3 (MH^+^, 15%); HRMS (*m*/*z*) [M – H]^−^ calcd for C_22_H_19_N_4_O_2_^–^ 371.1503, found 371.1513.

#### (*E*)-Ethyl 3-(4-((*E*)-(4-(Dimethylamino)phenyl)diazenyl)phenyl)acrylate (**7**)

To a solution of (*E*)-ethyl 3-(4-aminophenyl)acrylate **3** (400 mg, 2.09 mmol) in MeOH (3.0 mL) and HCl (1 M, 5.7 mL, 5.7 mmol) at −5 °C was added a solution of sodium nitrite (171 mg, 2.47 mmol) in water (1.5 mL) dropwise, keeping the temperature below 0 °C. The mixture was then stirred at room temperature for 10 min and cooled to −5 °C. A solution of *N*,*N*-dimethylaniline (240 μL, 1.90 mmol) and potassium hydroxide (235 mg, 4.18 mmol) in MeOH (7.0 mL) was added dropwise, keeping the temperature below 0 °C. The resulting suspension was stirred at room temperature overnight, diluted with EtOAc, washed with 1 × 1 M HCl and 1× brine, dried over anhydrous Na_2_SO_4_, filtered, and concentrated under reduced pressure. The crude product was purified by flash column chromatography (5% to 15% EtOAc in hexane) to give azobenzene **7** (270 mg, 0.835 mmol, 44%) as a red solid: ^1^H NMR (400 MHz, CDCl_3_) δ 7.91 (d, *J* = 8.6 Hz, 2H), 7.86 (d, *J* = 8.5 Hz, 2H), 7.72 (d, *J* = 16.0 Hz, 1H), 7.67–7.58 (m, 2H), 6.77 (d, *J* = 9.4 Hz, 2H), 6.48 (d, *J* = 16.0 Hz, 1H), 4.28 (q, *J* = 7.1 Hz, 2H), 3.11 (s, 6H), 1.35 (t, *J* = 7.1 Hz, 3H); ^13^C NMR (101 MHz, CDCl_3_) δ 167.0, 154.0, 152.8, 143.9, 143.7, 135.2, 128.9, 125.5, 122.7, 118.6, 111.7, 60.6, 40.4, 14.4; *m*/*z* (ESI+) 324.3 (MH^+^, 100%); HRMS (ESI+) [MH]^+^ calcd for C_19_H_22_N_3_O_2_^+^ 324.1704, found 324.1707.

#### (*E*)-3-(4-((*E*)-(4-(Dimethylamino)phenyl)diazenyl)phenyl)acrylic Acid (**10**)

To a suspension of ethyl ester **7** (489 mg, 1.51 mmol) in ethanol (3.0 mL) was added NaOH (10 wt %, 1.2 mL, 3.0 mmol). The mixture was stirred at room temperature overnight, acidified with 1 M HCl, filtered, and washed with water to give a brown solid, which shows 36% remaining starting material. The solid was diluted with ethanol (3.0 mL) and treated with NaOH (10 wt %, 1.2 mL, 3.0 mmol). The mixture was stirred at room temperature overnight, acidified with 1 M HCl, and filtered, and the solid rediluted in MeOH and concentrated *in vacuo* to give acid **10** (88 mg, 0.30 mmol, 20%) as a brown solid: ^1^H NMR (400 MHz, DMSO-*d*_6_) δ 7.88–7.72 (m, 6H), 7.64 (d, *J* = 16.0 Hz, 1H), 6.90–6.78 (m, 2H), 6.60 (d, *J* = 16.0 Hz, 1H), 3.07 (s, 6H); ^13^C NMR (101 MHz, DMSO-*d*_6_) δ 167.5, 153.2, 152.8, 143.1, 142.7, 135.2, 129.3, 125.1, 122.2, 119.8, 111.6, 39.9; *m*/*z* (ESI+) 296.3 (MH^+^, 100%); HRMS (ESI+) [M – H]^−^ calcd for C_17_H_16_N_3_O_2_^–^ 294.1247, found 294.1248.

#### (*E*)-*N*-(2-Aminophenyl)-3-(4-((*E*)-(4-(dimethylamino)phenyl)diazenyl)phenyl)acrylamide (**12**)

To a solution of acid **10** (62 mg, 0.21 mmol) in anhydrous DMF (1.0 mL) were added benzene-1,2-diamine (23 mg, 0.21 mmol), DIPEA (55 μL, 0.32 mmol), EDC (60 mg, 0.32 mmol), and HOBT (48 mg, 0.32 mmol). The mixture was stirred at room temperature overnight, diluted with water, extracted with 3× DCM, dried over anhydrous Na_2_SO_4_, washed with 2× brine/water, filtered, and concentrated under reduced pressure. The crude product was purified by flash column chromatography (10% to 100% EtOAc in hexane) to give benzamide **12** (25 mg, 0.065 mmol, 31%) as an orange solid: mp 218.1–218.9 °C; ^1^H NMR (400 MHz, DMSO-*d*_6_) δ 9.43 (s, 1H), 7.87–7.73 (m, 6H), 7.61 (d, *J* = 15.7 Hz, 1H), 7.37 (dd, *J* = 8.0, 1.5 Hz, 1H), 6.98 (d, *J* = 15.7 Hz, 1H), 6.95–6.89 (m, 1H), 6.88–6.81 (m, 2H), 6.76 (dd, *J* = 8.0, 1.5 Hz, 1H), 6.59 (td, *J* = 7.5, 1.5 Hz, 1H), 4.97 (s, 2H), 3.08 (s, 6H); ^13^C NMR (101 MHz, DMSO-*d*_6_) δ 163.4, 152.9, 152.7, 142.7, 141.6, 138.8, 135.9, 128.7, 125.8, 125.0, 124.7, 123.5, 123.1, 122.5, 116.3, 116.0, 111.6, 39.9; *m*/*z* (ESI^+^) 386.3 (MH^+^, 100%); HRMS (ESI+) [MH]^+^ calcd for C_23_H_24_N_5_O^+^ 386.1985, found 386.1975.

#### 4-Nitrosobenzoic Acid^47^ (**14**)

To a suspension of 4-aminobenzoic acid **13** (1.00 g, 7.29 mmol) in DCM (11.2 mL) was added a solution of Oxone (8.97 g, 14.6 mmol) in water (44.9 mL). The resulting suspension was stirred at room temperature for 1 h, filtered, and dried to give 4-nitrosobenzoic acid (1.05 g, 6.94 mmol, 95%) as a yellow solid: ^1^H NMR (400 MHz, DMSO-*d*_6_) δ 8.30–8.23 (m, 1H), 8.07–7.99 (m, 1H); *m*/*z* (ESI+) 152.2 (MH^+^, 100%).

#### (*E*)-4-((4-Methoxyphenyl)diazenyl)benzoic Acid^48^ (**16**)

A solution of 4-methoxyaniline (204 mg, 1.65 mmol) and nitroso **14** (250 mg, 1.65 mmol) in DCM (10.3 mL) and acetic acid (10.3 mL) was stirred at 35 °C overnight. Then it was cooled o 0 °C and filtered to give azobenzene **16** (226 mg, 0.882 mmol, 53%) as a red solid: ^1^H NMR (400 MHz, DMSO-*d*_6_) δ 8.15–8.08 (m, 2H), 7.97–7.93 (m, 2H), 7.93–7.89 (m, 2H), 7.24–7.05 (m, 2H), 3.89 (s, 3H); *m*/*z* (ESI+) 257.2 (MH^+^, 100%).

#### (*E*)-4-((4-Methoxyphenyl)diazenyl)-*N*-((tetrahydro-2*H*-pyran-2-yl)oxy)benzamide (**S1**)

To a solution of acid **16** (80 mg, 0.31 mmol) in anhydrous DMF (1.6 mL) were added *O*-(tetrahydro-2*H*-pyran-2-yl)hydroxylamine (40 mg, 0.34 mmol), DIPEA (82 μL, 0.47 mmol), EDC (90 mg, 0.47 mmol), and HOBt (72 mg, 0,47 mmol). The mixture was stirred at room temperature overnight, diluted with water, extracted with 3× DCM, washed with a 1:1 brine/water mixture, dried over anhydrous Na_2_SO_4_, filtered, and concentrated under reduced pressure. The crude product was purified by flash column chromatography (20% to 30% EtOAc in hexane) to give **S1** (61 mg, 0.17 mmol, 55%) as a red solid: ^1^H NMR (400 MHz, DMSO-*d*_6_) δ 11.81 (s, 1H), 7.99–7.92 (m, 4H), 7.91 (d, *J* = 8.6 Hz, 2H), 7.20–7.12 (m, 2H), 5.03 (s, 1H), 4.08 (d, *J* = 10.1 Hz, 1H), 3.88 (s, 3H), 3.62–3.48 (m, 1H), 1.81–1.67 (app s, 3H), 1.61–1.48 (m, 3H); ^13^C NMR (101 MHz, DMSO-*d*_6_) δ 163.6, 162.5, 153.7, 146.2, 133.8, 128.6, 125.0, 122.2, 114.8, 101.0, 61.4, 55.8, 27.9, 24.7, 18.3; *m*/*z* (ESI+) 356.2 (MH^+^, 30%); HRMS (ESI-) [M – H]^−^ calcd for C_19_H_20_N_3_O_4_^–^ 354.1459, found 354.1443.

#### (*E*)-*N*-Hydroxy-4-((4-methoxyphenyl)diazenyl)benzamide (**17**)

To a solution of **S1** (46 mg, 0.13 mmol) in MeOH (1.3 mL) was added HCl in 1,4-dioxane (4 M, 1.8 mL, 7.0 mmol). After 30 min, the mixture was concentrated *in vacuo*, and the resulting red solid was diluted with Et_2_O and filtered. The solid was purified by flash column chromatography (0% to 20% MeOH in DCM) to give hydroxamic acid **17** (16 mg, 0,059 mmol, 46%) as an orange solid: mp 198.8–201.4 °C; ^1^H NMR (400 MHz, DMSO-*d*_6_) δ 11.39 (s, 1H), 9.16 (s, 1H), 7.99–7.91 (m, 4H), 7.88 (d, *J* = 8.4 Hz, 2H), 7.16 (d, *J* = 8.9 Hz, 2H), 3.88 (s, 3H); ^13^C NMR (101 MHz, DMSO-*d*_6_) δ 163.4, 162.5, 153.4, 146.2, 134.4, 128.2, 124.9, 122.2, 114.8, 55.8; *m*/*z* (ESI+) 272.3 (MH^+^, 100%); HRMS (ESI-) [M – H]^−^ calcd for C_14_H_12_N_3_O_3_^–^ 270.0885, found 270.0884.

#### *tert*-Butyl (2-Aminophenyl)carbamate^49^ (**S2**)

To a solution of benzene-1,2-diamine (1.00 g, 9.25 mmol) in methanol (18.5 mL) was added di-*tert*-butyl dicarbonate (2.02 g, 9.25 mmol). The solution was stirred at room temperature for 24 h, concentrated under reduced pressure, diluted with brine, extracted with 3× DCM, dried over anhydrous Na_2_SO_4_, filtered, and concentrated under reduced pressure to give protected diamine **S2** (1.90 g, 9.14 mmol, 99%) as a yellow solid: ^1^H NMR (400 MHz, CDCl_3_) δ 7.27 (d, *J* = 6.9 Hz, 1H), 7.01 (ddd, *J* = 8.0, 7.2, 1.5 Hz, 1H), 6.87–6.78 (m, 2H), 6.27 (s, 1H), 1.51 (s, 9H); *m*/*z* (ESI+) 356.2 (MH^+^, 2%).

#### *tert*-Butyl (*E*)-(2-(4-((4-Methoxyphenyl)diazenyl)benzamido)phenyl)carbamate (**S3**)

To a solution of acid **16** (80 mg, 0.31 mmol) in anhydrous DMF (1.6 mL) were added *tert*-butyl (2-aminophenyl)carbamate **S2** (65 mg, 0,31 mmol), DIPEA (82 μL, 0.47 mmol), EDC (90 mg, 0.47 mmol), and HOBt (72 mg, 0.47 mmol). The mixture was stirred at room temperature overnight, diluted with water, extracted with 3× DCM, dried over anhydrous Na_2_SO_4_, washed with a 1:1 brine/water mixture, dried over anhydrous Na_2_SO_4_, filtered, and concentrated under reduced pressure. The crude product was purified by flash column chromatography (5% to 20% EtOAc in hexane) to give **S3** (51 mg, 0.11 mmol, 37%) as a red solid: ^1^H NMR (400 MHz, CDCl_3_) δ 8.10 (d, *J* = 8.6 Hz, 2H), 8.01–7.90 (m, 4H), 7.82 (d, *J* = 8.0 Hz, 1H), 7.22 (d, *J* = 7.3 Hz, 2H), 7.16 (dd, *J* = 6.8, 1.3 Hz, 1H), 7.07–6.99 (m, 2H), 6.88 (s, 1H), 3.91 (s, 3H), 1.53 (s, 9H); ^13^C NMR (101 MHz, CDCl_3_) δ 165.1, 162.7, 154.9, 154.8, 147.1, 135.5, 131.1, 129.9, 128.5, 126.3, 126.2, 126.0, 125.3, 124.6, 122.8, 114.5, 81.7, 55.8, 28.4; *m*/*z* (ESI+) 447.3 (MH^+^, 40%); HRMS (ESI-) [M – H]^−^ calcd for C_25_H_25_N_4_O_4_^–^ 445.1857, found 445.1881.

#### (*E*)-*N*-(2-Aminophenyl)-4-((4-methoxyphenyl)diazenyl)benzamide (**18**)

To a solution of Boc-protected **S3** (47 mg, 0.10 mmol) in DCM (2.1 mL) was added TFA (405 μL, 5.26 mmol). The mixture was stirred at room temperature for 2 h and concentrated *in vacuo*. The resulting red solid was diluted with Et_2_O and filtered. The solid was purified by flash column chromatography (EtOAc in hexane) to give benzamide **18** (24 mg, 0.069 mmol, 66%) as an orange solid: mp 271.2–271.3 °C; ^1^H NMR (400 MHz, DMSO-*d*_6_) δ 9.82 (s, 1H), 8.18 (d, *J* = 8.2 Hz, 2H), 8.01–7.85 (m, 4H), 7.18 (t, *J* = 7.9 Hz, 3H), 6.98 (q, *J* = 6.4, 5.1 Hz, 1H), 6.80 (d, *J* = 8.0 Hz, 1H), 6.61 (t, *J* = 7.6 Hz, 1H), 4.96 (s, 2H), 3.89 (s, 3H); ^13^C NMR (101 MHz, DMSO-*d*_6_) δ 164.6, 162.5, 153.6, 146.2, 143.1, 136.2, 129.1, 126.8, 126.7, 125.0, 123.1, 122.0, 116.2, 116.1, 114.8, 55.8; *m*/*z* (ESI+) 347.3 (MH^+^, 20%); HRMS (ESI+) [MH]^+^ calcd for C_20_H_19_N_4_O_2_^+^ 347.1503, found 347.1503.

#### (*E*)-4-((4-(Dimethylamino)phenyl)diazenyl)-*N*-((tetrahydro-2*H*-pyran-2-yl)oxy)benzamide (**S4**)

To a solution of 4-dimethylaminoazobenzene-4′-carboxylic acid (200 mg, 0.743 mmol) in anhydrous DMF were added DIPEA (650 μL, 3.7 mmol), EDC (427 mg, 2.23 mmol), *O*-(tetrahydro-2*H*-pyran-2-yl)hydroxylamine (96 mg, 0.82 mmol), and HOBt (57 mg, 0.37 mmol). The mixture was stirred at room temperature overnight, diluted with water, extracted with 3× DCM, washed with 1× NaHCO_3_ and 1× brine, dried over anhydrous Na_2_SO_4_, filtered, and concentrated under reduced pressure. The crude product was purified by flash column chromatography (20% to 50% EtOAc in hexane) to give **S4** (179 mg, 0.486 mmol, 65%) as a red solid: ^1^H NMR (400 MHz, DMSO-*d*_6_) δ 11.76 (s, 1H), 7.94–7.88 (m, 2H), 7.86–7.79 (m, 4H), 6.89–6.81 (m, 2H), 5.02 (s, 1H), 4.14–4.01 (m, 1H), 3.61–3.47 (m, 1H), 3.08 (s, 6H), 1.74 (s, 3H), 1.66–1.45 (m, 3H); ^13^C NMR (101 MHz, DMSO-*d*_6_) δ 163.7, 154.2, 152.9, 142.6, 132.5, 128.4, 125.2, 121.6, 111.6, 101.0, 61.4, 39.9, 27.9, 24.8, 18.3; *m*/*z* (ESI+) 369.4 (MH+, 100%); HRMS (ESI+) [M – H]^−^ calcd for C_20_H_23_N_4_O_3_^–^ 367.1776, found 367.1776.

#### (*E*)-4-((4-(Dimethylamino)phenyl)diazenyl)-*N*-hydroxybenzamide (**19**)

To a solution of **S4** (150 mg, 0.407 mmol) in MeOH (4.1 mL) was added HCl in 1,4-dioxane (4 M, 1.75 mL, 7.00 mmol). After 30 min, the mixture was concentrated *in vacuo*. The crude product was diluted with Et_2_O, filtered, washed with more Et_2_O, and dried to give hydroxamic acid **19** (76 mg, 0.27 mmol, 66%) as a dark purple solid: mp 164.0–164.2 °C; ^1^H NMR (400 MHz, DMSO-*d*_6_) δ 7.93–7.85 (m, 2H), 7.85–7.77 (m, 4H), 6.90–6.82 (m, 2H), 3.08 (s, 6H); ^13^C NMR (101 MHz, DMSO-*d*_6_) δ 163.5, 153.7, 152.9, 142.6, 133.0, 128.1, 125.4, 121.5, 112.0, 40.0; *m*/*z* (ESI+) 285.3 (MH^+^, 100%); HRMS (ESI+) [M – H]^−^ calcd for C_15_H_15_N_4_O_2_^–^ 283.1190, found 283.1200.

#### *tert*-Butyl (2-((*E*)-3-(4-((*E*)-(4-Methoxyphenyl)diazenyl)phenyl)acrylamido)-4-(thiophen-2-yl)phenyl)carbamate (**S5**)

To a solution of acid **9** (40 mg, 0.14 mmol) in dry DMF (720 μL) were added *tert*-butyl (2-amino-4-(thiophen-2-yl)phenyl)carbamate^38^ (45 mg, 0.16 mmol), DIPEA (37 μL, 0.21 mmol), EDC (41 mg, 0.21 mmol), and HOBt (32 mg, 0.21 mmol). The mixture was stirred at room temperature overnight, diluted with DCM, washed with 3× brine, dried over anhydrous Na_2_SO_4_, filtered, and concentrated under reduced pressure. The crude product was purified by flash column chromatography (5% to 30% EtOAc in hexane) to give amide **S5** (30 mg, 0.054 mmol, 38%) as an orange solid: ^1^H NMR (400 MHz, CDCl_3_) δ 8.60–8.46 (m, 1H), 7.93 (d, *J* = 8.8 Hz, 2H), 7.88 (d, *J* = 8.1 Hz, 2H), 7.84–7.75 (m, 2H), 7.63 (d, *J* = 8.2 Hz, 2H), 7.39 (q, *J* = 8.9 Hz, 2H), 7.23 (d, *J* = 3.6 Hz, 1H), 7.20 (d, *J* = 5.0 Hz, 1H), 7.12 (s, 1H), 7.08–6.96 (m, 3H), 6.62 (d, *J* = 15.6 Hz, 1H), 3.90 (s, 3H), 1.53 (s, 9H); ^13^C NMR (101 MHz, CDCl_3_) δ 164.8, 162.5, 154.4, 153.6, 147.2, 143.3, 141.8, 136.5, 130.1, 129.0, 128.2, 128.0, 125.1, 125.0, 124.6, 123.9, 123.4, 123.2, 123.0, 122.5, 121.6, 114.4, 81.3, 55.8, 28.5; *m*/*z* (ESI+) 555.0 (MH^+^, 100%); HRMS (ESI+) [MH]^+^ calcd for C_31_H_31_N_4_O_4_S^+^ 555.2061, found 555.2086.

#### (*E*)-*N*-(2-Amino-5-(thiophen-2-yl)phenyl)-3-(4-((*E*)-(4-methoxyphenyl)diazenyl)phenyl)acrylamide (**20**)

To a solution of Boc-protected **S5** (29 mg, 0.052 mmol) in DCM (1.0 mL) was added TFA (120 μL, 1.57 mmol). The mixture was stirred at room temperature for 2 h, basified with saturated aqueous NaHCO_3_, extracted with 3× EtOAc, dried over anhydrous Na_2_SO_4_, filtered, and concentrated under reduced pressure to give an orange solid. The crude product was purified by C18 column chromatography to give **20** (15 mg, 0.033 mmol, 63%) as an orange solid: mp 199.5–200.5 °C; ^1^H NMR (400 MHz, DMSO-*d*_6_) δ 9.53 (s, 1H), 7.98–7.90 (m, 4H), 7.83 (d, *J* = 8.4 Hz, 2H), 7.73 (d, *J* = 2.1 Hz, 1H), 7.67 (d, *J* = 15.8 Hz, 1H), 7.37 (d, *J* = 5.0 Hz, 1H), 7.26 (dd, *J* = 8.4, 2.3 Hz, 1H), 7.23 (d, *J* = 3.7 Hz, 1H), 7.16 (d, *J* = 9.0 Hz, 2H), 7.09–6.99 (m, 2H), 6.80 (d, *J* = 8.3 Hz, 1H), 5.25 (s, 2H), 3.88 (s, 3H); ^13^C NMR (101 MHz, DMSO-*d*_6_) δ 163.5, 162.3, 152.4, 146.3, 144.4, 141.4, 138.8, 137.2, 128.8, 128.2, 124.8, 123.7, 123.5, 123.3, 123.0, 123.0, 122.2, 121.9, 121.0, 116.2, 114.7, 55.7; *m*/*z* (ESI+) 455.4 (MH^+^, 45%); HRMS (ESI-) [M – H]^−^ calcd for C_26_H_21_N_4_O_2_S^–^ 453.1391, found 453.1406.

#### Methyl 3-(4-Nitrophenyl)propanoate^50^ (**S6**)

To a suspension of 3-(4-nitrophenyl)propanoic acid (500 mg, 2.56 mmol) in MeOH (10.2 mL) was added sulfuric acid (960 μL, 17.9 mmol). The mixture was heated to 80 °C for 3 h and concentrated *in vacuo*. The residue was diluted with water, extracted with 3× EtOAc, dried over anhydrous Na_2_SO_4_, filtered, and concentrated under reduced pressure to give methyl ester **S6** (547 mg, 2.61 mmol, quant) as a light brown solid: ^1^H NMR (400 MHz, DMSO-*d*_6_) δ 8.17–8.11 (m, 2H), 7.56–7.49 (m, 2H), 3.58 (s, 3H), 2.99 (t, *J* = 7.5 Hz, 2H), 2.71 (t, *J* = 7.5 Hz, 2H). NMR data were in agreement with the reported values.^50^

#### Methyl 3-(4-Aminophenyl)propanoate^18^ (**S7**)

A suspension of nitro **S6** (547 mg, 2.61 mmol) and 10% palladium on carbon (28 mg, 0.026 mmol) in MeOH (52.3 mL) was stirred at room temperature under a hydrogen atmosphere for 5 h. The mixture was then filtered through Celite and concentrated *in vacuo* to give amine **S7** (452 mg, 2.52 mmol, 96%) as a light orange solid: ^1^H NMR (400 MHz, CDCl_3_) δ 7.08–6.90 (m, 2H), 6.67–6.56 (m, 2H), 3.66 (s, 3H), 2.84 (t, *J* = 7.8 Hz, 2H), 2.57 (t, *J* = 7.8 Hz, 2H); *m*/*z* (ESI+) 180.3 (MH^+^, 100%). NMR data were in agreement with the reported values.^51^

#### Methyl (*E*)-3-(4-((4-Hydroxyphenyl)diazenyl)phenyl)propanoate^18^ (**S8**)

To a solution of **S7** (136 mg, 0.760 mmol) in MeOH (1.5 mL) and HCl (2.1 mL, 2.1 mmol) at −5 °C was added a solution of sodium nitrite (62 mg, 0,90 mmol) in water (530 μL) dropwise, keeping the temperature below 0 °C. The mixture was then stirred at room temperature for 10 min and cooled to −5 °C. A solution of phenol (65 mg, 0.69 mmol) and potassium hydroxide (85 mg, 1.52 mmol) in MeOH (2.7 mL) was added dropwise, keeping the temperature below 0 °C. The resulting suspension was stirred at room temperature overnight, diluted with EtOAc, washed with 1 × 1 M HCl and 1× brine, dried over anhydrous Na_2_SO_4_, filtered, and concentrated under reduced pressure to give azobenzene **S8** (210 mg, 0.74 mmol, quant.) as a dark brown solid: ^1^H NMR (400 MHz, DMSO-*d*_6_) δ 10.28 (s, 1H), 7.81–7.75 (m, 2H), 7.75–7.70 (m, 2H), 7.44–7.38 (m, 2H), 6.98–6.90 (m, 2H), 3.59 (s, 3H), 2.93 (t, *J* = 7.6 Hz, 2H), 2.69 (t, *J* = 7.4 Hz, 2H). NMR data were in agreement with the reported values.^18^

#### Methyl (*E*)-3-(4-((4-Methoxyphenyl)diazenyl)phenyl)propanoate (**S9**)

To a solution of phenol **S8** (200 mg, 0.703 mmol) in acetone (10.0 mL) were added methyl iodide (350 μL, 5.63 mmol) and potassium carbonate (972 mg, 7.03 mmol). The mixture was heated to 50 °C for 1 h and concentrated under reduced pressure. The residue was dissolved in EtOAc, washed with 2× brine, dried over anhydrous Na_2_SO_4_, filtered, and concentrated under reduced pressure to give methoxy **S9** (176 mg, 0.590 mmol, 84%) as an orange solid: ^1^H NMR (400 MHz, CDCl_3_) δ 7.95–7.86 (m, 2H), 7.85–7.77 (m, 2H), 7.36–7.29 (m, 2H), 7.05–6.97 (m, 2H), 3.89 (s, 3H), 3.68 (s, 3H), 3.03 (t, *J* = 7.8 Hz, 2H), 2.68 (t, *J* = 7.8 Hz, 2H). NMR data were in agreement with the reported values.^18^

#### (*E*)-3-(4-((4-Methoxyphenyl)diazenyl)phenyl)propanoic Acid (**S10**)

Sodium hydroxide (456 mg, 1.140 mmol) was added to a solution of methyl ester **S9** (170 mg, 0.570 mmol) in MeOH (1.1 mL). The mixture was heated to 50 °C. After 30 min, the mixture was concentrated, diltued with water, acidified with 1 M HCl, filtered, and dried to give acid **S10** (152 mg, 0.535 mmol, 94%) as an orange solid: ^1^H NMR (400 MHz, DMSO-*d*_6_) δ 12.20 (s, 1H), 7.91–7.83 (m, 2H), 7.77 (d, *J* = 8.4 Hz, 2H), 7.42 (d, *J* = 8.5 Hz, 2H), 7.17–7.08 (m, 2H), 3.86 (s, 3H), 2.91 (t, *J* = 7.6 Hz, 2H), 2.60 (t, *J* = 7.6 Hz, 2H); ^13^C NMR (101 MHz, DMSO-*d*_6_) δ 173.7, 161.9, 150.5, 146.2, 144.2, 129.3, 124.5, 122.3, 114.6, 55.7, 34.9, 30.2; *m*/*z* (ESI+) 285.4 (MH^+^, 100%); HRMS (ESI+) [MH]^+^ calcd for C_16_H_17_N_2_O_3_^+^ 285.1234, found 285.1206.

#### (*E*)-*N*-(2-Aminophenyl)-3-(4-((4-methoxyphenyl)diazenyl)phenyl)propanamide (**21**)

To a solution of acid **S10** (50 mg, 0.18 mmol) in anhydrous DMF (880 μL) were added benzene-1,2-diamine (23 mg, 0.21 mmol), DIPEA (46 μL, 0.26 mmol), EDC (51 mg, 0.26 mmol), and HOBt (40 mg, 0.26 mmol). The mixture was stirred at room temperature overnight, diluted with EtOAc, washed with 2× brine, dried over anhydrous Na_2_SO_4_, filtered, and concentrated under reduced pressure. The crude product was purified by C18 column chromatography to give benzamide **21** (12 mg, 0.032 mmol, 18%) as an orange solid: mp 235.0–235.2 °C; ^1^H NMR (400 MHz, DMSO-*d*_6_) δ 9.16 (s, 1H), 7.89 (d, *J* = 8.9 Hz, 2H), 7.80 (d, *J* = 8.3 Hz, 2H), 7.47 (d, *J* = 8.2 Hz, 2H), 7.18–7.09 (m, 3H), 6.89 (td, *J* = 7.6, 1.6 Hz, 1H), 6.70 (dd, *J* = 8.1, 1.5 Hz, 1H), 6.53 (td, *J* = 7.5, 1.5 Hz, 1H), 3.87 (s, 3H), 3.02 (t, *J* = 7.6 Hz, 2H), 2.70 (t, *J* = 7.7 Hz, 2H); ^13^C NMR (101 MHz, DMSO-*d*_6_) δ 170.2, 161.9, 150.4, 146.2, 144.6, 142.0, 129.3, 125.9, 125.4, 124.5, 123.3, 122.3, 116.1, 115.8, 114.6, 55.7, 37.0, 30.9; *m*/*z* (ESI+) 375.4 (MH^+^, 100%); HRMS (ESI+) [MH]^+^ calcd for C_22_H_23_N_4_O_2_^+^ 375.1816, found 375.2101.

#### (*E*)-4-((2,6-Difluoro-4-iodophenyl)diazenyl)-3,5-difluorophenol (**25a**)

A solution of sodium nitrite (133 mg, 1.92 mmol) in water (5.0 mL) was added dropwise to a suspension of 2,6-difluoro-4-iodoaniline **22a** (490 mg, 1.92 mmol) in water (8.0 mL) and concentrated HCl (2.0 mL) at 0 °C. The suspension was stirred at the same temperature for 20 min and then added to a solution of 3,5-difluorophenol **23a** (250 mg, 1.92 mmol) and sodium hydroxide (154 mg, 3.84 mmol) in water (4.0 mL) dropwise, keeping the temperature below 0 °C and basic pH via addition of aqueous 2 M NaOH. The mixture was then stirred at the same temperature for an additional 2 h, acidified with 1 M HCl, extracted with 3× DCM, dried over anhydrous Na_2_SO_4_, filtered, and concentrated under reduced pressure. The crude product was purified by flash column chromatography (10% to 30% EtOAc in hexane) to give azobenzene **25a** (191 mg, 0.482 mmol, 25%) as a red solid: ^1^H NMR (400 MHz, CDCl_3_) δ 7.47–7.38 (m, 2H), 6.55 (d, *J* = 10.7 Hz, 2H); ^13^C NMR (101 MHz, CDCl_3_) δ 160.1 (m), 157.7 (d, *J* = 269 Hz), 155.1 (d, *J* = 263 Hz), 131.9, 126.4, 122.4 (d, *J* = 27 Hz), 100.7 (d, *J* = 23 Hz), 92.8 (d, *J* = 10 Hz); *m*/*z* (ESI+) 397.0 (MH^+^, 100%); HRMS (ESI+) [MH]^+^ calcd for C_12_H_6_N_2_OF_4_I^+^ 396.9455, found 396.9400.

#### (*E*)-1-(2,6-Difluoro-4-iodophenyl)-2-(2,6-difluoro-4-methoxyphenyl)diazene (**27a**)

To a solution of phenol **25a** (191 mg, 0.482 mmol) in acetone (6.9 mL) were added potassium carbonate (666 mg, 4.82 mmol) and methyl iodide (240 μL, 3.86 mmol). The mixture was stirred at 50 °C for 1 h, concentrated under reduced pressure, diluted with EtOAc, washed with 2× brine, dried over anhydrous Na_2_SO_4_, filtered, and concentrated under reduced pressure to give methoxy **27a** (184 mg, 0.449 mmol, 93%) as a brown solid: ^1^H NMR (400 MHz, CDCl_3_) δ 7.46–7.39 (m, 2H), 6.64–6.56 (m, 2H), 3.88 (s, 3H); ^13^C NMR (101 MHz, CDCl_3_) δ 163.1 (t, *J* = 14 Hz), 157.7 (dd, *J* = 262, 7 Hz), 155.1 (dd, *J* = 265, 6 Hz), 131.9 (t, *J* = 10 Hz), 126.2 (t, *J* = 9 Hz), 122.4 (dd, *J* = 24, 3 Hz), 99.1 (dd, *J* = 24, 3 Hz), 92.8, 56.4; *m*/*z* (ESI+) 411.0 (MH^+^, 100%); HRMS (ESI+) [MH]^+^ calcd for C_13_H_8_F_4_IN_2_O^+^ 410.9612, found 410.9604.

#### *tert*-Butyl (*E*)-3-(4-((*E*)-(2,6-Difluoro-4-methoxyphenyl)diazenyl)-3,5-difluorophenyl)acrylate (**28a**)

To a solution of iodo **27a** (180 mg, 0.439 mmol) in anhydrous DMF (1.8 mL) were added tri-*o*-tolylphosphine (13 mg, 0.044 mmol), triethylamine (133 mg, 1.32 mmol), *tert*-butyl acrylate (84 mg, 0.66 mmol), and palladium(II) acetate (4.9 mg, 0.022 mmol). The mixture was degassed with argon for 10 min and heated to 100 °C overnight. The mixture was diluted with EtOAc, washed with 2× brine, dried over anhydrous Na_2_SO_4_, filtered, and concentrated under reduced pressure. The crude product was purified by flash column chromatography (10% to 20% EtOAc in hexane) to give **28a** (128 mg, 0.312 mmol, 71%) as a red solid: ^1^H NMR (400 MHz, CDCl_3_) δ 7.40 (d, *J* = 15.9 Hz, 1H), 7.15–7.05 (m, 2H), 6.58–6.49 (m, 2H), 6.34 (d, *J* = 15.9 Hz, 1H), 3.81 (s, 3H), 1.47 (s, 9H); *m*/*z* (ESI+) 411.3 (MH^+^, 100%); HRMS (ESI+) [MH]^+^ calcd for C_20_H_19_F_4_N_2_O_3_^+^ 411.1326, found 411.1328.

#### (*E*)-3-(4-((*E*)-(2,6-Difluoro-4-methoxyphenyl)diazenyl)-3,5-difluorophenyl)acrylic Acid (**30a**)

A solution of *tert*-butoxy **28a** (112 mg, 0.273 mmol) in DCM (1.4 mL) and TFA (1.4 mL) was stirred at room temperature. After 1 h, the mixture was concentrated under reduced pressure to give acid **30a** as a dark red solid, which was used in the following step without purification: ^1^H NMR (400 MHz, DMSO-*d*_6_) δ 7.77 (d, *J* = 10.5 Hz, 2H), 7.61 (d, *J* = 16.0 Hz, 1H), 7.08–6.99 (m, 2H), 6.80 (d, *J* = 16.0 Hz, 1H), 3.92 (s, 3H).

#### (*E*)-*N*-(2-Aminophenyl)-3-(4-((*E*)-(2,6-difluoro-4-methoxyphenyl)diazenyl)-3,5-difluorophenyl)acrylamide (**32a**)

To a solution of acid **30a** (97 mg, 0.27 mmol) in DMF (1.4 mL) were added benzene-1,2-diamine (35 mg, 0.33 mmol), DIPEA (72 μL, 0.41 mmol), EDC (79 mg, 0.41 mmol), and HOBt (63 mg, 0.41 mmol). The mixture was stirred at room temperature overnight, diluted with EtOAc, washed with 3× brine, dried over anhydrous Na_2_SO_4_, filtered, and concentrated under reduced pressure. The crude product was purified by flash column chromatography (10% to 50% EtOAc in hexane) to give benzamide **32a** (52 mg, 0.12 mmol, 43%) as an orange solid: mp 199.3–200.3 °C. NMR data were collected after heating a DMSO-*d*_6_ solution of the product at 80 °C for 1 h to obtain the *trans* form: ^1^H NMR (400 MHz, DMSO-*d*_6_) δ 9.46 (s, 1H), 7.65–7.53 (m, 3H), 7.37 (dd, *J* = 7.9, 1.5 Hz, 1H), 7.13–6.97 (m, 3H), 6.93 (td, *J* = 7.6, 1.6 Hz, 1H), 6.76 (dd, *J* = 8.0, 1.5 Hz, 1H), 4.97 (s, 2H), 3.91 (s, 3H); ^13^C NMR (101 MHz, DMSO-*d*_6_) δ 163.4 (d, *J* = 14 Hz), 162.7, 156.7 (dd, *J* = 260, 7 Hz), 154.87 (d, *J* = 258 Hz), 141.6, 138.8 (d, *J* = 10 Hz), 136.5, 130.9 (d, *J* = 10 Hz), 126.6, 126.0, 125.0 (t, *J* = 9 Hz), 124.7, 123.2, 116.3, 116.0, 111.9 (d, *J* = 22 Hz), 99.7 (dd, *J* = 24, 3 Hz), 56.8; *m*/*z* (ESI+) 445.2 (MH^+^, 45%); HRMS (ESI+) [MH]^+^ calcd for C_22_H_17_F_4_N_4_O_2_^+^ 445.1282, found 445.1270.

#### (*E*)-3,5-Dichloro-4-((2,6-dichloro-4-iodophenyl)diazenyl)phenol (**25b**)

A solution of sodium nitrite (34 mg, 0.49 mmol) in water (5.0 mL) was added dropwise to a suspension of 2,6-dichloro-4-iodoaniline **22b**(52,53) (141 mg, 0.491 mmol) in water (8.0 mL) and concentrated HCl (520 μL) at 0 °C. The suspension was stirred at the same temperature for 20 min and then added to a solution of 3,5-dichlorophenol **23b** (80 mg, 0.49 mmol) and sodium hydroxide (39 mg, 0.98 mmol) in water (4.0 mL) dropwise, keeping the temperature below 0 °C and basic pH via addition of 2 M NaOH. The mixture was then stirred at the same temperature for an additional 2 h, acidified with 1 M HCl, extracted with 3× DCM, dried over anhydrous Na_2_SO_4_, filtered, and concentrated under reduced pressure. The crude product was purified by flash column chromatography (5% to 10% EtOAc in hexane) to give azobenzene **25b** (111 mg, 0.240 mmol, 49%) as a red solid: ^1^H NMR (400 MHz, DMSO-*d*_6_) δ 8.07 (s, 2H), 7.06 (s, 2H); ^13^C NMR (101 MHz, DMSO-*d*_6_) δ 160.0, 146.9, 137.8, 137.5, 129.8, 126.5, 117.0, 94.7; *m*/*z* (ESI+) 460.9 (MH^+^, 75%); HRMS (ESI+) [MH]^−^ calcd for C_12_H_4_Cl_4_IN_2_O^–^ 458.8128, found 458.8107.

#### (*E*)-1-(2,6-Dichloro-4-iodophenyl)-2-(2,6-dichloro-4-methoxyphenyl)diazene (**27b**)

To a solution of phenol **25b** (107 mg, 0.232 mmol) in acetone (3.3 mL) were added potassium carbonate (320 mg, 2.32 mmol) and methyl iodide (120 μL, 1.85 mmol). The mixture was stirred at 50 °C for 1 h, concentrated under reduced pressure, diluted with EtOAc, washed with 2× brine, dried over anhydrous Na_2_SO_4_, filtered, and concentrated under reduced pressure to give methoxy **27b** (105 mg, 0.221 mmol, 95%) as a brown solid. The crude product was taken to the following step without further purification: ^1^H NMR (400 MHz, CDCl_3_) δ 7.79 (s, 2H), 7.01 (s, 2H), 3.88 (s, 3H); *m*/*z* (ESI+) 475.0 (MH^+^, 90%).

#### *tert*-Butyl (*E*)-3-(3,5-Dichloro-4-((*E*)-(2,6-dichloro-4-methoxyphenyl)diazenyl)phenyl)acrylate (**28b**)

To a solution of iodo **27b** (90 mg, 0.19 mmol) in anhydrous DMF (950 μL) were added tri-*o*-tolylphosphine (5.8 mg, 0.019 mmol), *tert*-butyl acrylate (41 μL, 0.28 mmol), triethylamine (79 μL, 0.57 mmol), and palladium(II) acetate (2.1 mg, 9.5 μmol). The mixture was degassed with argon for 10 min and then heated to 100 °C overnight. The mixture was allowed to cool to room temperature, diluted with EtOAc, washed with 2× brine, dried over anhydrous Na_2_SO_4_, filtered, and concentrated under reduced pressure. The crude product was purified by flash column chromatography (1% to 2% EtOAc in hexane) to give **28b** (45 mg, 0.095 mmol, 50%) as a dark red solid: ^1^H NMR (400 MHz, CDCl_3_) δ 7.56 (s, 2H), 7.47 (d, *J* = 16.0 Hz, 1H), 7.02 (s, 2H), 6.42 (d, *J* = 16.0 Hz, 1H), 3.88 (s, 3H), 1.54 (s, 9H); ^13^C NMR (101 MHz, CDCl_3_) δ 165.5, 160.4, 148.5, 140.6, 140.0, 136.1, 130.5, 128.5, 127.8, 123.5, 115.6, 81.3, 56.2, 28.3; *m*/*z* (ESI+) 475.1 (MH^+^, 70%); HRMS (ESI+) [MH]^+^ calcd for C_20_H_19_Cl_4_N_2_O_3_^+^ 475.0144, found 475.0136.

#### (*E*)-3-(3,5-Dichloro-4-((*E*)-(2,6-dichloro-4-methoxyphenyl)diazenyl)phenyl)acrylic Acid (**30b**)

A solution of *tert*-butyl ester **28b** (58 mg, 0.12 mmol) in DCM (610 μL) and TFA (610 μL) was stirred at room temperature. After 1 h, the mixture was concentrated under reduced pressure to give acid **30b**, which was taken to the following step without purification: ^1^H NMR (400 MHz, DMSO-*d*_6_) δ 8.09 (s, 2H), 7.61 (d, *J* = 16.1 Hz, 1H), 7.34 (s, 2H), 6.81 (d, *J* = 16.0 Hz, 1H), 3.92 (s, 3H); *m*/*z* (ESI+) 418.9 (MH^+^, 45%).

#### (*E*)-*N*-(2-Aminophenyl)-3-(3,5-dichloro-4-((*E*)-(2,6-dichloro-4-methoxyphenyl)diazenyl)phenyl)acrylamide (**32b**)

To a solution of acid **30b** (50 mg, 0.12 mmol) in anhydrous DMF (600 μL) were added benzene-1,2-diamine (16 mg, 0.14 mmol), DIPEA (31 μL, 0.18 mmol), EDC (34 mg, 0.18 mmol), and HOBt (28 mg, 0.18 mmol). The mixture was stirred at room temperature overnight, diluted with EtOAc, washed with 2× brine, dried over anhydrous Na_2_SO_4_, filtered, and concentrated under reduced pressure. The crude product was purified by flash column chromatography (20% to 30% EtOAc in hexane) to give benzamide **32b** (42 mg, 0.082 mmol, 69%) as an orange solid: mp 234.0–234.5 °C; ^1^H NMR (400 MHz, DMSO-*d*_6_) δ 9.40 (s, 1H), 7.94 (s, 2H), 7.58 (d, *J* = 15.7 Hz, 1H), 7.39 (dd, *J* = 7.9, 1.5 Hz, 1H), 7.34 (s, 2H), 7.10 (d, *J* = 15.8 Hz, 1H), 6.93 (ddd, *J* = 8.5, 7.4, 1.6 Hz, 1H), 6.76 (dd, *J* = 8.0, 1.5 Hz, 1H), 6.59 (td, *J* = 7.5, 1.5 Hz, 1H), 4.97 (br s, 2H), 3.92 (s, 3H); ^13^C NMR (101 MHz, DMSO-*d*_6_) δ 162.8, 160.7, 146.8, 141.5, 139.3, 137.5, 135.9, 129.2, 128.4, 126.5, 126.4, 125.9, 124.6, 123.2, 116.3, 116.0, 115.9, 56.7; *m*/*z* (ESI+) 509.1 (MH^+^, 70%); HRMS (ESI+) [MH]^+^ calcd for C_22_H_17_Cl_4_N_4_O_2_^+^ 509.0100, found 509.0098.

#### 2-Chloro-6-fluoro-4-iodoaniline^54^ (**22c**)

To a solution of 2-chloro-6-fluoroaniline (1.00 g, 6.87 mmol) in acetonitrile (13.8 mL) was added *N*-iodosuccinimide (1.55 g, 6.87 mmol) portionwise. The mixture was stirred at room temperature overnight, diluted with water, extracted with 3× hexane, dried over anhydrous Na_2_SO_4_, filtered, and concentrated under reduced pressure. The crude product was purified by flash column chromatography (1% EtOAc in hexane) to give iodo **22c** (540 mg, 1.99 mmol, 29%) together with 6% remaining starting material: ^1^H NMR (400 MHz, CDCl_3_) δ 7.30 (t, *J* = 1.8 Hz, 1H), 7.16 (dd, *J* = 9.7, 1.8 Hz, 1H), 4.04 (s, 2H).

#### (*E*)-3-Chloro-4-((2-chloro-6-fluoro-4-iodophenyl)diazenyl)-5-fluorophenol (**25c**)

A solution of sodium nitrite (118 mg, 1.71 mmol) in water (5.0 mL) was added dropwise to a suspension of iodo **22c** (463 mg, 1.71 mmol) in water (8.0 mL) and concentrated HCl (1.8 mL) at 0 °C. The suspension was stirred at the same temperature for 30 min and then added to a solution of 3-chloro-5-fluorophenol **23c** (250 mg, 1.71 mmol) and sodium hydroxide (136 mg, 3.41 mmol) in water (4.0 mL) dropwise, keeping the temperature below 0 °C and basic pH via addition of aqueous 2 M NaOH. The mixture was then stirred at the same temperature for an additional 2 h, acidified with 1 M HCl, extracted with 3× DCM, dried over anhydrous Na_2_SO_4_, filtered, and concentrated under reduced pressure. The crude product was purified by flash column chromatography (1% to 10% EtOAc in hexane) to give azobenzene **25c** (268 mg, 0.625 mmol, 37%) as a red solid: ^1^H NMR (400 MHz, methanol-*d*_4_) δ 7.80 (t, *J* = 1.7 Hz, 1H), 7.65 (dd, *J* = 9.9, 1.7 Hz, 1H), 6.89 (dd, *J* = 2.6, 1.5 Hz, 1H), 6.63 (dd, *J* = 13.1, 2.6 Hz, 1H); ^13^C NMR (101 MHz, methanol-*d*_4_) δ 162.9 (d, *J* = 14 Hz), 155.7 (d, *J* = 265 Hz), 153.3 (d, *J* = 264 Hz), 140.8 (d, *J* = 9 Hz), 137.6 (d, *J* = 6 Hz), 135.9 (d, *J* = 4 Hz), 133.4, 132.8 (d, *J* = 3 Hz), 126.4 (d, *J* = 23 Hz), 114.6 (d, *J* = 3 Hz), 104.3 (d, *J* = 23 Hz), 93.4 (d, *J* = 9 Hz); *m*/*z* (ESI+) 429.0 (MH^+^, 100%); HRMS (ESI+) [MH]^+^ calcd for C_12_H_6_Cl_2_F_2_IN_2_O^+^ 428.8864, found 428.8860.

#### (*E*)-1-(2-Chloro-6-fluoro-4-iodophenyl)-2-(2-chloro-6-fluoro-4-methoxyphenyl)diazene (**27c**)

To a solution of phenol **25c** (268 mg, 0.625 mmol) in acetone (8.9 mL) were added potassium carbonate (863 mg, 6.25 mmol) and iodomethane (310 μL, 5.00 mmol). The mixture was stirred at 50 °C for 1 h, concentrated under reduced pressure, diluted with water, extracted with 2× DCM, dried over anhydrous Na_2_SO_4_, filtered, and concentrated under reduced pressure to give methoxy **27c** (238 mg, 0.537 mmol, 86%) as a brown solid: ^1^H NMR (400 MHz, CDCl_3_) δ 7.71 (t, *J* = 1.6 Hz, 1H), 7.50 (dd, *J* = 9.6, 1.7 Hz, 1H), 6.97–6.91 (m, 1H), 6.67 (dd, *J* = 12.9, 2.8 Hz, 1H), 3.88 (s, 3H); *m*/*z* (ESI+) 443.0 (MH^+^, 20%); HRMS (ESI+) [MH]^+^ calcd for C_13_H_8_Cl_2_F_2_IN_2_O^+^ 442.9021, found 442.9034.

#### *tert*-Butyl (*E*)-3-(3-Chloro-4-((*E*)-(2-chloro-6-fluoro-4-methoxyphenyl)diazenyl)-5-fluorophenyl)acrylate (**28c**)

DMF (2.1 mL) degassed with argon was added to iodo **27c** (238 mg, 0.537 mmol), tri-*o*-tolylphosphine (16 mg, 0.054 mmol), and palladium(II) acetate (6,0 mg, 0.027 mmol). Then, triethylamine (220 μL, 1.61 mmol) and *tert*-butyl acrylate (120 μL, 0.806 mmol) were added, and the mixture was degassed with argon for 10 min. The mixture was heated to 100 °C overnight, diluted with EtOAc, washed with 2× brine, dried over anhydrous Na_2_SO_4_, filtered, and concentrated under reduced pressure. The crude product was purified by flash column chromatography (1% to 5% EtOAc in hexane) to give **28c** (108 mg, 0.244 mmol, 45%) as a brown oil: ^1^H NMR (400 MHz, CDCl_3_) δ 7.52–7.42 (m, 2H), 7.25 (dd, *J* = 11.2, 1.7 Hz, 1H), 6.95 (dd, *J* = 2.7, 1.5 Hz, 1H), 6.68 (dd, *J* = 12.8, 2.6 Hz, 1H), 6.41 (d, *J* = 15.9 Hz, 1H), 3.89 (s, 3H), 1.54 (s, 9H); ^13^C NMR (101 MHz, CDCl_3_) δ 165.5, 162.0 (d, *J* = 13 Hz), 154.3 (d, *J* = 266 Hz), 153.0 (d, *J* = 262 Hz), 140.3 (d, *J* = 21 Hz), 140.2 (d, *J* = 2 Hz), 137.0 (d, *J* = 9 Hz), 136.5 (d, *J* = 6 Hz), 133.3 (d, *J* = 8 Hz), 132.5 (d, *J* = 4 Hz), 125.6 (d, *J* = 3 Hz), 123.7, 114.7 (d, *J* = 22 Hz), 112.2 (d, *J* = 3 Hz), 102.4 (d, *J* = 24 Hz), 81.4, 56.3, 28.3; *m*/*z* (ESI+) 443.4 (MH^+^, 25%); HRMS (ESI+) [MH]^+^ calcd for C_20_H_19_Cl_2_F_2_N_2_O_3_ 443.0735, found 443.0709.

#### (*E*)-*N*-(2-Aminophenyl)-3-(3-chloro-4-((*E*)-(2-chloro-6-fluoro-4-methoxyphenyl)diazenyl)-5-fluorophenyl)acrylamide (**32c**)

A solution of *tert*-butoxy **28c** (108 mg, 0.244 mmol) in DCM (1.2 mL) and TFA (1.2 mL) was stirred at room temperature for 1 h and then concentrated *in vacuo*.

To a solution of the crude acid mentioned above in DMF (1.2 mL) were added benzene-1,2-diamine (32 mg, 0.29 mmol), DIPEA (64 μL, 0.37 mmol), EDC (70 mg, 0,37 mmol), and HOBt (56 mg, 0.37 mmol). The mixture was stirred at room temperature overnight, diluted with EtOAc, washed with 3× brine, dried over anhydrous Na_2_SO_4_, filtered, and concentrated under reduced pressure. The crude product was purified by flash column chromatography (30% to 35% EtOAc in hexane) to give benzamide **32c** (32 mg, 0.067 mmol, 28%) as an orange solid. NMR data were collected after heating a DMSO-*d*_6_ solution of the product at 80 °C for 1 h to obtain the *trans* form: ^1^H NMR (400 MHz, DMSO-*d*_6_) δ 9.43 (s, 1H), 7.84 (s, 1H), 7.71 (dd, *J* = 11.8, 1.7 Hz, 1H), 7.58 (d, *J* = 15.7 Hz, 1H), 7.37 (dd, *J* = 7.9, 1.5 Hz, 1H), 7.26 (dd, *J* = 2.7, 1.4 Hz, 1H), 7.13 (dd, *J* = 13.4, 2.7 Hz, 1H), 7.08 (d, *J* = 15.8 Hz, 1H), 6.93 (td, *J* = 7.6, 1.6 Hz, 1H), 6.76 (dd, *J* = 8.0, 1.5 Hz, 1H), 6.59 (td, *J* = 7.6, 1.5 Hz, 1H), 4.98 (s, 2H), 3.92 (s, 3H); ^13^C NMR (101 MHz, DMSO-*d*_6_) δ 162.8, 162.3 (d, *J* = 14 Hz), 153.4 (d, *J* = 263 Hz), 152.0 (d, *J* = 259 Hz), 141.6, 138.6 (d, *J* = 10 Hz), 138.4 (d, *J* = 10 Hz), 136.2, 135.1 (d, *J* = 6 Hz), 132.0 (d, *J* = 8 Hz), 131.1, 126.6, 126.0, 125.4 (d, *J* = 3 Hz), 124.6, 123.2, 116.3, 116.0, 115.0 (d, *J* = 21 Hz), 112.6 (d, *J* = 3 Hz), 102.9 (d, *J* = 24 Hz), 56.8; *m*/*z* (ESI+) 477.2 (MH^+^, 100%); HRMS (ESI+) [MH]^+^ calcd for C_22_H_17_Cl_2_F_2_N_4_O_2_^+^ 477.0691, found 477.0769.

#### (*E*)-3-Fluoro-4-((2-fluoro-4-iodophenyl)diazenyl)phenol (**25d**)

A solution of sodium nitrite (62 mg, 0.89 mmol) in water (2.3 mL) was added dropwise to a suspension of 2-fluoro-4-iodoaniline (211 mg, 0.892 mmol) in water (3.8 mL) and concentrated HCl (940 μL) at 0 °C. The suspension was stirred at the same temperature for 20 min and then added to a solution of 3-fluorophenol (100 mg, 0.892 mmol) and sodium hydroxide (71 mg, 1.8 mmol) in water (1.9 mL) dropwise, keeping the temperature below 0 °C and basic pH via addition of aqueous 2 M NaOH. The mixture was then stirred at the same temperature for an additional 2 h, acidified with 1 M HCl, extracted with 3× DCM, dried over anhydrous Na_2_SO_4_, filtered, and concentrated under reduced pressure. The crude product was purified by flash column chromatography (5% to 10% EtOAc in hexane) to give azobenzene **25d** (198 mg, 0.550 mmol, 62%) as a red solid: ^1^H NMR (400 MHz, DMSO-*d*_6_) δ 10.99 (s, 1H), 7.94 (dd, *J* = 10.2, 1.8 Hz, 1H), 7.74–7.69 (m, 1H), 7.67 (d, *J* = 8.9 Hz, 1H), 7.41 (t, *J* = 8.3 Hz, 1H), 6.81 (dd, *J* = 12.7, 2.5 Hz, 1H), 6.78–6.61 (m, 1H); ^13^C NMR (101 MHz, DMSO-*d*_6_) δ 163.6 (d, *J* = 12 Hz), 161.5 (d, *J* = 257 Hz), 158.5 (d, *J* = 260 Hz), 139.7 (d, *J* = 7 Hz), 134.2 (d, *J* = 4 Hz), 133.5 (d, *J* = 7 Hz), 126.0 (d, *J* = 22 Hz), 118.8, 118.7, 112.8 (d, *J* = 2 Hz), 103.5 (d, *J* = 22 Hz), 98.2 (d, *J* = 8 Hz); *m*/*z* (ESI+) 361.1 (MH^+^, 100%); HRMS (ESI+) [MH]^+^ calcd for C_12_H_8_N_2_OF_2_I^+^ 360.9644, found 360.9619.

#### (*E*)-1-(2-Fluoro-4-iodophenyl)-2-(2-fluoro-4-methoxyphenyl)diazene (**27d**)

To a solution of phenol **25d** (175 mg, 0.486 mmol) in acetone (6.9 mL) were added potassium carbonate (672 mg, 4.86 mmol) and methyl iodide (240 μL, 3.89 mmol). The mixture was stirred at 50 °C for 1 h, concentrated under reduced pressure, diluted with EtOAc, washed with 2× brine, dried over anhydrous Na_2_SO_4_, filtered, and concentrated under reduced pressure to give methoxy **27d** (170 mg, 0.454 mmol, 94%) as a red solid, which was used in the following step without further purification.: ^1^H NMR (400 MHz, DMSO-*d*_6_) δ 7.94 (dd, *J* = 10.2, 1.7 Hz, 1H), 7.88–7.55 (m, 2H), 7.42 (t, *J* = 8.3 Hz, 1H), 7.13 (dd, *J* = 12.9, 2.6 Hz, 1H), 6.92 (dd, *J* = 9.1, 2.7 Hz, 1H), 3.88 (s, 3H); ^13^C NMR (101 MHz, DMSO-*d*_6_) δ 164.3 (d, *J* = 11 Hz), 161.4 (d, *J* = 258 Hz), 158.6 (d, *J* = 261 Hz), 139.6 (d, *J* = 7 Hz), 134.3–134.2 (m), 134.2 (m), 126.1 (d, *J* = 22 Hz), 118.8, 118.4, 112.0 (d, *J* = 3 Hz), 102.3 (d, *J* = 23 Hz), 98.8 (d, *J* = 8 Hz), 56.4; *m*/*z* (ESI+) 375.1 (MH^+^, 100%); HRMS (ESI+) [MH]^+^ calcd for C_13_H_10_N_2_OF_2_I^+^ 374.9800, found 374.9804.

#### *tert*-Butyl (*E*)-3-(3-Fluoro-4-((*E*)-(2-fluoro-4-methoxyphenyl)diazenyl)phenyl)acrylate (**28d**)

To a solution of iodo **27d** (125 mg, 0.334 mmol) in anhydrous DMF (1.3 mL) were added tri-*o*-tolylphosphine (10 mg, 0.033 mmol), *tert*-butyl acrylate (73 μL, 0,50 mmol), triethylamine (140 μL, 1.0 mmol), and palladium(II) acetate (3.8 mg, 0.017 mmol). The mixture was degassed with argon for 10 min and then heated to 100 °C overnight. The mixture was allowed to cool to room temperature, diluted with EtOAc, washed with 2× brine, dried over anhydrous Na_2_SO_4_, filtered, and concentrated under reduced pressure. The crude product was purified by flash column chromatography (2% to 5% EtOAc in hexane) to give **28d** (94 mg, 0.25 mmol, 75%) as a red solid: ^1^H NMR (400 MHz, CDCl_3_) δ 7.84 (dd, *J* = 9.4, 8.5 Hz, 1H), 7.78 (t, *J* = 8.0 Hz, 1H), 7.55 (d, *J* = 16.0 Hz, 1H), 7.38 (dd, *J* = 11.3, 1.8 Hz, 1H), 7.34 (dd, *J* = 8.4, 1.8 Hz, 1H), 6.84–6.73 (m, 2H), 6.42 (d, *J* = 16.0 Hz, 1H), 3.89 (s, 3H), 1.54 (s, 9H); ^13^C NMR (101 MHz, CDCl_3_) δ 165.8, 164.3 (d, *J* = 11 Hz), 162.2 (d, *J* = 259 Hz), 160.1 (d, *J* = 258 Hz), 141.6 (d, *J* = 7 Hz), 141.5 (d, *J* = 2 Hz), 138.8 (d, *J* = 8 Hz), 135.6 (d, *J* = 7 Hz), 124.2 (d, *J* = 3 Hz), 122.7, 118.9 (d, *J* = 2 Hz), 118.4, 116.0 (d, *J* = 21 Hz), 111.2 (d, *J* = 3 Hz), 102.0 (d, *J* = 23 Hz), 81.1, 56.1, 28.3; *m*/*z* (ESI+) 375.2 (MH^+^, 100%); HRMS (ESI+) [MH]^+^ calcd for C_20_H_21_F_2_N_2_O_3_^+^ 375.1515, found 375.1550.

#### (*E*)-*N*-(2-Aminophenyl)-3-(3-fluoro-4-((*E*)-(2-fluoro-4-methoxyphenyl)diazenyl)phenyl)acrylamide (**32d**)

A solution of *tert*-butoxy **28d** (36 mg, 0.096 mmol) in DCM (480 μL) and TFA (480 μL) was stirred at room temperature. After 1 h, the mixture was concentrated under reduced pressure.

To a solution of the crude acid mentioned above in anhydrous DMF (480 μL) were added benzene-1,2-diamine (12 mg, 0.11 mmol), DIPEA (25 μL, 0.14 mmol), EDC (28 mg, 0.14 mmol), and HOBt (22 mg, 0.14 mmol). The mixture was stirred at room temperature overnight, diluted with EtOAc, washed with 2× brine, dried over anhydrous Na_2_SO_4_, filtered, and concentrated under reduced pressure. The crude product was purified by C18 column chromatography to give benzamide **32d** (13 mg, 0.032 mmol, 33%) as an orange solid: mp 196.1–200.6 °C; ^1^H NMR (400 MHz, DMSO-*d*_6_) δ 9.45 (s, 1H), 7.74–7.64 (m, 3H), 7.57–7.51 (m, 2H), 7.30 (dd, *J* = 7.9, 1.5 Hz, 1H), 7.09 (dd, *J* = 12.9, 2.7 Hz, 1H), 6.99 (d, *J* = 15.8 Hz, 1H), 6.91–6.81 (m, 2H), 6.68 (dd, *J* = 8.0, 1.5 Hz, 1H), 6.51 (td, *J* = 7.5, 1.5 Hz, 1H), 4.92 (s, 2H), 3.82 (s, 3H); ^13^C NMR (101 MHz, DMSO-*d*_6_) δ 164.3 (d, *J* = 11 Hz), 163.0, 161.4 (d, *J* = 258 Hz), 159.3 (d, *J* = 256.0 Hz), 141.6, 140.2 (d, *J* = 7 Hz), 139.8 (d, *J* = 8 Hz), 137.4 (d, *J* = 2 Hz), 134.5 (d, *J* = 7 Hz), 125.9, 125.4, 124.7, 123.8 (d, *J* = 3 Hz), 123.3, 118.4, 118.1, 116.4 (d, *J* = 20 Hz), 116.2, 116.0, 112.0 (d, *J* = 2 Hz), 102.3 (d, *J* = 23 Hz), 56.4; *m*/*z* (ESI+) 409.3 (MH^+^, 100%); HRMS (ESI+) [MH]^+^ calcd for C_22_H_19_F_2_N_4_O_2_^+^ 409.1471, found 409.1445.

#### (*E*)-3-Chloro-4-((2-chloro-4-iodophenyl)diazenyl)phenol (**25e**)

A solution of sodium nitrite (134 mg, 1.94 mmol) in water (5.0 mL) was added dropwise to a suspension of 2-chloro-4-iodoaniline **22e** (493 mg, 1.94 mmol) in water (8.0 mL) and concentrated HCl (2.0 mL) at 0 °C. The suspension was stirred at the same temperature for 20 min and then added to a solution of 3-chlorophenol **23e** (250 mg, 1.94 mmol) and sodium hydroxide (156 mg, 3.89 mmol) in water (4.0 mL) dropwise, keeping the temperature below 0 °C and basic pH via addition of aqueous 2 M NaOH. The mixture was then stirred at the same temperature for an additional 2 h, acidified with 1 M HCl, extracted with 3× DCM, dried over anhydrous Na_2_SO_4_, filtered, and concentrated under reduced pressure. The crude product was purified by flash column chromatography (5% to 10% EtOAc in hexane) to give azobenzene **25e** (540 mg, 1.37 mmol, 71%) as a red solid: ^1^H NMR (400 MHz, DMSO-*d*_6_) δ 10.97 (s, 1H), 8.10 (d, *J* = 1.8 Hz, 1H), 7.85 (dd, *J* = 8.5, 1.8 Hz, 1H), 7.71 (d, *J* = 9.0 Hz, 1H), 7.38 (d, *J* = 8.5 Hz, 1H), 7.07 (d, *J* = 2.5 Hz, 1H), 6.90 (dd, *J* = 9.0, 2.5 Hz, 1H); ^13^C NMR (101 MHz, DMSO-*d*_6_) δ 162.5, 147.6, 141.1, 138.5, 137.5, 137.1, 134.6, 119.1, 119.0, 116.7, 115.8, 98.6; *m*/*z* (ESI+) 393.0 (MH^+^, 100%); HRMS (ESI+) [MH]^−^ calcd for C_12_H_6_Cl_2_IN_2_O^–^ 390.8907, found 390.8926.

#### (*E*)-1-(2-Chloro-4-iodophenyl)-2-(2-chloro-4-methoxyphenyl)diazene (**27e**)

To a solution of phenol **25e** (466 mg, 1.19 mmol) in acetone (16.9 mL) were added potassium carbonate (1.64 g, 11.9 mmol) and methyl iodide (593 μL, 9.49 mmol). The mixture was stirred at 50 °C for 1 h, concentrated under reduced pressure, diluted with EtOAc, washed with 2× brine, dried over anhydrous Na_2_SO_4_, filtered, and concentrated under reduced pressure to give methoxy **27e** (455 mg, 1.12 mmol, 94%) as a brown solid, which was used in the following step without further purification: ^1^H NMR (400 MHz, CDCl_3_) δ 7.92 (d, *J* = 1.8 Hz, 1H), 7.85 (d, *J* = 9.1 Hz, 1H), 7.67 (dd, *J* = 8.5, 1.8 Hz, 1H), 7.48 (d, *J* = 8.5 Hz, 1H), 7.08 (d, *J* = 2.7 Hz, 1H), 6.88 (dd, *J* = 9.1, 2.7 Hz, 1H), 3.90 (s, 3H); *m*/*z* (ESI+) 407.0 (MH^+^, 100%).

#### *tert*-Butyl (*E*)-3-(3-Chloro-4-((*E*)-(2-chloro-4-methoxyphenyl)diazenyl)phenyl)acrylate (**28e**)

To a solution of iodo **27e** (452 mg, 1.11 mmol) in anhydrous DMF (5.6 mL) were added tri-*o*-tolylphosphine (34 mg, 0.11 mmol), *tert*-butyl acrylate (240 μL, 1.67 mmol), triethylamine (460 μL, 3.33 mmol), and palladium(II) acetate (12 mg, 0.056 mmol). The mixture was degassed with argon for 10 min and then heated to 100 °C overnight. The mixture was allowed to cool to room temperature, diluted with EtOAc, washed with 2× brine, dried over anhydrous Na_2_SO_4_, filtered, and concentrated under reduced pressure. The crude product was purified by flash column chromatography (1% to 2% EtOAc in hexane) to give **28e** (286 mg, 0.702 mmol, 63%) as a red solid: ^1^H NMR (400 MHz, CDCl_3_) δ 7.86 (d, *J* = 9.1 Hz, 1H), 7.77 (d, *J* = 8.4 Hz, 1H), 7.68 (d, *J* = 1.8 Hz, 1H), 7.54 (d, *J* = 15.9 Hz, 1H), 7.46 (dd, *J* = 8.5, 1.9 Hz, 1H), 7.08 (d, *J* = 2.6 Hz, 1H), 6.89 (dd, *J* = 9.1, 2.7 Hz, 1H), 6.43 (d, *J* = 16.0 Hz, 1H), 3.89 (s, 3H), 1.54 (s, 9H); ^13^C NMR (101 MHz, CDCl_3_) δ 165.8, 163.1, 149.4, 143.3, 141.3, 138.6, 138.1, 135.9, 130.0, 126.9, 122.7, 119.3, 118.6, 115.0, 114.3, 81.1, 56.1, 28.3; *m*/*z* (ESI+) 407.2 (MH^+^, 25%); HRMS (ESI+) [MH]^+^ calcd for C_20_H_21_Cl_2_N_2_O_3_^+^ 407.0924, found 407.0925.

#### (*E*)-*N*-(2-Aminophenyl)-3-(3-chloro-4-((*E*)-(2-chloro-4-methoxyphenyl)diazenyl)phenyl)acrylamide (**32e**)

A solution of *tert*-butoxy **28e** (122 mg, 0.300 mmol) in DCM (1.5 mL) and TFA (1.5 mL) was stirred at room temperature. After 1 h, the mixture was concentrated under reduced pressure.

To a solution of the crude acid mentioned above in anhydrous DMF (1.5 mL) were added benzene-1,2-diamine (39 mg, 0.36 mmol), DIPEA (79 μL, 0.45 mmol), EDC (86 mg, 0.45 mmol), and HOBt (69 mg, 0,45 mmol). The mixture was stirred at room temperature overnight, diluted with water, filtered, and dried to give benzamide **32e** (81 mg, 0.18 mmol, 61%) as a red solid: ^1^H NMR (400 MHz, DMSO-*d*_6_) δ 9.47 (s, 1H), 8.02–7.92 (m, 1H), 7.82–7.70 (m, 3H), 7.61 (d, *J* = 15.7 Hz, 1H), 7.37 (dd, *J* = 7.9, 1.6 Hz, 1H), 7.35 (d, *J* = 2.7 Hz, 1H), 7.11 (dd, *J* = 9.1, 2.7 Hz, 1H), 7.06 (d, *J* = 15.8 Hz, 1H), 6.93 (td, *J* = 7.6, 1.6 Hz, 1H), 6.76 (dd, *J* = 8.0, 1.5 Hz, 1H), 6.59 (td, *J* = 7.5, 1.4 Hz, 1H), 4.99 (br s, 2H), 3.91 (s, 3H); ^13^C NMR (101 MHz, DMSO-*d*_6_) δ 163.2, 163.0, 148.0, 142.3, 141.6, 139.2, 137.4, 137.1, 134.6, 130.0, 126.8, 125.9, 125.5, 124.7, 123.3, 118.8, 118.2, 116.3, 116.0, 115.1, 115.0, 56.3; *m*/*z* (ESI+) 441.3 (MH^+^, 90%); HRMS (ESI+) [MH]^+^ calcd for C_22_H_19_Cl_2_N_4_O_2_^+^ 441.0880, found 441.0881.

#### 3,5-Difluoro-*N*,*N*-dimethylaniline (**25a**)

To a solution of 3,5-difluoroaniline (500 mg, 3.87 mmol) in acetonitrile (7.7 mL) were added potassium carbonate (1.34 g, 9.68 mmol) and iodomethane (1.2 mL, 19 mmol). The mixture was heated to 60 °C overnight, cooled to room temperature, diluted with water, extracted with 3× EtOAc, dried over anhydrous Na_2_SO_4_, filtered, and concentrated under reduced pressure to give dimethylaniline **25a** (642 mg, 4.08 mmol, quant) as a yellow solid: ^1^H NMR (400 MHz, CDCl_3_) δ 6.47 (d, *J* = 9.1 Hz, 2H), 6.34 (t, *J* = 9.0 Hz, 1H), 3.00 (s, 6H); *m*/*z* (ESI+) 158.2 (MH^+^, 100%).

#### (*E*)-4-((2,6-Difluoro-4-iodophenyl)diazenyl)-3,5-difluoro-*N*,*N*-dimethylaniline (**26a**)

A solution of sodium nitrite (88 mg, 1.3 mmol) in sulfuric acid (880 μL, 16.5 mmol) was heated to 70 °C and then cooled to 0 °C. To this mixture was added a solution of 2,6-difluoro-4-iodoaniline **22a** (325 mg, 1.27 mmol) in DMF (4.0 mL) and acetic acid (1.5 mL). After the mixture had been stirred for 2 h at 0 °C, a solution of 3,5-difluoro-*N*,*N*-dimethylaniline **24a** (100 mg, 0.636 mmol) was added dropwise. The mixture was stirred for an additional 1 h at 0 °C and at room temperature for 3 days. The mixture was then diluted with DCM, washed with 2× saturated aqueous NaHCO_3_ and 1× brine, dried over anhydrous Na_2_SO4, filtered, and concentrated under reduced pressure. The crude product was purified by flash column chromatography (5% to 10% EtOAc in hexane) to give azobenzene **26a** (108 mg, 0.255 mmol, 40%) as a red solid: ^1^H NMR (400 MHz, CDCl_3_) δ 7.38 (d, *J* = 8.0 Hz, 2H), 6.25 (d, *J* = 13.5 Hz, 2H), 3.08 (s, 6H); ^13^C NMR (100 MHz, CDCl_3_) δ 158.6 (dd, *J* = 260, 8 Hz), 155.1 (dd, *J* = 260, 4 Hz), 153.2 (t, *J* = 14 Hz), 153.6 (m), 132.4 (m), 122.2 (m), 95.2 (m), 90.7 (t, *J* = 10 Hz), 40.4; *m*/*z* (ESI+) 424.1 (MH^+^, 100%); HRMS (ESI+) [MH]^+^ calcd for C_14_H_11_F_4_IN_3_^+^ 423.9928, found 423.9988.

#### *tert*-Butyl (*E*)-3-(4-((*E*)-(4-(Dimethylamino)-2,6-difluorophenyl)diazenyl)-3,5-difluorophenyl)acrylate (**29a**)

To a mixture of iodo **26a** (251 mg, 0.593 mmol), tri-*o*-tolylphosphine (18 mg, 0.059 mmol) and palladium(II) acetate (6,7 mg, 0.030 mmol) under argon were added degassed anhydrous DMF (2.4 mL), triethylamine (250 μL, 1.78 mmol), and *tert*-butyl acrylate (130 μL, 0.890 mmol), and the mixture was degassed with argon for an additional 10 min and then heated to 100 °C overnight. The mixture was diluted with EtOAc, washed with 2× brine, dried over anhydrous Na_2_SO_4_, filtered, and concentrated under reduced pressure. The crude product was purified by flash column chromatography (5% to 15% EtOAc in hexane) to give **29a** (155 mg, 0.366 mmol, 62%) as a red solid: ^1^H NMR (400 MHz, CDCl_3_) δ 7.46 (d, *J* = 15.9 Hz, 1H), 7.14 (d, *J* = 9.4 Hz, 2H), 6.37 (d, *J* = 15.9 Hz, 1H), 6.25 (d, *J* = 13.4 Hz, 2H), 3.08 (s, 6H), 1.53 (s, 9H); ^13^C NMR (101 MHz, CDCl_3_) δ 165.61, 158.6 (dd, *J* = 260, 8 Hz), 155.7 (dd, *J* = 258, 5 Hz), 153.2 (t, *J* = 14 Hz), 140.8 (t, *J* = 3 Hz), 135.9 (t, *J* = 10 Hz), 133.2 (t, *J* = 11 Hz), 123.1, 122.6 (t, *J* = 9 Hz), 111.8 (d, *J* = 24 Hz), 95.2 (dd, *J* = 26, 2 Hz), 81.2, 40.3, 28.3; *m*/*z* (ESI+) 424.3 (MH^+^, 100%); HRMS (ESI+) [MH]^+^ calcd for C_21_H_22_F_4_N_3_O_2_^+^ 424.1648, found 424.1685.

#### (*E*)-*N*-(2-Aminophenyl)-3-(4-((*E*)-(4-(dimethylamino)-2,6-difluorophenyl)diazenyl)-3,5-difluorophenyl)acrylamide (**33a**)

A solution of *tert*-butoxy **29a** (150 mg, 0.354 mmol) in DCM (1.8 mL) and TFA (1.8 mL) was stirred at room temperature. After 1 h, the mixture was concentrated under reduced pressure to give a dark red solid.

To a solution of the crude acid mentioned above in DMF (1.8 mL) were added benzene-1,2-diamine (45 mg, 0.42 mmol), DIPEA (92 μL, 0.52 mmol), EDC (101 mg, 0.525 mmol), and HOBt (80 mg, 0.52 mmol). The mixture was stirred at room temperature overnight, diluted with EtOAc, washed with 3× brine, dried over anhydrous Na_2_SO_4_, filtered, and concentrated under reduced pressure. The crude product was purified by flash column chromatography (30% to 100% EtOAc in hexane) to give benzamide **33a** (127 mg, 0.278 mmol, 79%) as an orange solid: ^1^H NMR (400 MHz, DMSO-*d*_6_) δ 9.42 (s, 1H), 7.62–7.46 (m, 3H), 7.37 (d, *J* = 7.8 Hz, 1H), 7.02 (d, *J* = 15.7 Hz, 1H), 6.93 (t, *J* = 7.6 Hz, 1H), 6.76 (d, *J* = 7.9 Hz, 1H), 6.61–6.56 (m, 3H), 4.97 (s, 2H), 3.09 (s, 6H); ^13^C NMR (101 MHz, DMSO-*d*_6_) δ 162.9, 157.7 (dd, *J* = 258, 9 Hz), 156.3–153.5 (m), 153.5, 141.6, 137.0–136.5 (m, 2 × C), 131.8 (t, *J* = 10 Hz), 125.9, 125.7, 124.7, 123.3, 120.9 (t, *J* = 9 Hz), 116.3, 116.0, 111.7 (d, *J* = 24 Hz), 95.2 (d, *J* = 25 Hz), 40.2; *m*/*z* (ESI+) 458.2 (MH^+^, 100%); HRMS (ESI+) [MH]^+^ calcd for C_23_H_20_F_4_N_5_O^+^ 458.1598, found 458.1562.

#### (*E*)-3,5-Dichloro-4-((2,6-dichloro-4-iodophenyl)diazenyl)-*N*,*N*-dimethylaniline (**26b**)

A solution of sodium nitrite (73 mg, 1.05 mmol) in sulfuric acid (730 μL, 13.7 mmol) was heated to 70 °C and then cooled to 0 °C. To this mixture was added a solution of 2,6-dichloro-4-iodoaniline **22b**(52,53) (303 mg, 1.05 mmol) in DMF (4.0 mL) and acetic acid (1.5 mL). After the mixture had been stirred for 2 h at 0 °C, a solution of 3,5-dichloro-*N*,*N*-dimethylaniline **24b**(55,56) (100 mg, 0.526 mmol) was added dropwise. The mixture was stirred for an additional 1 h at 0 °C and at room temperature over the weekend. The mixture was then diluted with DCM, washed with 2× NaHCO_3_ and 1× brine, dried over anhydrous Na_2_SO_4_, filtered, and concentrated under reduced pressure to give a dark orange oil. The crude product was purified by flash column chromatography (5% to 10% EtOAc in hexane) to give azobenzene **26b** (191 mg, 0.391 mmol, 74%) as a red solid: ^1^H NMR (400 MHz, CDCl_3_) δ 7.74 (s, 2H), 6.71 (s, 2H), 3.08 (s, 6H); ^13^C NMR (101 MHz, CDCl_3_) δ 151.3, 148.6, 137.5, 135.3, 132.6, 128.0, 112.4, 90.6, 40.3; *m*/*z* (ESI+) 488.0 (MH^+^, 70%); HRMS (ESI+) [MH]^+^ calcd for C_14_H_11_ClIN_3_^+^ 487.8746, found 487.8721.

#### *tert*-Butyl (*E*)-3-(3,5-Dichloro-4-((*E*)-(2,6-dichloro-4-(dimethylamino)phenyl)diazenyl)phenyl)acrylate (**29b**)

To a mixture of iodo **26b** (332 mg, 0.679 mmol), tri-*o*-tolylphosphine (21 mg, 0.068 mmol) and palladium(II) acetate (7,6 mg, 0.034 mmol) under argon were added degassed anhydrous DMF (2.7 mL), triethylamine (280 μL, 2.04 mmol), and *tert*-butyl acrylate (150 μL, 1.02 mmol), and the mixture was degassed with argon for 10 min and then heated to 100 °C overnight. The mixture was diluted with EtOAc, washed with 2× brine, dried over anhydrous Na_2_SO_4_, filtered, and concentrated under reduced pressure. The crude product was purified by flash column chromatography (5% to 10% EtOAc in hexane) to give **29b** (201 mg, 0.411 mmol, 60%) as a red solid: ^1^H NMR (400 MHz, CDCl_3_) δ 7.53 (s, 2H), 7.46 (d, *J* = 16.0 Hz, 1H), 6.73 (s, 2H), 6.39 (d, *J* = 15.9 Hz, 1H), 3.08 (s, 6H), 1.54 (s, 9H); ^13^C NMR (101 MHz, CDCl_3_) δ 165.6, 151.2, 149.2, 140.3, 135.5, 135.2, 132.6, 128.4, 127.8, 122.9, 112.6, 81.2, 40.4, 28.3; *m*/*z* (ESI+) 488.2 (MH^+^, 65%); HRMS (ESI+) [MH]^+^ calcd for C_21_H_22_Cl_4_N_3_O_2_^+^ 488.0455, found 488.0456.

#### (*E*)-*N*-(2-Aminophenyl)-3-(3,5-dichloro-4-((*E*)-(2,6-dichloro-4-(dimethylamino)phenyl)diazenyl)phenyl)acrylamide (**33b**)

A solution of *tert*-butoxy **29b** (120 mg, 0.245 mmol) in DCM (1.2 mL) and TFA (1.2 mL) was stirred at room temperature. After 1 h, the mixture was concentrated under reduced pressure to give a dark red solid.

To a solution of the crude acid mentioned above in DMF (1.2 mL) were added benzene-1,2-diamine (32 mg, 0.29 mmol), DIPEA (64 μL, 0.37 mmol), EDC (70 mg, 0.37 mmol), and HOBt (56 mg, 0.37 mmol). The mixture was stirred at room temperature overnight, diluted with EtOAc, washed with 3× brine, dried over anhydrous Na_2_SO_4_, filtered, and concentrated under reduced pressure. The crude product was purified by flash column chromatography (20% to 50% EtOAc in hexane) to give benzamide **33b** (108 mg, 0.206 mmol, 84%) as a dark orange solid: ^1^H NMR (400 MHz, DMSO-*d*_6_) δ 9.37 (s, 1H), 7.88 (s, 2H), 7.56 (d, *J* = 15.7 Hz, 1H), 7.39 (d, *J* = 7.8 Hz, 1H), 7.06 (d, *J* = 15.8 Hz, 1H), 6.97–6.91 (m, 1H), 6.91 (s, 2H), 6.76 (dd, *J* = 8.0, 1.4 Hz, 1H), 6.69–6.51 (m, 1H), 4.98 (s, 2H), 3.10 (s, 6H); ^13^C NMR (101 MHz, DMSO-*d*_6_) δ 162.9, 151.6, 147.7, 141.5, 136.2, 136.1, 133.3, 131.7, 128.2, 126.4, 125.9, 125.6, 124.6, 123.3, 116.3, 116.0, 112.2, 39.9; *m*/*z* (ESI+) 522.0 (MH^+^, 90%); HRMS (ESI+) [MH]^+^ calcd for C_23_H_20_Cl_4_N_5_O^+^ 522.0416, found 522.0427.

#### 4-Bromo-2,6-difluoroaniline^57^ (**S11**)

To a solution of 2,6-difluoroaniline (2.00 g, 15,5 mmol) in acetonitrile (31.0 mL) was added *N*-bromosuccinimide (2.76 g, 15.5 mmol) portionwise. The mixture was stirred at room temperature overnight, diluted with water, extracted with 8× hexane, dried over anhydrous Na_2_SO_4_, filtered, and concentrated under reduced pressure to give brominated product **S11** (1.90 g, 9.13 mmol, 59%) as a light brown solid: ^1^H NMR (400 MHz, CDCl_3_) δ 7.02–6.97 (m, 2H), 3.65 (br s, 2H); *m*/*z* (ESI+) 208.2 (MH^+^, 88%). NMR data were in agreement with the reported values.^57^

#### 4-Amino-3,5-difluorobenzonitrile^58^ (**S12**)

To a solution of bromo **S11** (1.68 g, 8.08 mmol) in anhydrous DMF (16.2 mL) was added copper(I) cyanide (2.17 g, 24.2 mmol). The mixture was stirred at 160 °C overnight, poured onto 30% aqueous ammonia, extracted with 3× EtOAc, dried over anhydrous Na_2_SO_4_, filtered, and concentrated under reduced pressure. The crude product was purified by flash column chromatography (2% to 15% EtOAc in hexane) to give nitrile **S12** (397 mg, 2.58 mmol, 32%) as a light purple solid: ^1^H NMR (400 MHz, CDCl_3_) δ 7.08 (dd, *J* = 6.0, 2.2 Hz, 1H), 4.20 (s, 1H); *m*/*z* (ESI+) 155.2 (MH^+^, 100%). NMR data were in agreement with the reported values.^58^

#### 4-Amino-3,5-difluorobenzoic Acid^58^ (**34a**)

A solution of nitrile **S12** (397 mg, 2.58 mmol) in aqueous sodium hydroxide (13.4 mL, 13.4 mmol) was heated to 110 °C overnight and washed with hexane, and the aqueous layer acidified with 1 M HCl, extracted with 3× EtOAc, dried over anhydrous Na_2_SO_4_, filtered, and concentrated under reduced pressure to give acid **34a** (444 mg, 2.56 mmol, quant) as a beige solid: ^1^H NMR (400 MHz, CDCl_3_) δ 7.64–7.53 (m, 2H). NMR data were in agreement with the reported values.^58^

#### Methyl (*E*)-4-((2,6-Difluoro-4-methoxyphenyl)diazenyl)-3,5-difluorobenzoate (**36a**)

A solution of sodium nitrite (128 mg, 1.85 mmol) in water (1.4 mL) was added dropwise to a suspension of acid **34a** (385 mg, 2.22 mmol) in water (8.0 mL) and concentrated HCl (370 μL) at 0 °C. The suspension was stirred at the same temperature for 1 h, and then a solution of 3,5-difluorophenol **23a** (241 mg, 1.85 mmol), sodium hydroxide (79 mg, 2.0 mmol), and potassium carbonate (410 mg, 2.97 mmol) in water (2.8 mL) was added dropwise, keeping the temperature below 0 °C. The mixture was then stirred at the same temperature for an additional 1 h, acidified with 1 M HCl, extracted with 3× EtOAc, dried over anhydrous Na_2_SO_4_, filtered, and concentrated under reduced pressure.

To a solution of the crude product mentioned above (581 mg, 1.85 mmol) in acetone (26.4 mL) were added potassium carbonate (2.56 g, 18.5 mmol) and methyl iodide (925 μL, 14.8 mmol). The mixture was stirred at 50 °C for 3 h, concentrated under reduced pressure, diluted with EtOAc, washed with 2× brine, dried over anhydrous Na_2_SO_4_, filtered, and concentrated under reduced pressure. The crude product was purified by flash column chromatography (5% to 10% EtOAc in hexane) to give **36a** (95 mg, 0.28 mmol, 15%) as a red solid: ^1^H NMR (400 MHz, CDCl_3_) δ 7.74–7.66 (m, 2H), 6.65–6.56 (m, 2H), 3.96 (s, 3H), 3.89 (s, 3H); ^13^C NMR (101 MHz, CDCl_3_) δ 164.6 (t, *J* = 3 Hz), 163.6 (t, *J* = 14 Hz), 157.9 (dd, *J* = 263, 7 Hz), 154.9 (dd, *J* = 256, 4 Hz), 135.2 (t, *J* = 11 Hz), 131.7 (t, *J* = 9 Hz), 126.2 (t, *J* = 9 Hz), 114.2–113.7 (m), 99.2 (dd, *J* = 24, 3 Hz), 56.4, 53.0; *m*/*z* (ESI+) 343.2 (MH^+^, 100%).

#### (*E*)-4-((2,6-Difluoro-4-methoxyphenyl)diazenyl)-3,5-difluorobenzoic Acid (**37a**)

To a solution of methyl ester **36a** (95 mg, 0.28 mmol) in THF (6.2 mL) and MeOH (3.1 mL) was added an aqueous solution of sodium hydroxide (0.8 M, 4.3 mL, 3.5 mmol). The mixture was stirred at room temperature overnight, concentrated, acidified with 1 M HCl, extracted with 3× EtOAc, dried over anhydrous Na_2_SO_4_, filtered, and concentrated under reduced pressure to give acid **37a** (95 mg, 0.29 mmol, quant) as a red solid. The product was taken to the following step without further purification: ^1^H NMR (400 MHz, methanol-*d*_4_) δ 7.78–7.73 (m, 2H), 6.90–6.76 (m, 2H), 3.95 (s, 3H); *m*/*z* (ESI+) 329.2 (MH^+^, 100%).

#### (*E*)-*N*-(2-Aminophenyl)-4-((2,6-difluoro-4-methoxyphenyl)diazenyl)-3,5-difluorobenzamide (**38a**)

To a solution of acid **37a** (95 mg, 0.29 mmol) in DMF (1.4 mL) were added benzene-1,2-diamine (38 mg, 0.35 mmol), DIPEA (76 μL, 0.43 mmol), EDC (83 mg, 0.43 mmol), and HOBt (66 mg, 0.43 mmol). The mixture was stirred at room temperature overnight, diluted with EtOAc, washed with 3× brine, dried over anhydrous Na_2_SO_4_, filtered, and concentrated under reduced pressure. The crude product was purified by flash column chromatography (EtOAc in hexane) to give benzamide **38a** (50 mg, 0.12 mmol, 41%) as an orange solid. NMR data were collected after heating a DMSO-*d*_6_ solution of the product at 80 °C for 2 h to obtain the *trans* form (15% of *cis* remained): ^1^H NMR (400 MHz, DMSO-*d*_6_) 1:1.6 mixture of isomers, major isomer reported δ 9.88 (s, 1H), 7.92 (d, *J* = 9.8 Hz, 2H), 7.16 (dd, *J* = 7.8, 1.5 Hz, 1H), 7.11–7.03 (m, 2H), 7.02–6.97 (m, 1H), 6.78 (dd, *J* = 8.0, 1.4 Hz, 1H), 6.60 (td, *J* = 7.5, 1.4 Hz, 1H), 5.03 (s, 2H), 3.92 (s, 3H); ^13^C NMR (101 MHz, DMSO-*d*_6_) δ 163.7, 162.3, 156.9 (dd, *J* = 260, 7 Hz), 154.0 (dd, *J* = 257, 4 Hz), 143.6, 137.1 (t, *J* = 8 Hz), 132.6 (t, *J* = 6 Hz), 127.1, 127.1, 125.1–124.7 (m), 122.1, 116.0, 115.9, 112.6 (d, *J* = 23 Hz), 99.8 (dd, *J* = 24, 3 Hz), 56.9; *m*/*z* (ESI+) 419.2 (MH^+^, 55%); HRMS (ESI+) [MH]^+^ calcd for C_20_H_15_F_4_N_4_O_2_^+^ 419.1126, found 419.1127.

#### Methyl (*E*)-3,5-Dichloro-4-((2,6-dichloro-4-methoxyphenyl)diazenyl)benzoate (**36b**)

A solution of sodium nitrite (106 mg, 1.53 mmol) in water (1.4 mL) was added dropwise to a suspension of 4-amino-3,5-dichlorobenzoic acid **34b** (537 mg, 2.61 mmol) in water (8 mL) and concentrated HCl (300 μL) at 0 °C. The suspension was stirred at the same temperature for 1 h, and then a solution of 3,5-dichlorophenol **23b** (250 mg, 1.53 mmol), sodium hydroxide (66 mg, 1.6 mmol), and potassium carbonate (339 mg, 2.45 mmol) in water (2.8 mL) was added dropwise, keeping the temperature below 0 °C. The mixture was then stirred at the same temperature for an additional 1 h, acidified with 1 M HCl, extracted with 3× EtOAc, dried over anhydrous Na_2_SO_4_, filtered, and concentrated under reduced pressure. The crude product was purified by flash column chromatography (10% to 70% EtOAc in hexane) to give a 1:4 mixture of the product and starting material.

To a solution of the mixture mentioned above (76 mg) in acetone (2.9 mL) were added potassium carbonate (276 mg, 2.00 mmol) and methyl iodide (100 μL, 1.60 mmol). The mixture was stirred at 50 °C for 3 h, concentrated under reduced pressure, diluted with EtOAc, washed with 2× brine, dried over anhydrous Na_2_SO_4_, filtered, and concentrated under reduced pressure. The crude product was purified by flash column chromatography (EtOAc in hexane) to give **36b** (77 mg, 0.19 mmol, 94%) as a red solid: ^1^H NMR (400 MHz, CDCl_3_) δ 8.09 (s, 2H), 7.03 (s, 2H), 3.97 (s, 3H), 3.89 (s, 3H); ^13^C NMR (101 MHz, CDCl_3_) δ 164.6, 160.7, 151.6, 140.3, 130.9, 130.7, 130.4, 126.9, 115.7, 56.3, 53.0; *m*/*z* (ESI+) 406.9 (MH^+^, 45%).

#### (*E*)-3,5-Dichloro-4-((2,6-dichloro-4-methoxyphenyl)diazenyl)benzoic Acid (**37b**)

To a solution of methyl ester **36b** (77 mg, 0.19 mmol) in THF (4.2 mL) and MeOH (2.1 mL) was added an aqueous solution of sodium hydroxide (2.9 mL, 2.4 mmol). The mixture was stirred at room temperature overnight, concentrated, acidified with 1 M HCl, and filtered to give acid **37b** (30 mg, 0.076 mmol, 40%) as a red solid: ^1^H NMR (400 MHz, methanol-*d*_4_) δ 8.10 (s, 2H), 7.20 (s, 2H), 3.94 (s, 3H); *m*/*z* (ESI+) 393.0 (MH^+^, 70%).

#### (*E*)-*N*-(2-Aminophenyl)-3,5-dichloro-4-((2,6-dichloro-4-methoxyphenyl)diazenyl)benzamide (**38b**)

To a solution of acid **37b** (95 mg, 0.24 mmol) in DMF (1.2 mL) were added benzene-1,2-diamine (31 mg, 0.29 mmol), DIPEA (63 μL, 0.36 mmol), EDC (69 mg, 0.36 mmol), and HOBt (55 mg, 0.36 mmol). The mixture was stirred at room temperature overnight, diluted with EtOAc, washed with 3× brine, dried over anhydrous Na_2_SO_4_, filtered, and concentrated under reduced pressure. The crude product was purified by flash column chromatography (EtOAc in hexane) to give benzamide **38b** (44 mg, 0.091 mmol, 38%) as an orange solid: ^1^H NMR (400 MHz, DMSO-*d*_6_) δ 10.05 (s, 1H), 8.25 (s, 2H), 7.36 (s, 2H), 7.22 (d, *J* = 7.6 Hz, 1H), 7.07 (t, *J* = 7.3 Hz, 1H), 6.89 (d, *J* = 7.9 Hz, 1H), 6.73 (t, *J* = 7.6 Hz, 1H), 3.93 (s, 3H); ^13^C NMR (101 MHz, DMSO-*d*_6_) δ 162.4, 160.9, 149.0, 141.3, 139.1, 135.9, 129.5, 129.0, 127.2, 127.1, 125.4, 123.5, 117.9, 117.1, 116.0, 56.7; *m*/*z* (ESI+) 483.1 (MH+, 70%); HRMS (*m*/*z*) [MH]^+^ calcd for C_20_H_15_Cl_4_N_4_O_2_^+^ 482.9944, found 482.9887.

#### (*E*)-3-(4-((*E*)-(2,6-Difluoro-4-methoxyphenyl)diazenyl)-3,5-difluorophenyl)-*N*-hydroxyacrylamide (**39**)

To a solution of acid **30a** (50 mg, 0.11 mmol) in DMF (550 μL) were added *O*-(tetrahydro-2*H*-pyran-2-yl)hydroxylamine (15 mg, 0.13 mmol), EDC (32 mg, 0.16 mmol), HOBt (25 mg, 0.16 mmol), and DIPEA (30 μL, 0.16 mmol). The mixture was stirred at room temperature overnight, diluted with EtOAc, washed with 2× brine, dried over anhydrous Na_2_SO_4_, filtered, and concentrated under reduced pressure to give an orange solid.

A solution of the crude protected hydroxamide mentioned above (50 mg, 0.11 mmol) in DCM (740 μL) and TFA (370 μL) was stirred at room temperature for 2 h and then concentrated under reduced pressure. The crude product was purified by C18 column chromatography to give hydroxamide **39** (10 mg, 0.027 mmol, 25%) as an orange solid: ^1^H NMR (400 MHz, DMSO-*d*_6_) δ 10.90 (s, 1H), 9.18 (s, 1H), 7.57 (d, *J* = 10.9 Hz, 2H), 7.49 (d, *J* = 16.2 Hz, 1H), 7.02 (d, *J* = 11.9 Hz, 2H), 6.65 (d, *J* = 15.6 Hz, 1H), 3.91 (s, 3H); ^13^C NMR (101 MHz, DMSO-*d*_6_) δ 163.5–163.1 (m), 161.9, 156.7 (dd, *J* = 260, 7 Hz), 154.8 (dd, *J* = 258, 6 Hz), 138.8, 135.5, 130.8, 125.1 (d, *J* = 9 Hz), 123.4, 111.8 (d, *J* = 21 Hz), 99.7 (d, *J* = 23 Hz), 56.8; *m*/*z* (ESI+) 370.2 (MH^+^, 100%); HRMS (ESI-) [M – H]^−^ calcd for C_16_H_10_F_4_N_3_O_3_^–^ 368.0664, found 368.0641.

### Photochemistry

Ultraviolet–visible (UV–vis) spectra were recorded using a Tecan Spark 20M Multimode Microplate reader. Samples (200 μL of compound solution/well) were prepared at 25–100 μM in DMSO. Samples were measured between 300 and 800 nm with 2 nm fixed intervals in 96-well transparent plates. Illumination at different wavelengths was performed for 2 min using 96-well LED array plates (LEDA Teleopto) placed below the samples. Illumination was done at the highest potency for each plate, which corresponded to 7.0 mW/cm^2^ for 365 nm, 11 mW/cm^2^ for 380 nm, 19 mW/cm^2^ for 405 nm, 13 mW/cm^2^ for 420 nm, 14 mW/cm^2^ for 455 nm, 14 mW/cm^2^ for 470 nm, 19 mW/cm^2^ for 500 nm, and 13 mW/cm^2^ for 550 nm. Potencies were measured using a Thorlabs PM100D power energy meter connected with a standard photodiode power sensor (S120VC).

The *E*/*Z* composition under different temperature and light conditions was determined by HPLC and quantified by integration at 254 nm.

The spectrum of the pure *Z* isomer was estimated by subtracting the spectrum of the pure *E* isomer from the spectrum of the PSS upon illumination at a wavelength at which the proportion of each isomer had been previously determined by HPLC, using the equation
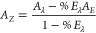
where *A*_λ_ is the absorbance in the PSS, *A*_*E*_ is the absorbance in the dark, and %*E* is the fraction of the *E* isomer at the PSS.

In the cases in which there was a mixture of *E* and *Z* isomers in the dark, the spectrum of each isomer was determined by measuring the absorbance of the PSS upon illumination at two different wavelengths where the proportion of each isomer had been previously determined by HPLC, using the equation
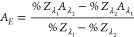


To determine the thermal relaxation rates of the *cis* isomers at room temperature, a 25–100 μM solution of the compounds in DMSO was illuminated for 2 min, and then the absorbance was measured at the wavelength with the largest difference in absorbance between both isomers, at fixed intervals in the dark. Thermal half-lives were calculated by plotting the absorbance readings versus time and fitting the obtained curve to an exponential decay function, with GraphPad Prism 6. For the rates at 37 °C, the 10 mM stock in DMSO was illuminated for 2 min and then diluted to a 10 μM solution in DMEM with 0.1% DMSO. The solution was kept in an incubator at 37 °C, and samples were taken at fixed intervals and analyzed by HPLC.

### HDAC1 Assays

#### Assay Reagents

Recombinant HDAC1 (BPS Bioscience, snap frozen in 2 μg aliquots upon receipt, diluted to 200 ng/mL with HDAC buffer), HDAC buffer (25 mM Tris-HCl, 137 mM NaCl, 2.7 mM KCl, 1 mM MgCl_2_, set to pH 8 with concentrated HCl; BSA at 0.1 mg/mL added before the assay), substrate (Boc-Lys(Ac)-AMC, Enzo, 10 mM stock, diluted to 30 μM with HDAC buffer), stop solution (6 mg/mL trypsin and 300 μM SAHA (from 40 mM DMSO stock) in HDAC buffer).

Stock solutions of the compounds were diluted to 1 mM in DMSO and then heated to 60 or 80 °C to obtain solutions with the maximum amount of the *trans* isomer (dark). For the “light” solutions, the diluted solutions were illuminated with 96-well LED array plates for 3 min at the maximum potency. The 1 mM solutions were then serially diluted with HDAC buffer to 10 times the desired concentration, maintaining DMSO at 10%.

To initiate the enzyme reaction, reagents were added to 96-well low-binding black flat plates with a clear bottom in this order: compounds (10 μL, 10 μM to 3 nM), enzyme (40 μL, 8 ng/well), and substrate (50 μL, 15 μM). In control wells without the compound, inhibitor, or enzyme, HDAC buffer was added instead. Plates were kept at room temperature for 1 h either in the dark or under illumination with 96-well LED array plates at preoptimized potencies (2 mW/cm^2^ for 365 and 380 nm, 7 mW/cm^2^ for 420 nm, and 6 mW/cm^2^ for 550 nm). Then, 50 μL of the stop solution was added and the plates were incubated at 37 °C for 20 min in the dark. Fluorescence was measured at a λ_em_ of 460 nm and a λ_ex_ of 380 nm. Enzyme activity is expressed as a percentage with respect to the nontreated cells, after subtracting the blank (well with no enzyme and no compound).

### Cell Assays

HeLa, MCF7, and HT29 cells were maintained in adherent cultures in DMEM supplemented with 10% FBS. KG1 cells were maintained in a suspension culture in IMDM supplemented with 20% FBS.

Stock solutions of the compounds were heated to 60 or 80 °C to obtain solutions with the maximum amount of the *trans* isomer (dark). For the “light” solutions, the stock solutions were illuminated with 96-well LED array plates for 3 min at the maximum potency. The solutions were then serially diluted with DMEM to give 20 times the desired final concentration, maintaining DMSO at 10%.

### Whole-Cell Inhibition Assay

See the HDAC1 Assays for details about the reagents, buffers, and assay solutions.

HeLa cells were seeded in transparent flat bottom plates at a density of 15 × 10^3^ cells/well in a volume of 45 μL. After 24 h, 2.5 μL of the compound solution and 2.5 μL of the HDAC substrate solution in DMEM were added to final concentrations of 50 and 100 μM, respectively. The plates were incubated at 37 °C for 3 h, and 50 μL of the stop solution was added. The plates were incubated for an additional 1 h at 37 °C. The fluorescence was measured at a λ_em_ of 460 nm and a λ_ex_ of 380 nm. Enzyme activity is expressed as a percentage with respect to the nontreated cells, after subtracting the blank (nontreated cells with no substrate).

### Cell Viability Assay

Cells were seeded in 96-well black flat bottom plates with a clear bottom at a density of 5 × 10^3^ cells/well (HeLa) or 1 × 10^4^ cells/well (MCF7, HT29, and KG1), in a volume of 95 μL. After 24 h, 5 μL of compound solutions (20×) were added. Cells were incubated for 48 h.

Viability of HeLa, MCF7, and HT20 cells was determined with a CellTiter 96 assay (Promega), according to the manufacturer’s instructions. Briefly, 20 μL of CellTiter 96 reagent was added to each well, the plates were incubated at 37 °C for 2 h, and the absorbance at 490 nm was recorded. Viability is expressed as a percentage with respect to the nontreated cells.

Viability of KG1 cells was determined with a CellTiter-Glo (Promega) assay, according to the manufacturer’s instructions. Briefly, 100 μL of CellTiter-Glo reagent was added to each well, the plates were kept at room temperature for 10 min, and the luminescence was recorded. Viability is expressed as a percentage with respect to the nontreated cells.

## Supporting Information Available

^1^H and ^13^C NMR spectra, HPLC traces, and full photochemical characterization of the final compounds (PDF)
Molecular formula strings (CSV)

## Abbreviations

HDAC: histone deacetylase
HA: hydroxamic acid
LED: light-emitting diode
OAA: *o*-aminoanilide
PSS: photostationary state
SAR: structure–activity relationship
THP: tetrahydropyran
TLC: thin layer chromatography
ZBD: zinc binding domain
