# Supplementary material for: Visible-Light-Controlled Histone Deacetylase Inhibitors for Targeted Cancer Therapy
Source: J Med Chem. 2023 Jan 19;66(3):1909–27. doi: 10.1021/acs.jmedchem.2c01713 (PMC9949698; doi:10.1021/acs.jmedchem.2c01713)
Supplement: Supplementary file 1 — jm2c01713_si_001.pdf [file jm2c01713_si_001.pdf]

SUPPORTING INFORMATION

Visible-light-controlled histone deacetylase inhibitors for targeted cancer therapy

Laia Josa-Culleré* and Amadeu Llebaria*

MCS, Laboratory of Medicinal Chemistry & Synthesis, Department of Biological Chemistry, Institute for Advanced Chemistry of Catalonia (IQAC-CSIC), Jordi Girona 18-26, 08034 Barcelona, Spain

*Corresponding authors

Email addresses: laia.josacullere@iqac.csic.es; amadeu.llebaria@iqac.csic.es

Table of contents

Figure S1. Photochemical properties of **1**. 3

Figure S2. Photochemical properties of **2**. 4

Figure S3. UV-Vis spectra of **12**. 4

Figure S4. Photochemical properties of **17**. 5

Figure S5. Photochemical properties of **18**. 6

Figure S6. UV-Vis spectra of **19**. 6

Figure S7. Photochemical properties of **20**. 7

Figure S8. Photochemical properties of **21**. 8

Figure S9. Photochemical properties of **32a**. 9

Figure S10. Photochemical properties of **32b**. 10

Figure S11. Photochemical properties of **32c**. 11

Figure S12. Photochemical properties of **32d**. 12

Figure S13. Photochemical properties of **32e**. 13

Figure S14. Photochemical properties of **33a**. 14

Figure S15. Photochemical properties of **33b**. 15

Figure S16. Photochemical properties of **38a**. 16

Figure S17. Photochemical properties of **38b**. 17

Figure S18. Photochemical properties of **39**. 18

Figure S19. UV-Vis spectra of **11** after illumination with 365nm at different times and intensities. 19

Figure S20. Activity of non-treated recombinant HDAC1 under either dark or illumination conditions. 19

Figure S21. Inhibition of HDAC1 by SAHA under either dark or illumination conditions at different wavelengths. 20

Figure S22. Activity of representative compounds in a whole-cell HDAC inhibition assay at 50 µM under either dark or pre-illumination (380 or 550 nm) conditions. 20

Figure S23. Effect of 50 µM of **11**, **32a** and **38a** on HeLa, HT29, MCF7 and KG1 cells under either dark or pre-illumination conditions. 21

Scheme S1. Synthesis of compounds **19**, **20**, **21** and **39**. 22

NMR and HPLC spectra of final compounds. 23

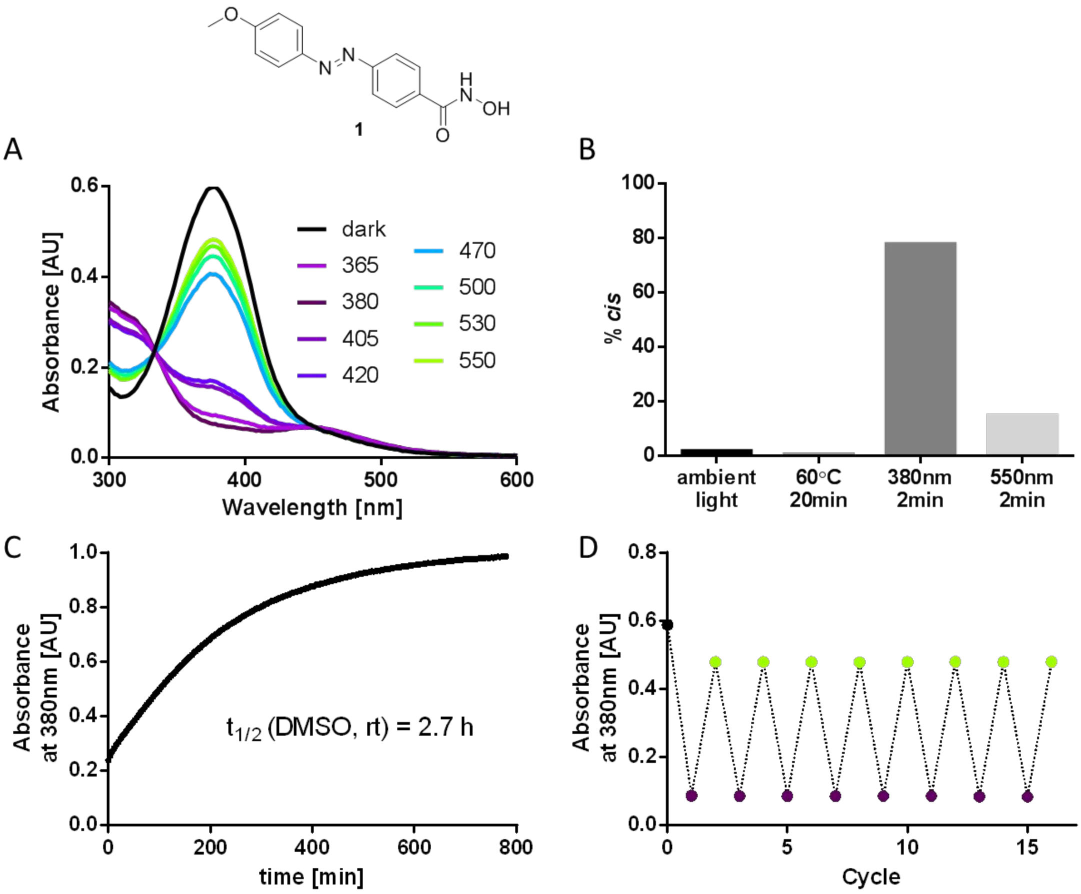

Figure S1. Photochemical properties of **1** in DMSO. (A) UV-Vis spectra of 1 (25 µM) under different light conditions. (B) Quantification of E/Z composition of **1** (100 µM) by HPLC after different temperature and light conditions, measured at the isosbestic point (331 nm). (C) Half-life estimation of cis-**1** (50 µM) after irradiation (380 nm) following its absorbance at 380 nm. (D) Absorbance at 380 nm of **1** (25 µM) after multiple illumination cycles (380 – 550 nm, 2 min each). In all cases, illumination at 380 nm was done at 11 mW/cm^2, and 550 nm at 12 mW/cm^2.

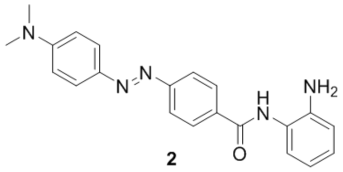

2

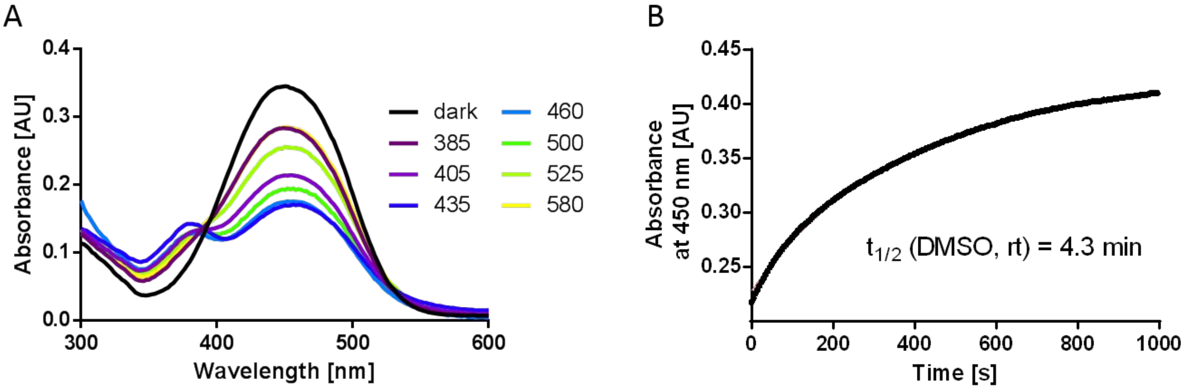

Figure S2. Photochemical properties of **2** in DMSO at 25 µM. (A) UV-Vis spectra of **2** under different light conditions. (B) Half-life estimation of *cis* after irradiation (460 nm) following its absorbance at 450 nm. Illumination performed with a coolLed.

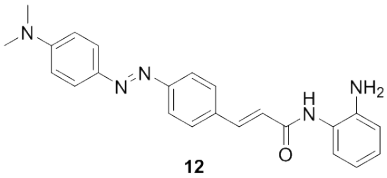

12

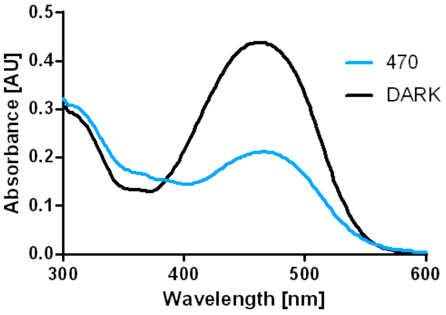

Figure S3. UV-Vis spectra of **12** (25 µM) in DMSO.

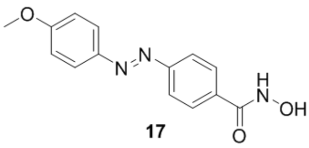

17

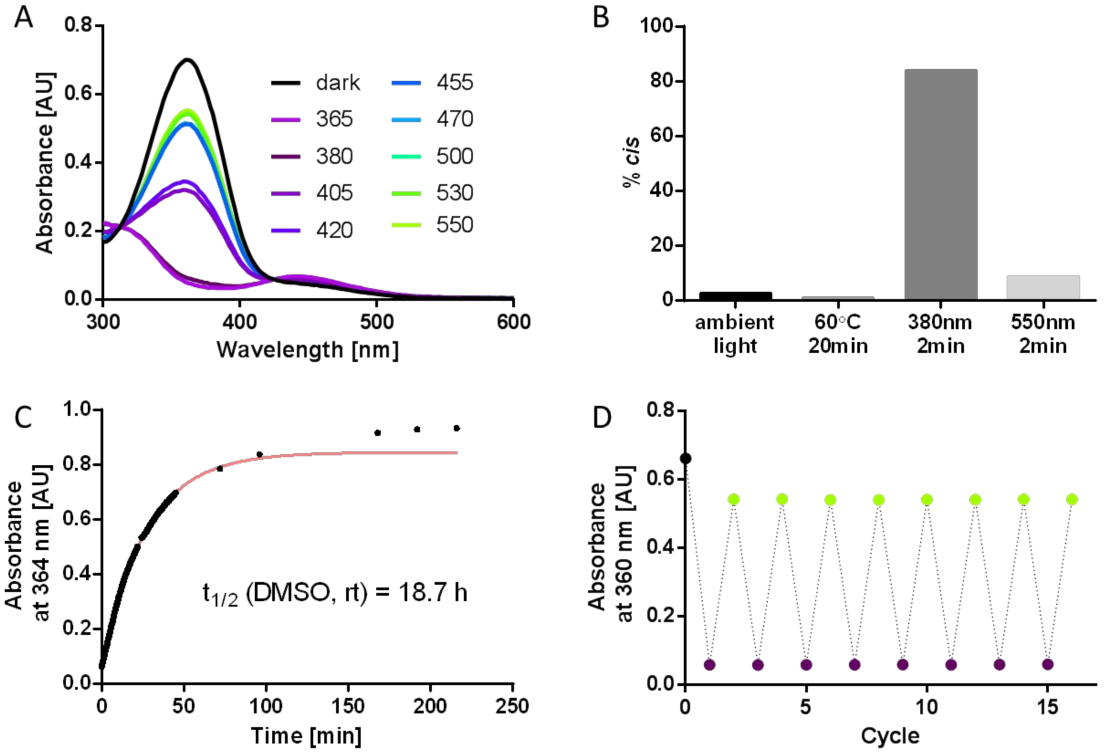

Figure S4. Photochemical properties of **17** in DMSO. (A) UV-Vis spectra of **17** (50 µM) under different light conditions. (B) Quantification of E/Z composition of **17** (100 µM) after different temperature and light conditions, measured by HPLC at the isosbestic point (332 nm). (C) Half-life estimation of *cis*-**17** (50 µM) after irradiation (380 nm) following its absorbance at 364 nm. (D) Absorbance at 360 nm of **17** (50 µM) after multiple illumination cycles (380 – 550 nm, 2 min each). In all cases, illumination at 380 nm was done at 11 mW/cm^2, and 550 nm at 12 mW/cm^2.

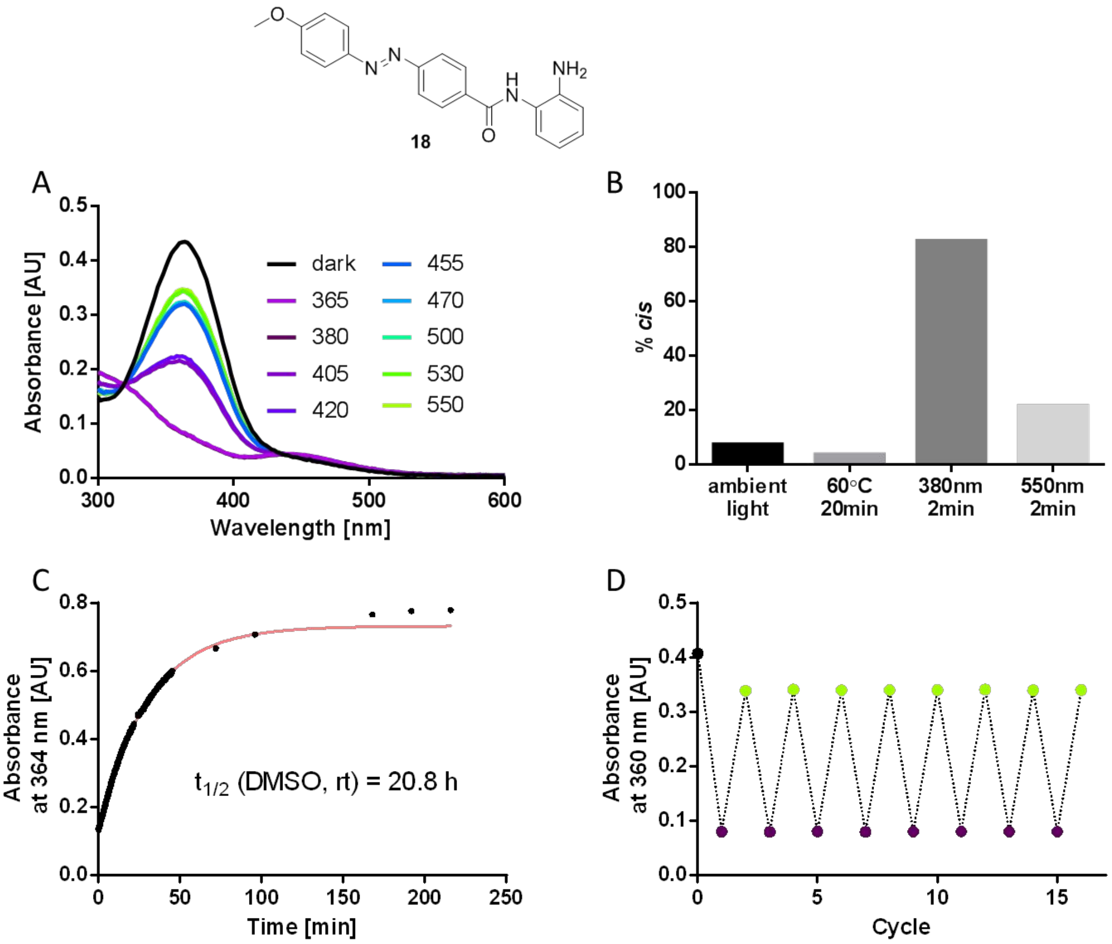

Figure S5. Photochemical properties of **18** in DMSO. (A) UV-Vis spectra of **18** (25 µM) under different light conditions. (B) Quantification of E/Z composition of **18** (100 µM) after different temperature and light conditions, measured by HPLC at the isosbestic point (316 nm). (C) Half-life estimation of *cis*-**18** (25 µM) after irradiation (380 nm) following its absorbance at 364 nm. (D) Absorbance at 360 nm of **18** (25 µM) after multiple illumination cycles (380 – 550 nm, 2 min each). In all cases, illumination at 380 nm was done at 11 mW/cm^2, and 550 nm at 12 mW/cm^2.

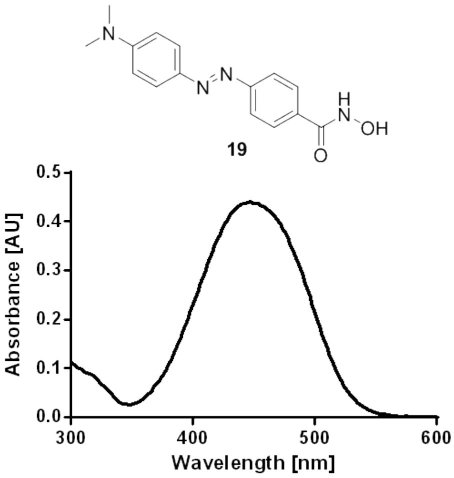

Figure S6. UV-Vis spectra of **19** (25 µM) in DMSO.

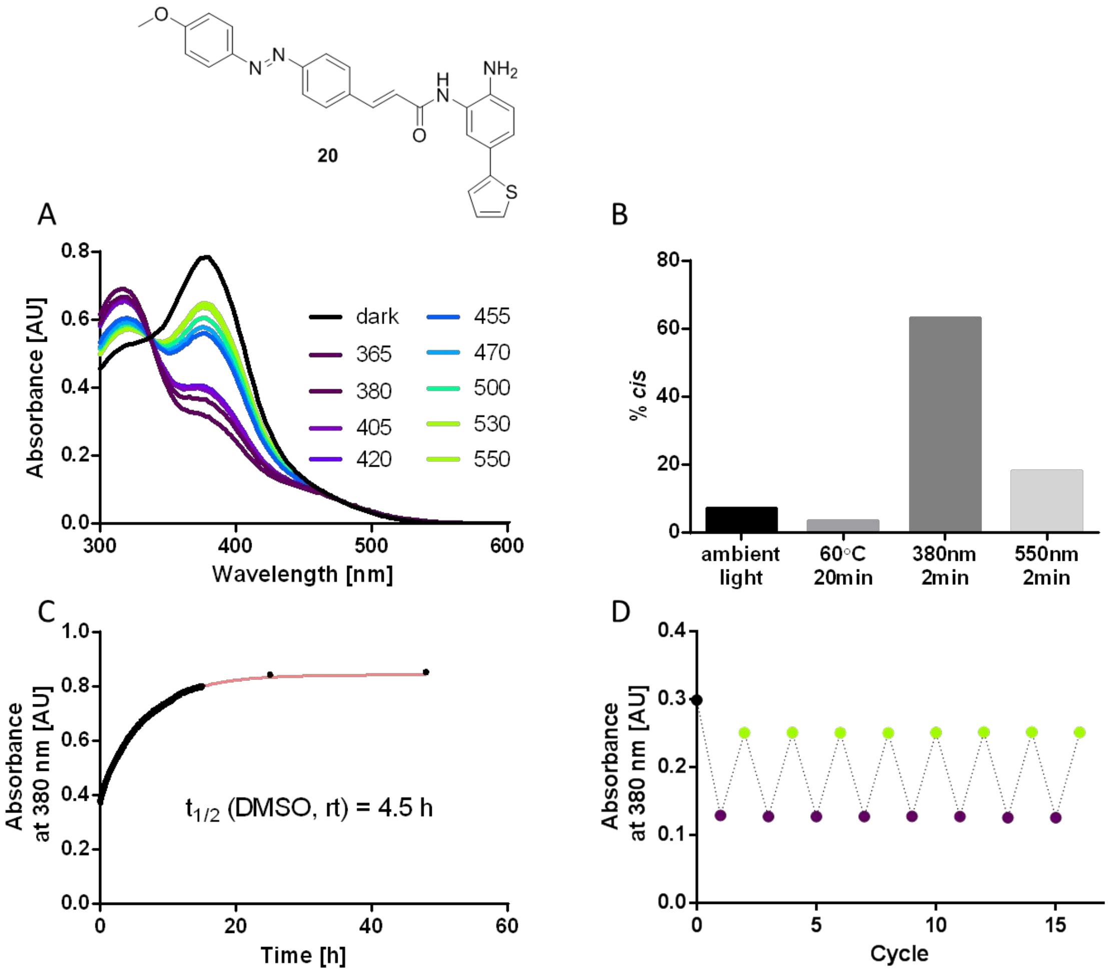

Figure S7. Photochemical properties of **20** in DMSO. (A) UV-Vis spectra of **20** (50 µM) under different light conditions. (B) Quantification of E/Z composition of **20** (100 µM) after different temperature and light conditions, measured by HPLC at the isosbestic point (338 nm). (C) Half-life estimation of *cis*-**20** (50 µM) after irradiation (380 nm) following its absorbance at 380 nm. (D) Absorbance at 380 nm of **20** (25 µM) after multiple illumination cycles (380 – 550 nm, 2 min each). In all cases, illumination at 380 nm was done at 11 mW/cm^2, and 550 nm at 12 mW/cm^2.

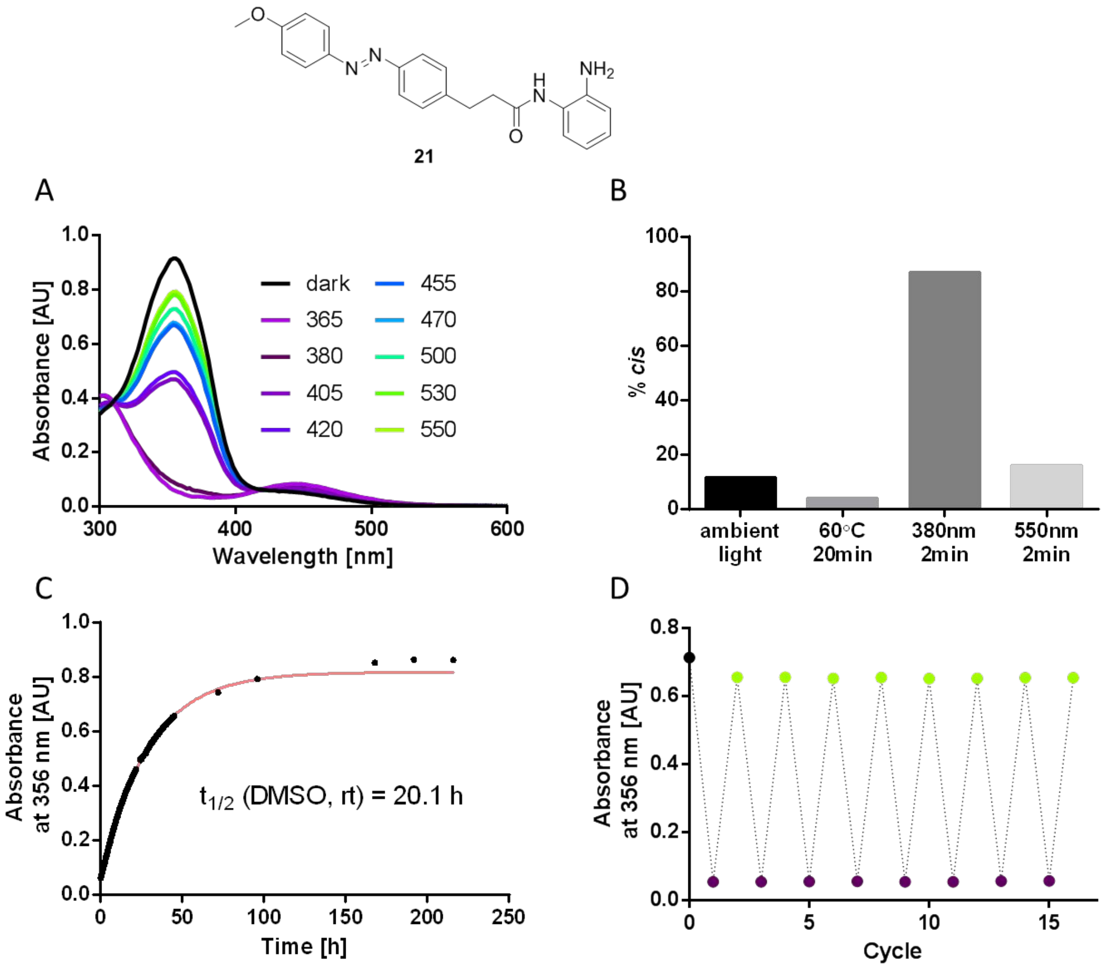

Figure S8. Photochemical properties of **21** in DMSO. (A) UV-Vis spectra of **21** (50 µM) under different light conditions. (B) Quantification of E/Z composition of **21** (100 µM) after different temperature and light conditions, measured by HPLC at the isosbestic point (310 nm). (C) Half-life estimation of *cis*-**21** (50 µM) after irradiation (380 nm) following its absorbance at 356 nm. (D) Absorbance at 356 nm of **21** (50 µM) after multiple illumination cycles (380 – 550 nm, 2 min each). In all cases, illumination at 380 nm was done at 11 mW/cm^2, and 550 nm at 12 mW/cm^2.

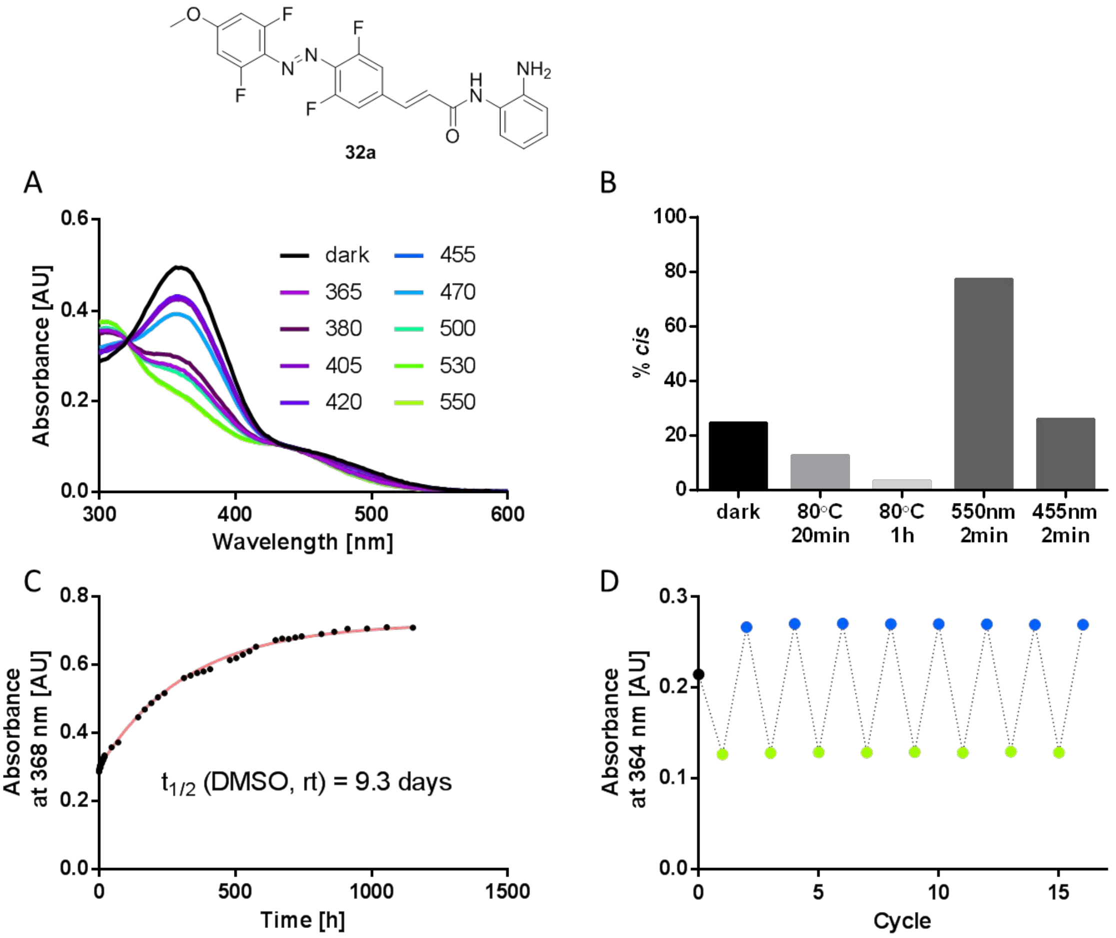

Figure S9. Photochemical properties of **32a** in DMSO. (A) UV-Vis spectra of **32a** (25 µM) under different light conditions. (B) Quantification of E/Z composition of **32a** (100 µM) after different temperature and light conditions, measured by HPLC at the isosbestic point (320 nm). (C) Half-life estimation of *cis*-**32a** (50 µM) after irradiation (550 nm) following its absorbance at 368 nm. (D) Absorbance at 364 nm of **32a** (25 µM) after multiple illumination cycles (550 – 455 nm, 2 min each). In all cases, illumination at 455 nm was done at 9 mW/cm^2, and 550 nm at 12 mW/cm^2.

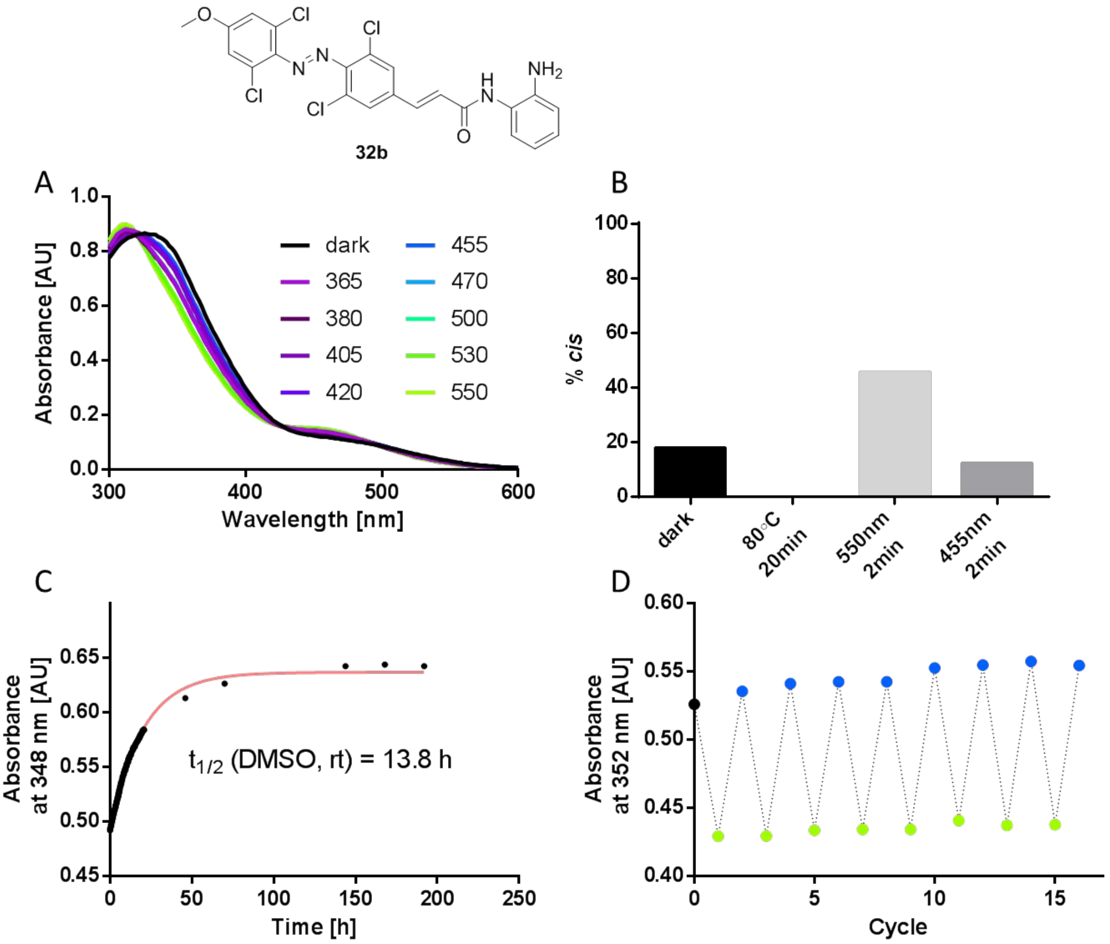

Figure S10. Photochemical properties of **32b** in DMSO. (A) UV-Vis spectra of **32b** (50 µM) under different light conditions. (B) Quantification of E/Z composition of **32b** (100 µM) after different temperature and light conditions, measured by HPLC at the isosbestic point (316 nm). (C) Half-life estimation of *cis*-**32b** (50 µM) after irradiation (550 nm) following its absorbance at 364 nm. (D) Absorbance at 380 nm of **32b** (50 µM) after multiple illumination cycles (550 – 455 nm, 2 min each). In all cases, illumination at 455 nm was done at 9 mW/cm², and 550 nm at 12 mW/cm².

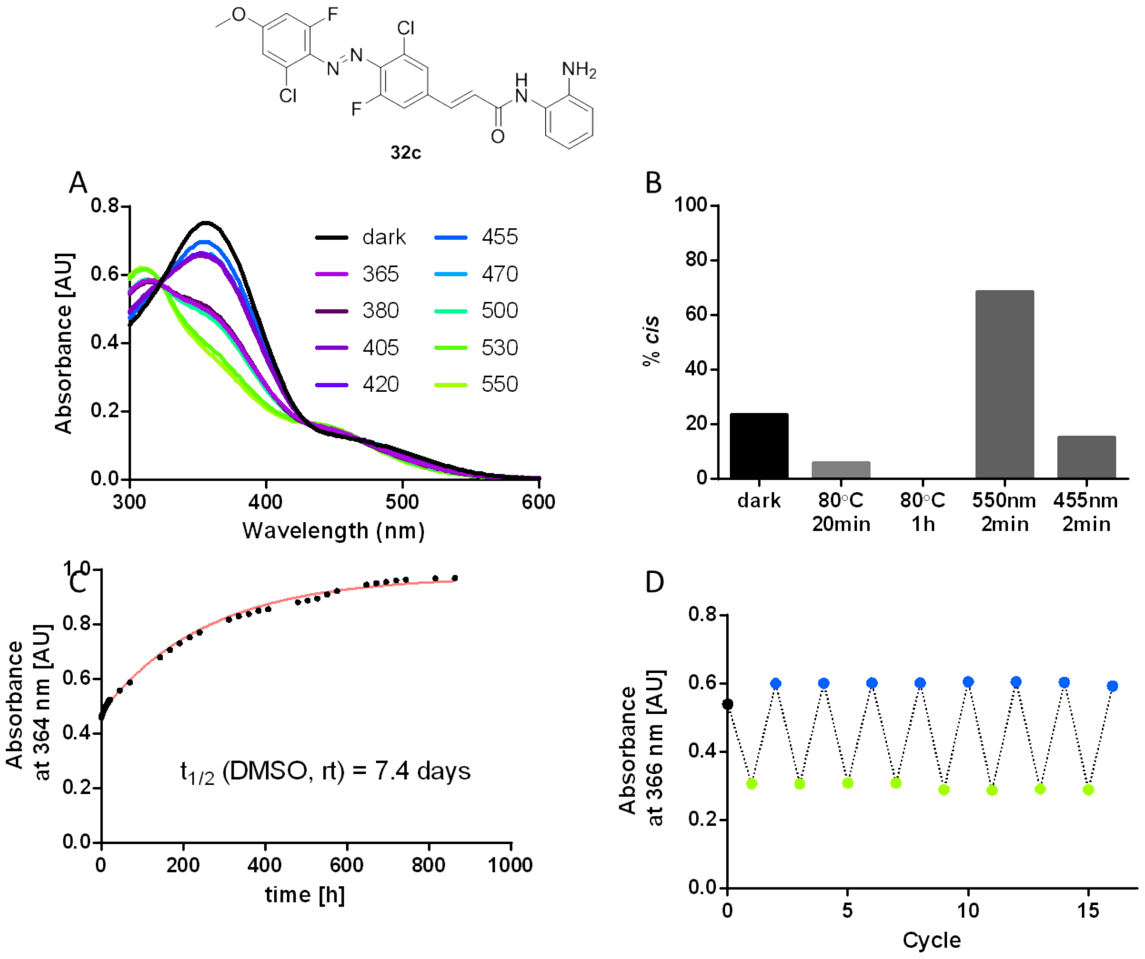

Figure S11. Photochemical properties of **32c** in DMSO. (A) UV-Vis spectra of **32c** (50 µM) under different light conditions. (B) Quantification of E/Z composition of **32c** (100 µM) after different temperature and light conditions, measured by HPLC at the isosbestic point (323 nm). (C) Half-life estimation of *cis*-**32c** (50 µM) after irradiation (550 nm) following its absorbance at 348 nm. (D) Absorbance at 366 nm of **32c** (50 µM) after multiple illumination cycles (550 – 455 nm, 2 min each). In all cases, illumination at 455 nm was done at 9 mW/cm^2, and 550 nm at 12 mW/cm^2.

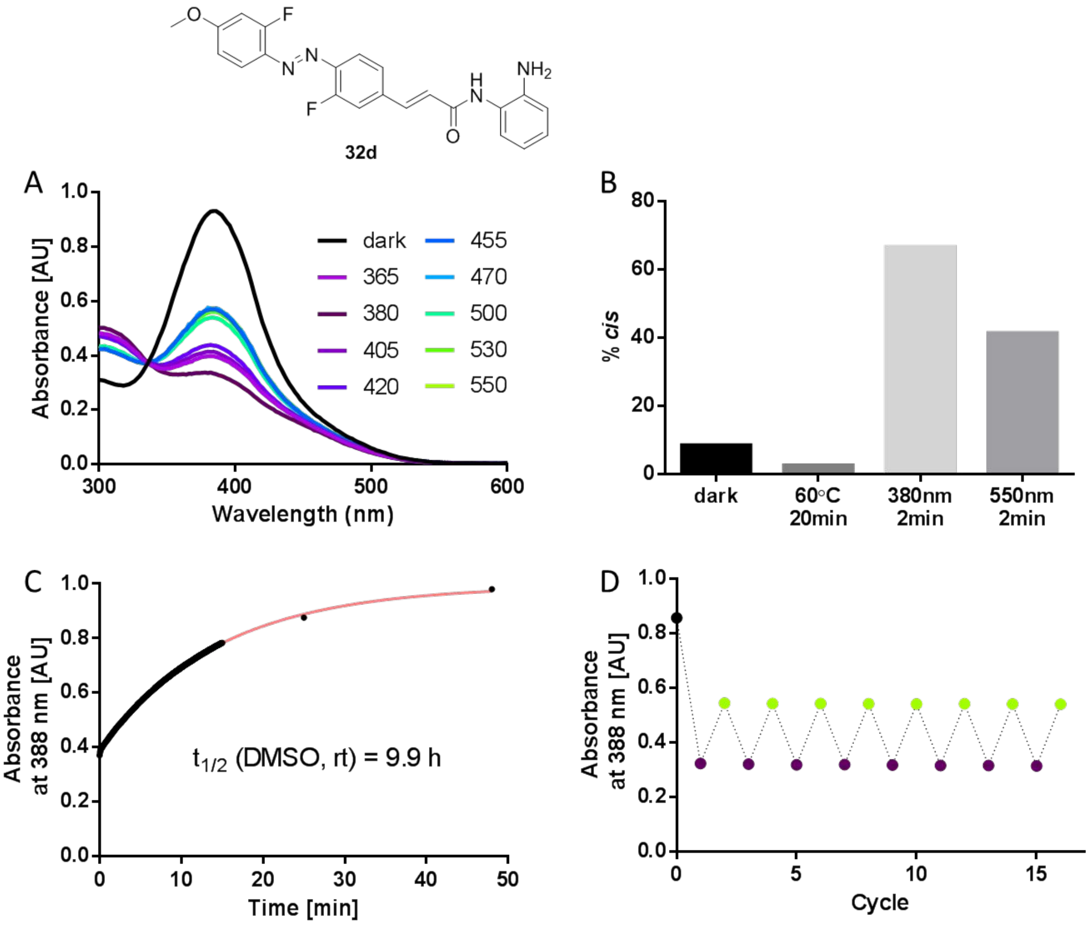

Figure S12. Photochemical properties of **32d** in DMSO. (A) UV-Vis spectra of **32d** (50 µM) under different light conditions. (B) Quantification of E/Z composition of **32d** (100 µM) after different temperature and light conditions, measured by HPLC at the isosbestic point (336 nm). (C) Half-life estimation of *cis*-**32d** (50 µM) after irradiation (380 nm) following its absorbance at 388 nm. (D) Absorbance at 388 nm of **32d** (50 µM) after multiple illumination cycles (380 – 550 nm, 2 min each). In all cases, illumination at 380 nm was done at 11 mW/cm^2, and 550 nm at 12 mW/cm^2.

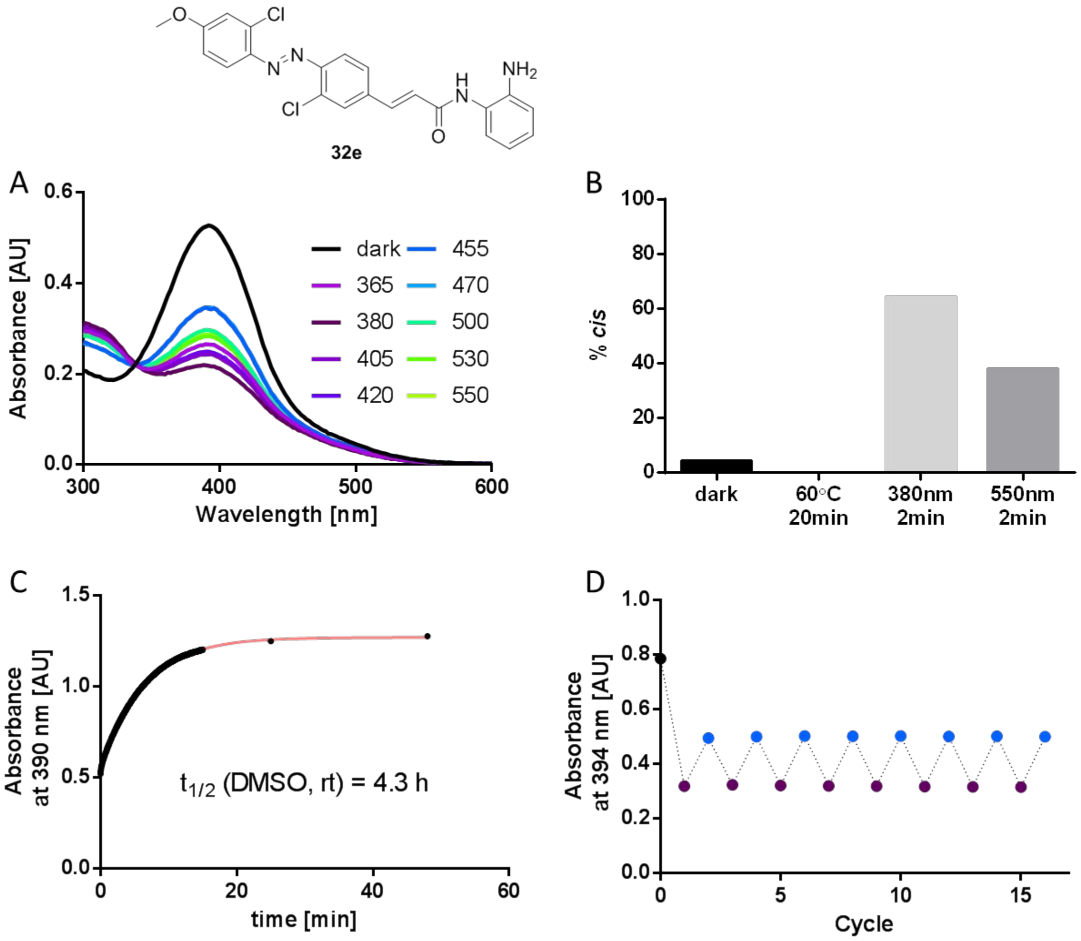

Figure S13. Photochemical properties of **32e** in DMSO. (A) UV-Vis spectra of **32e** (25 µM) under different light conditions. (B) Quantification of E/Z composition of **32e** (100 µM) after different temperature and light conditions, measured by HPLC at the isosbestic point (333 nm). (C) Half-life estimation of *cis*-**32e** (50 µM) after irradiation (380 nm) following its absorbance at 390 nm. (D) Absorbance at 394 nm of **32e** (25 µM) after multiple illumination cycles (380 – 550 nm, 2 min each). In all cases, illumination at 380 nm was done at 11 mW/cm^2, and 550 nm at 12 mW/cm^2.

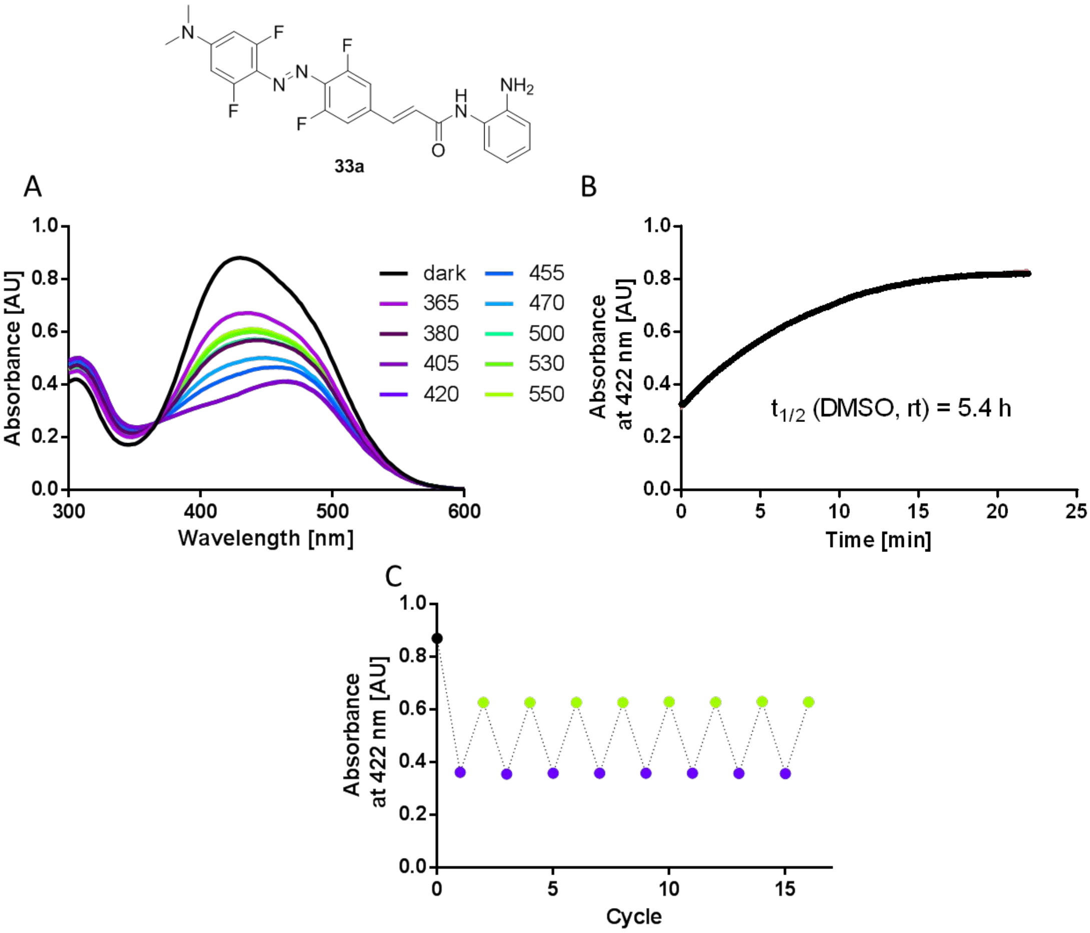

Figure S14. Photochemical properties of **33a** in DMSO. (A) UV-Vis spectra of **33a** (50 µM) under different light conditions. (B) Half-life estimation of *cis*-**33a** (50 µM) after irradiation (420 nm) following its absorbance at 422 nm. (C) Absorbance at 422 nm of **33a** (50 µM) after multiple illumination cycles (420 – 550 nm, 2 min each). In all cases, illumination at 420 nm was done at 13 mW/cm^2, and 550 nm at 12 mW/cm^2.

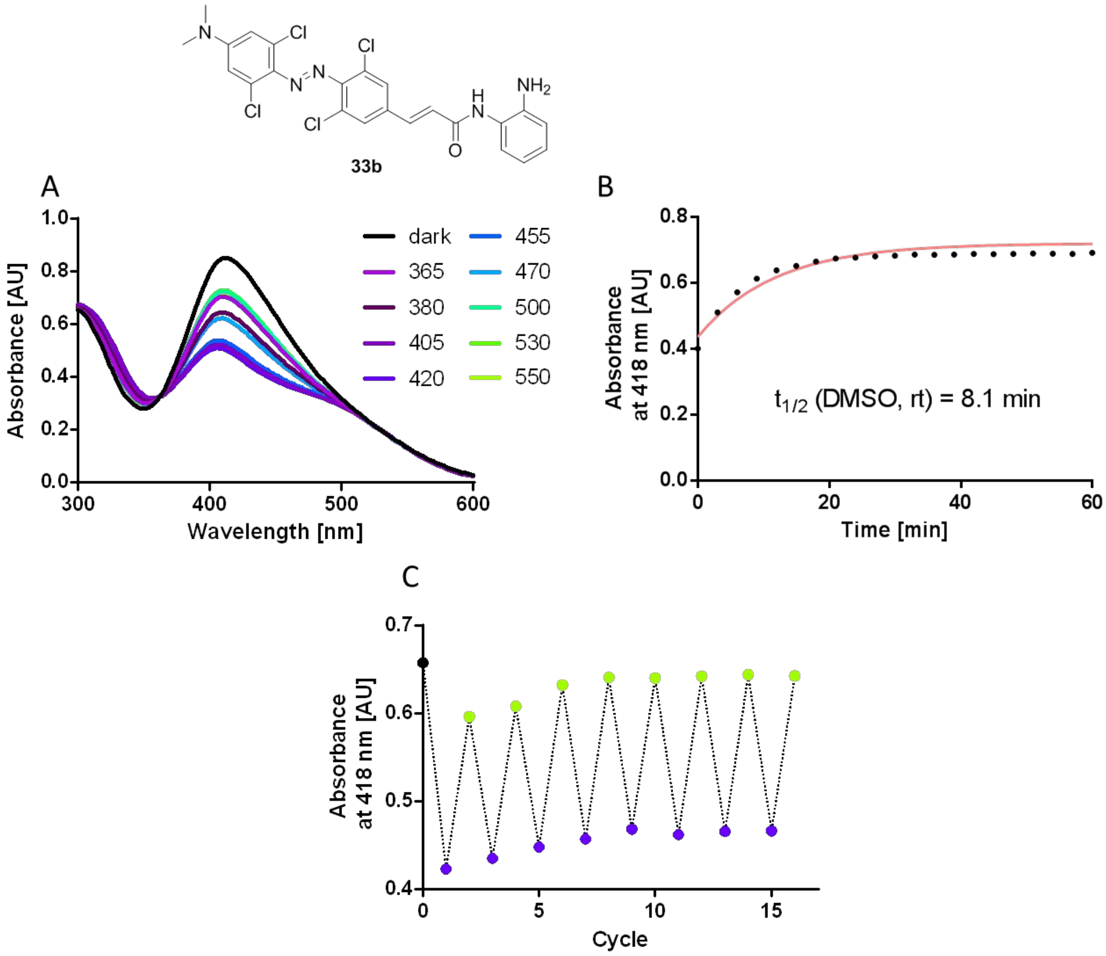

Figure S15. Photochemical properties of **33b** in DMSO. (A) UV-Vis spectra of **33b** (50 µM) under different light conditions. (B) Half-life estimation of *cis*-**33b** (50 µM) after irradiation (420 nm) following its absorbance at 422 nm. (C) Absorbance at 422 nm of **33b** (50 µM) after multiple illumination cycles (420 – 550 nm, 2 min each). In all cases, illumination at 420 nm was done at 13 mW/cm^2, and 550 nm at 12 mW/cm^2.

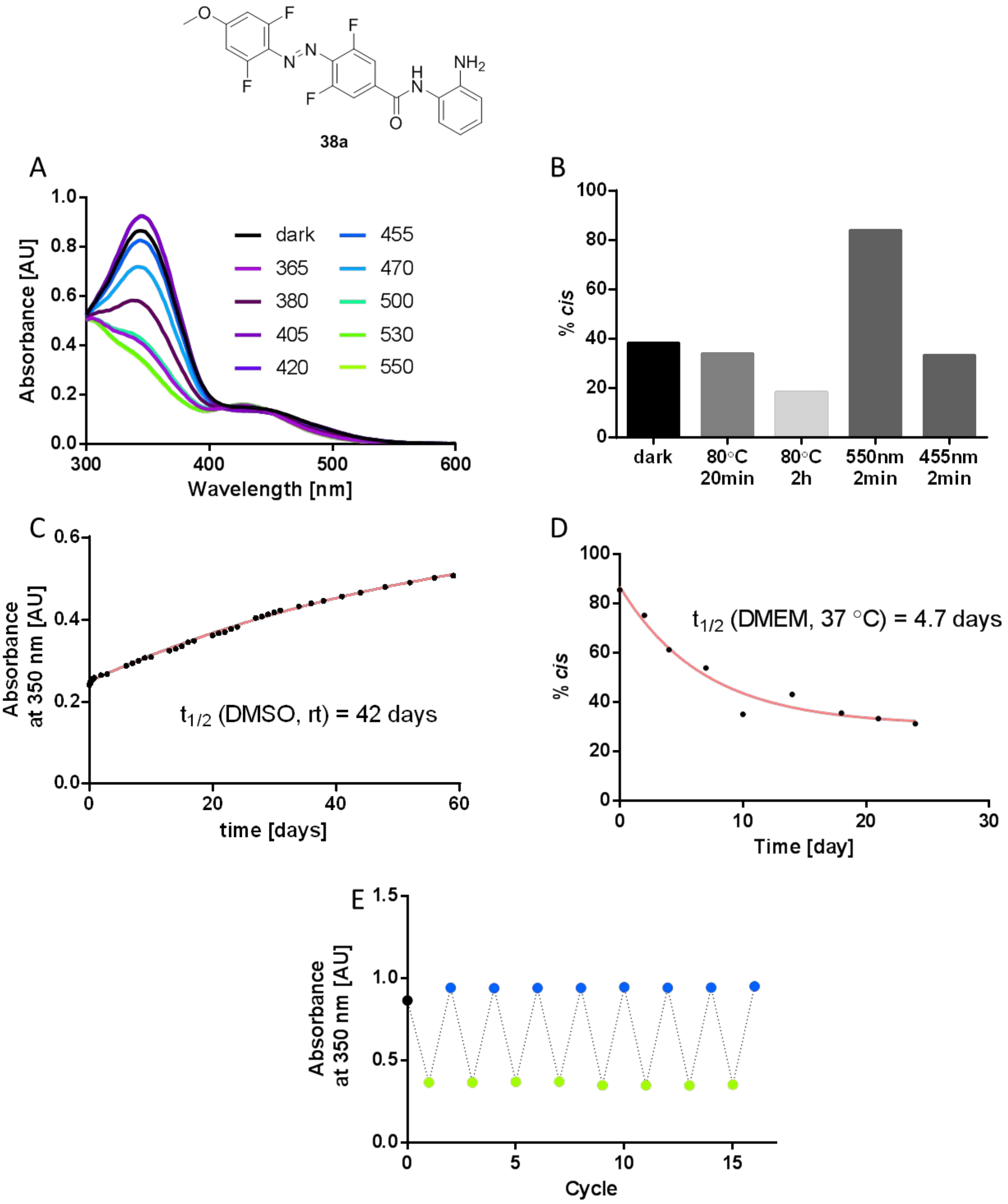

Figure S16. Photochemical properties of **38a** in DMSO. (A) UV-Vis spectra of **38a** (50 µM) under different light conditions. (B) Quantification of E/Z composition of **38a** (100 µM) after different temperature and light conditions, measured by HPLC at the isosbestic point (290 nm). (C) Half-life estimation of *cis*-**38a** (50 µM) after irradiation (550 nm) following its absorbance at 350 nm. (D) Half-life estimation by HPLC of *cis*-**38a** in DMEM at 37 °C (10 µM) after irradiation (550 nm). (E) Absorbance at 350 nm of **38a** (50 µM) after multiple illumination cycles (550 – 455 nm, 2 min each). In all cases, illumination at 455 nm was done at 9 mW/cm^2, and 550 nm at 12 mW/cm^2.

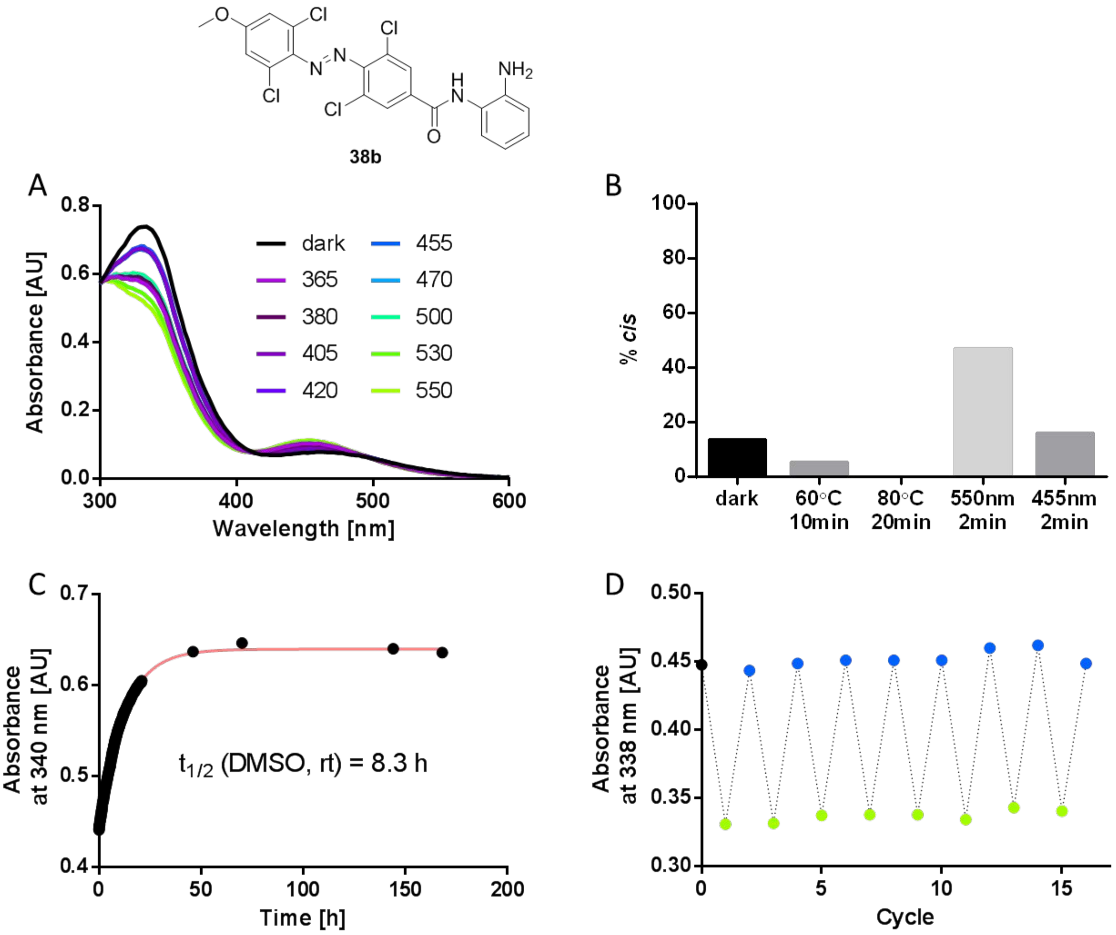

Figure S17. Photochemical properties of **38b** in DMSO. (A) UV-Vis spectra of **38b** (50 µM) under different light conditions. (B) Quantification of E/Z composition of **38b** (100 µM) after different temperature and light conditions, measured by HPLC at the isosbestic point (295 nm). (C) Half-life estimation of *cis*-**38b** (50 µM) after irradiation (550 nm) following its absorbance at 340 nm. (D) Absorbance at 338 nm of **38b** (50 µM) after multiple illumination cycles (550 – 455 nm, 2 min each). In all cases, illumination at 455 nm was done at 9 mW/cm^2, and 550 nm at 12 mW/cm^2.

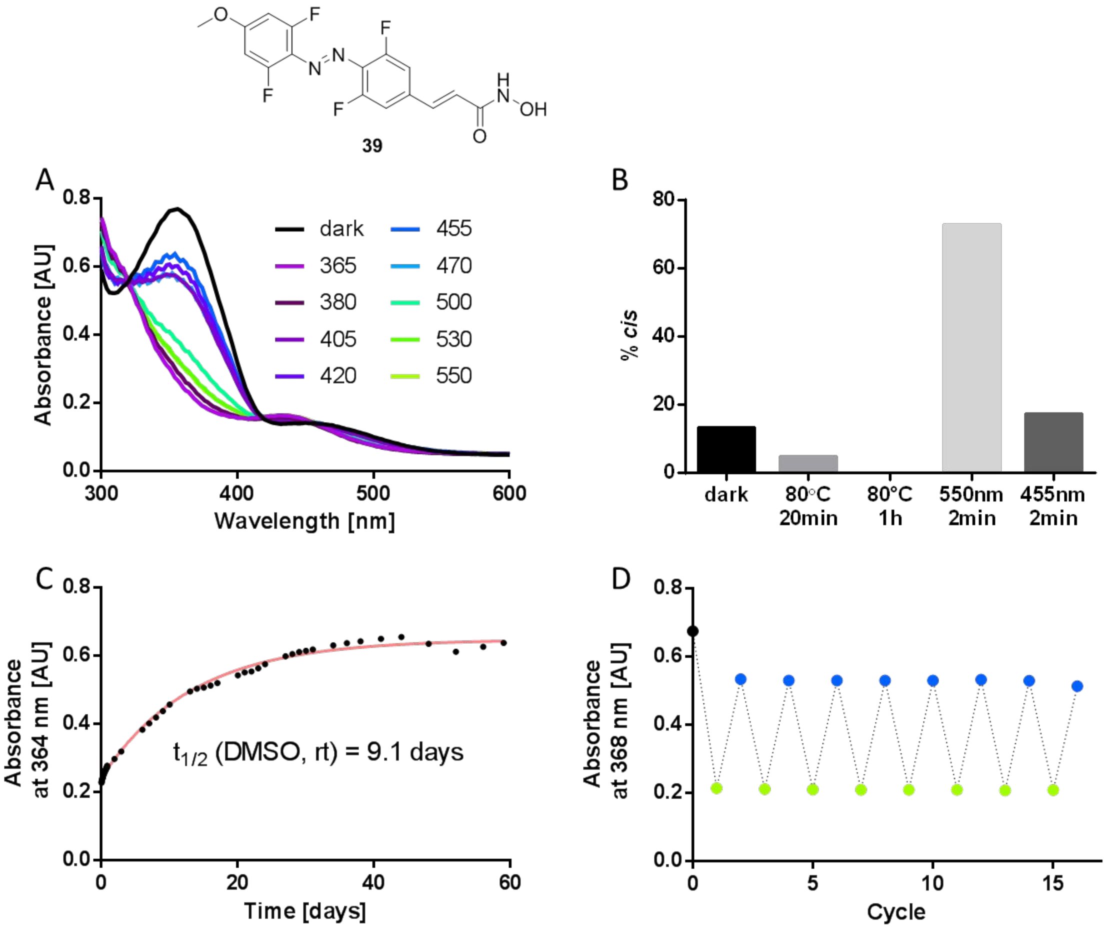

Figure S18. Photochemical properties of **39** in DMSO. (A) UV-Vis spectra of **39** (100 µM) under different light conditions. (B) Quantification of E/Z composition of **39** (100 µM) after different temperature and light conditions, measured by HPLC at the isosbestic point (321 nm). (C) Half-life estimation of *cis*-**39** (50 µM) after irradiation (550 nm) following its absorbance at 364 nm. (D) Absorbance at 368 nm of **39** (100 µM) after multiple illumination cycles (550 – 455 nm, 2 min each). In all cases, illumination at 455 nm was done at 9 mW/cm^2, and 550 nm at 12 mW/cm^2.

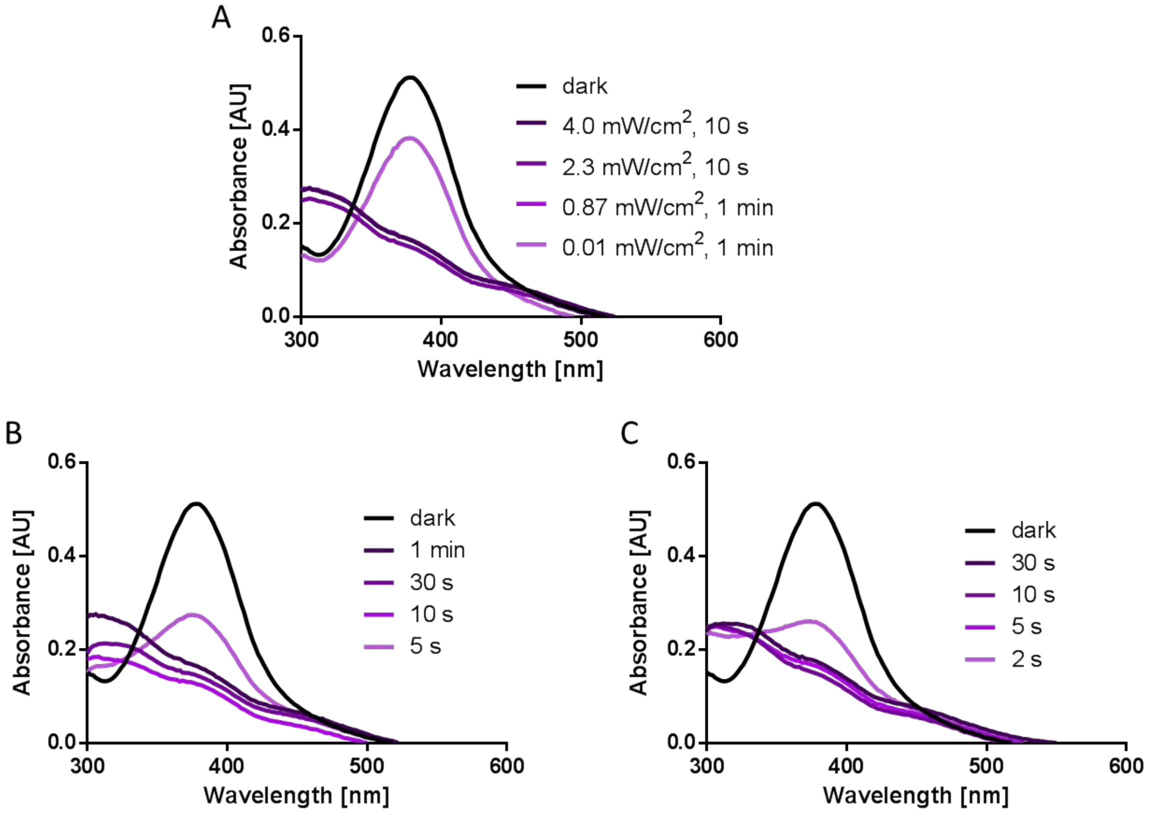

Figure S19. UV-Vis spectra of **11** (25 µM) in 50% DMSO/HDAC after illumination with 365 nm at (A) different intensities for 10 s or 1 min, (B) 0.87 mW/cm^2 for different times, and (C) 2.3 mW/cm^2 for different times.

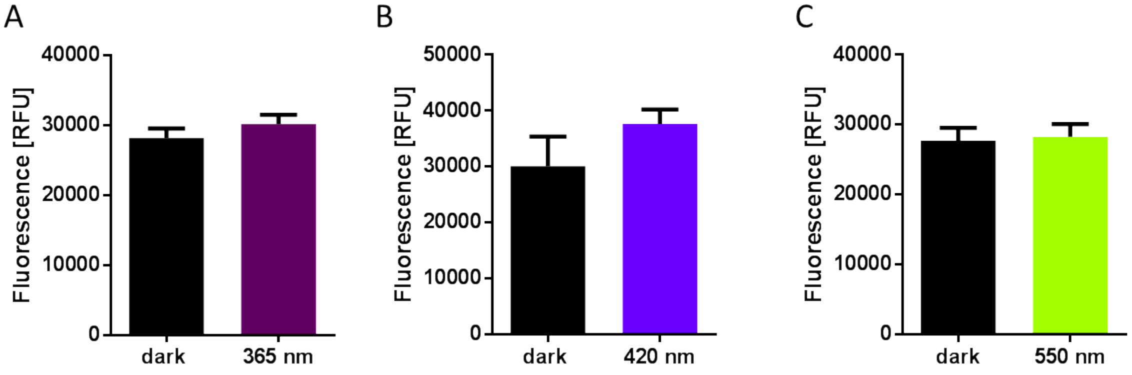

Figure S20. Activity of non-treated recombinant HDAC1 under either dark or illumination conditions, at (A) 365 nm, 2 mW/cm^2; (B) 420 nm, 8 mW/cm^2; and (C) 550 nm, 7 mW/cm^2.

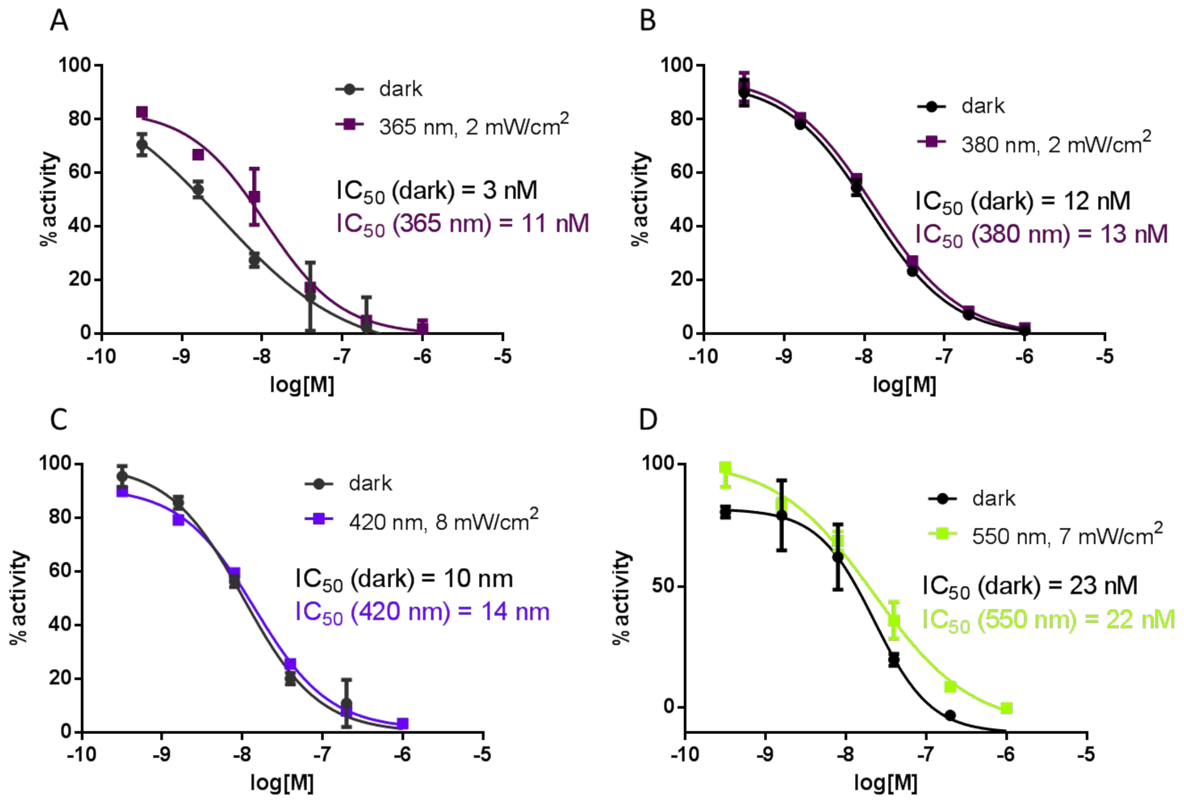

Figure S21. Inhibition of recombinant HDAC1 by SAHA under either dark or illumination conditions, at (A) 365 nm, 2 mW/cm^2; (B) 380 nm, 2 mW/cm^2; (C) 420 nm, 8 mW/cm^2; and (D) 550 nm, 7 mW/cm^2.

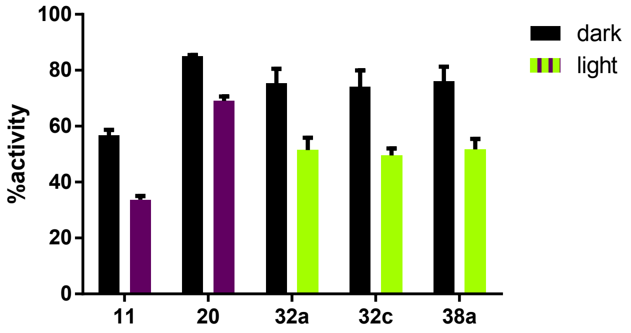

Figure S22. Activity of representative compounds in a whole-cell HDAC inhibition assay at 50 µM under either dark or pre-illumination (380 or 550 nm) conditions.

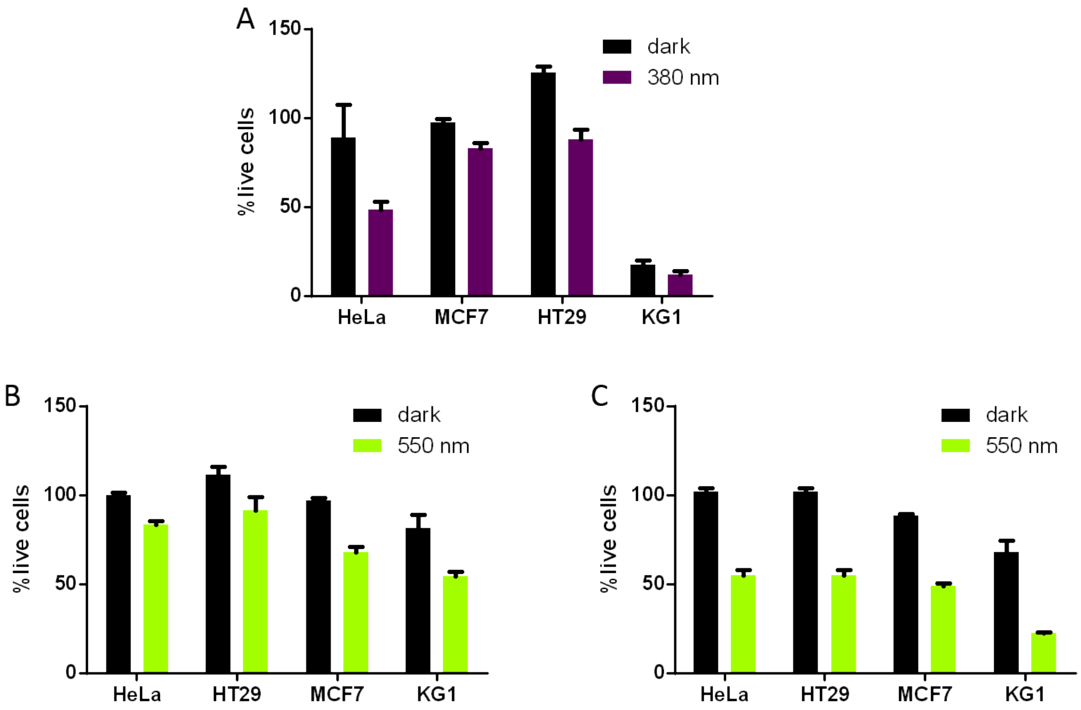

Figure S23. Effect of 50 µM of (A) **11**, (B) **32a** and (C) **38a** on HeLa, HT29, MCF7 and KG1 cells under either dark or pre-illumination (380 or 550 nm) conditions, measured by MTS (HeLa, HT29, MCF7) and CellTiterGlo (KG1) assays.

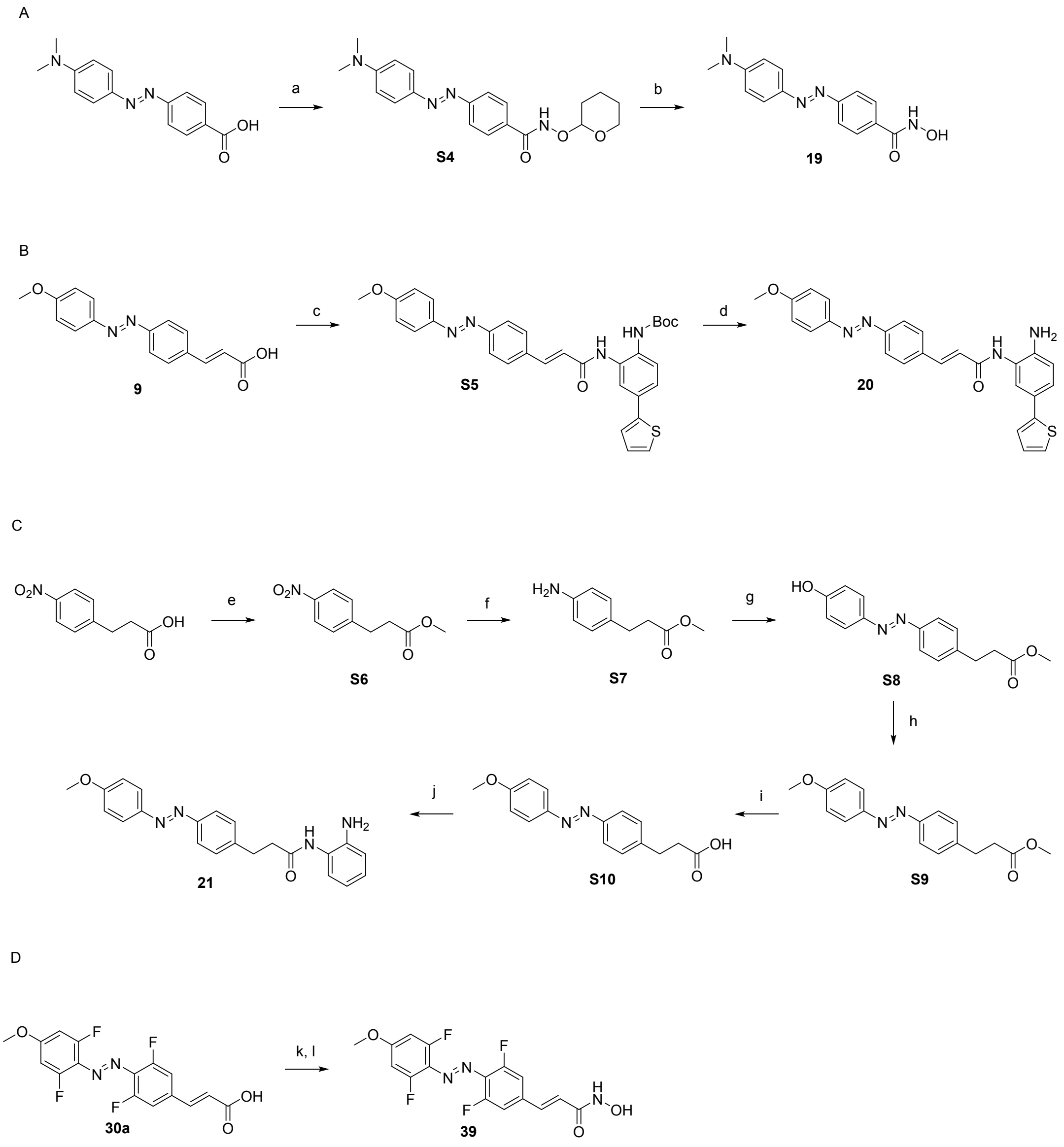

Scheme S1. Synthesis of compounds **19**, **20**, **21** and **39**. Reagents and conditions: (a) *O*-THP-hydroxylamine, DIPEA, EDC, HOBt, DMF, rt, 18 h, 65%; (b) HCl, dioxane, rt, 30 min, 66%; (c) *tert*-butyl (2-amino-4-(thiophen-2-yl)phenyl)carbamate,[1] EDC, HOBt, DMF, rt, 18 h, 38%; (d) TFA, DCM, rt, 2 h, 63%; (e) H_2SO_4, MeOH, 80 °C, 2 h, quant.; (f) H_2, Pd/C, MeOH, rt, 5 h, 96%; (g) (i) $NaNO_2$, HCl, MeOH/H_2O, -5 °C to rt, 10 min; (ii) phenol, KOH, -5 °C to rt, 18 h, quant.; (h) MeI, K_2CO_3, acetone, 50 °C, 1 h, 84%; (i) NaOH, MeOH, 50 °C, 30 min, 94%; (j) benzene-1,2-diamine, DIPEA, EDC, HOBt, DMF, rt, 18 h, 18%; (k) *O*-THP-hydroxylamine, DIPEA, EDC, HOBt, DMF, rt, 18 h; (l) TFA, DCM, rt, 2 h, 25% over 2 steps.

(*E*)-*N*-(2-Aminophenyl)-3-(4-((*E*)-(4-methoxyphenyl)diazenyl)phenyl)acrylamide (11)

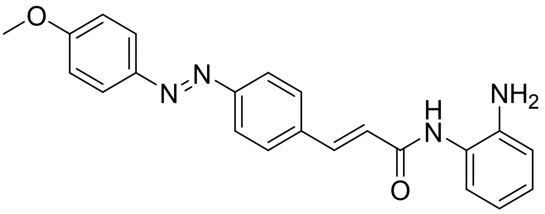

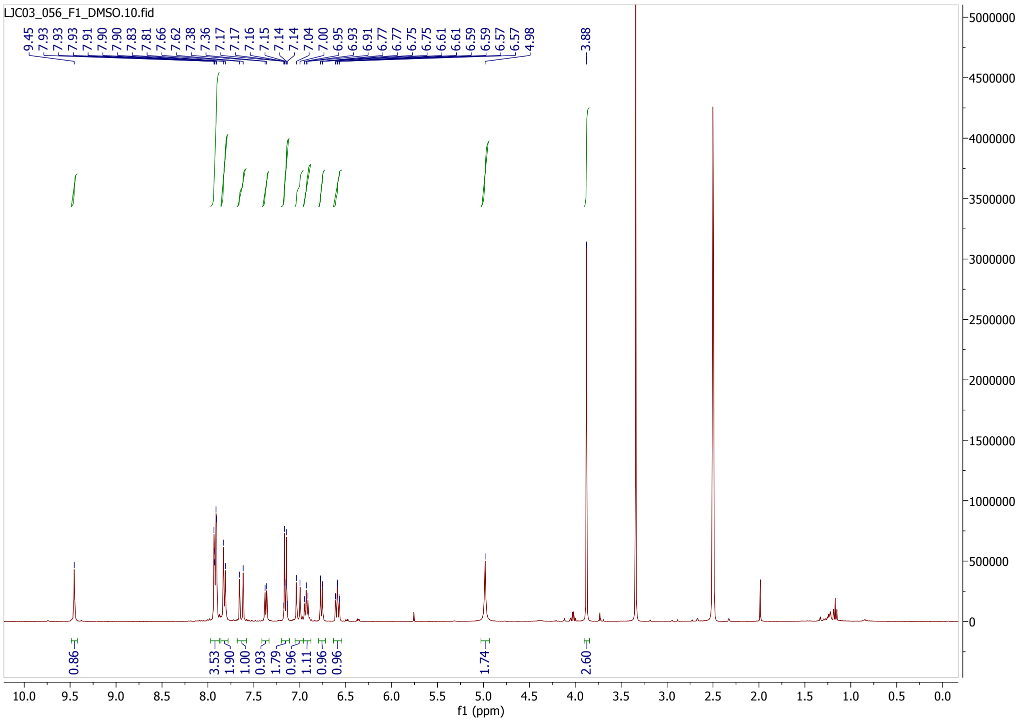

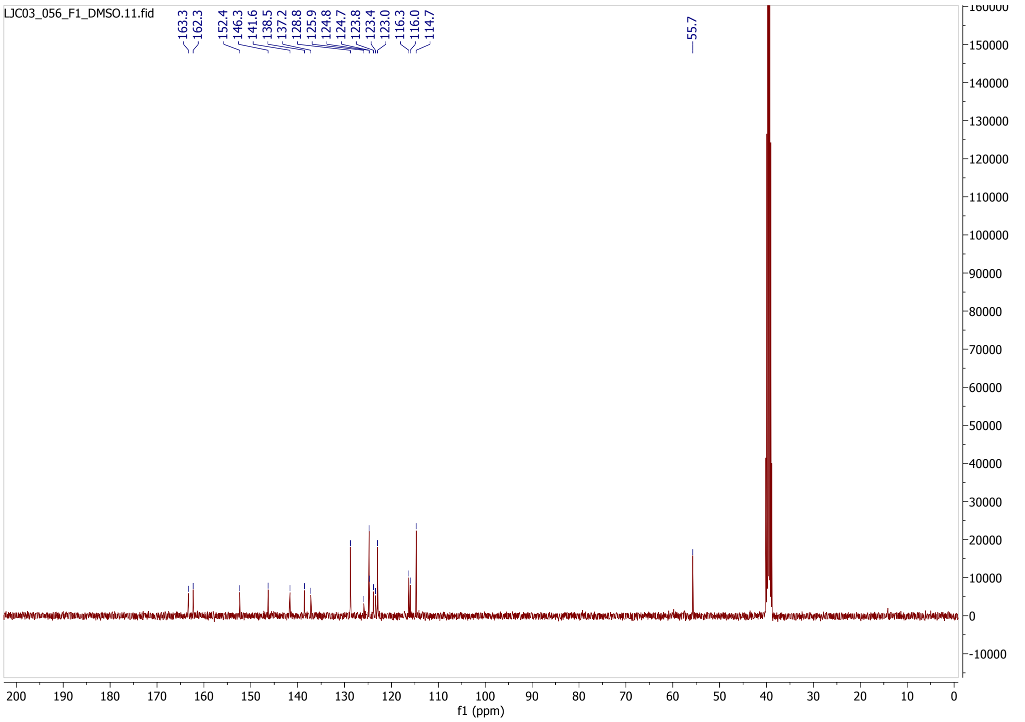

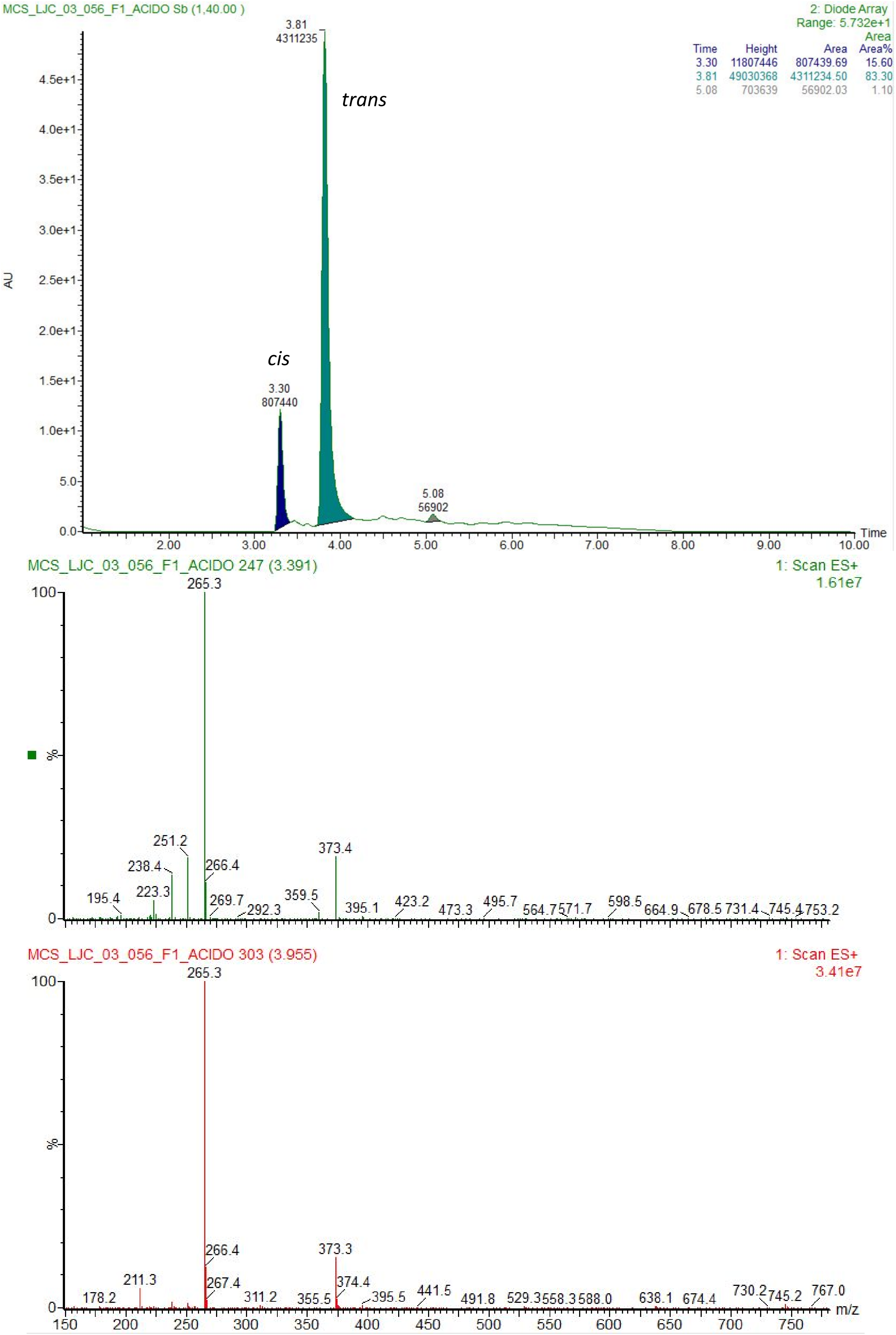
MCS_LJC_03_056_F1_ACIDO Sb (1,40.00)
2: Diode Array
Range: 5.732e+1
Time Height Area Area%
3.30 11807446 807439.69 15.60
3.81 49030368 4311234.50 83.30
5.08 703639 56902.03 1.10
3.81
4311235
trans
cis
3.30
807440
5.08
56902
AU
Time
MCS_LJC_03_056_F1_ACIDO 247 (3.391)
1: Scan ES+
1.61e7
265.3
251.2
238.4
266.4
373.4
195.4
223.3
269.7
292.3
359.5
395.1
423.2
473.3
495.7
564.7
571.7
598.5
664.9
678.5
731.4
745.4
753.2
MCS_LJC_03_056_F1_ACIDO 303 (3.955)
1: Scan ES+
3.41e7
265.3
266.4
373.3
211.3
267.4
374.4
178.2
311.2
355.5
395.5
441.5
491.8
529.3
558.3
588.0
638.1
674.4
730.2
745.2
767.0
m/z
%

(*E*)-*N*-(2-Aminophenyl)-3-(4-((*E*)-(4-(dimethylamino)phenyl)diazenyl)phenyl)acrylamide (12)

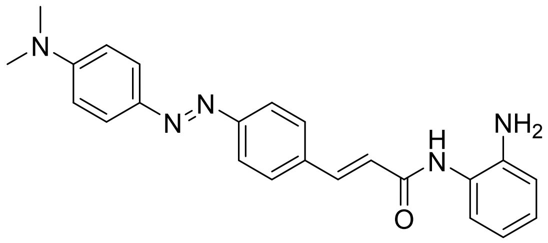

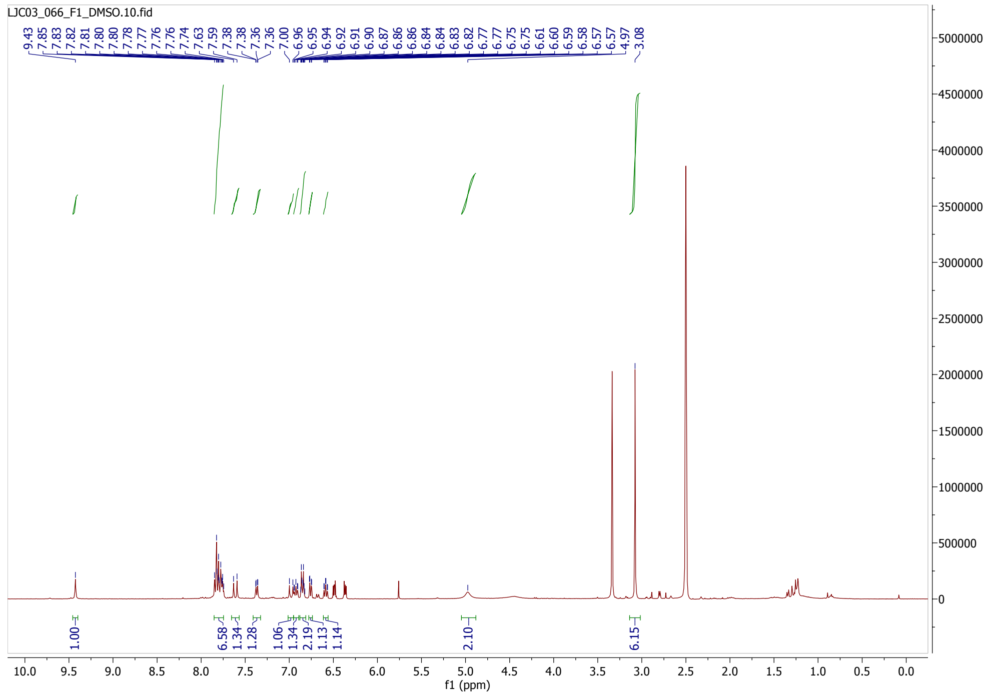

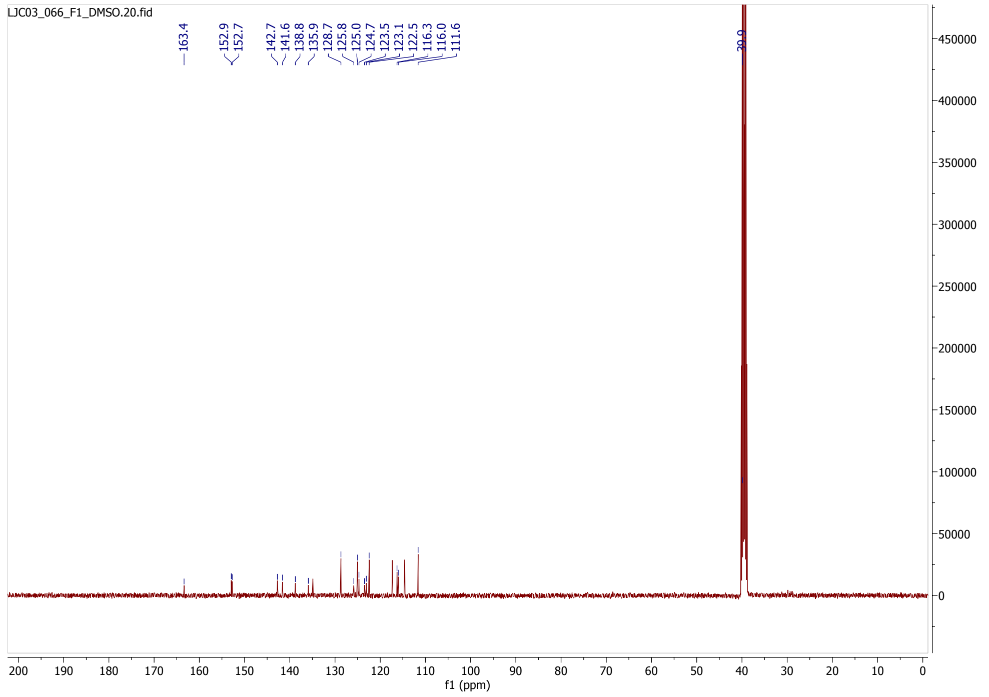

(*E*)-*N*-Hydroxy-4-((4-methoxyphenyl)diazenyl)benzamide (17)

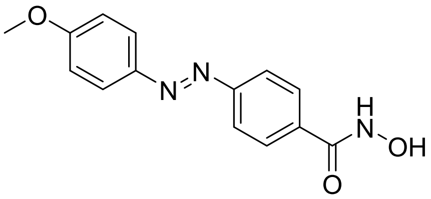

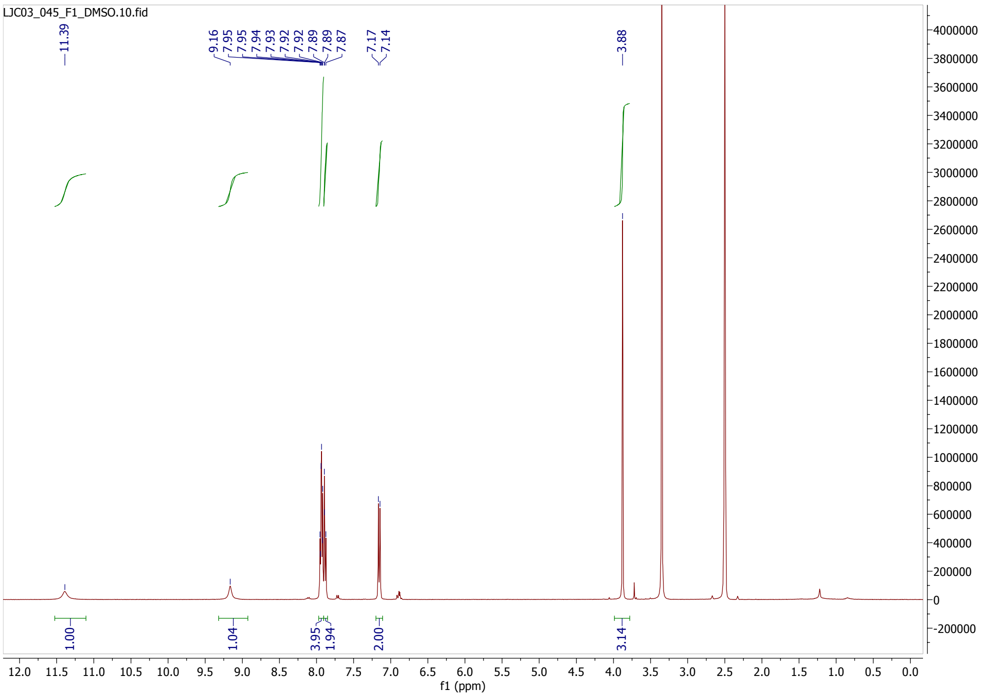

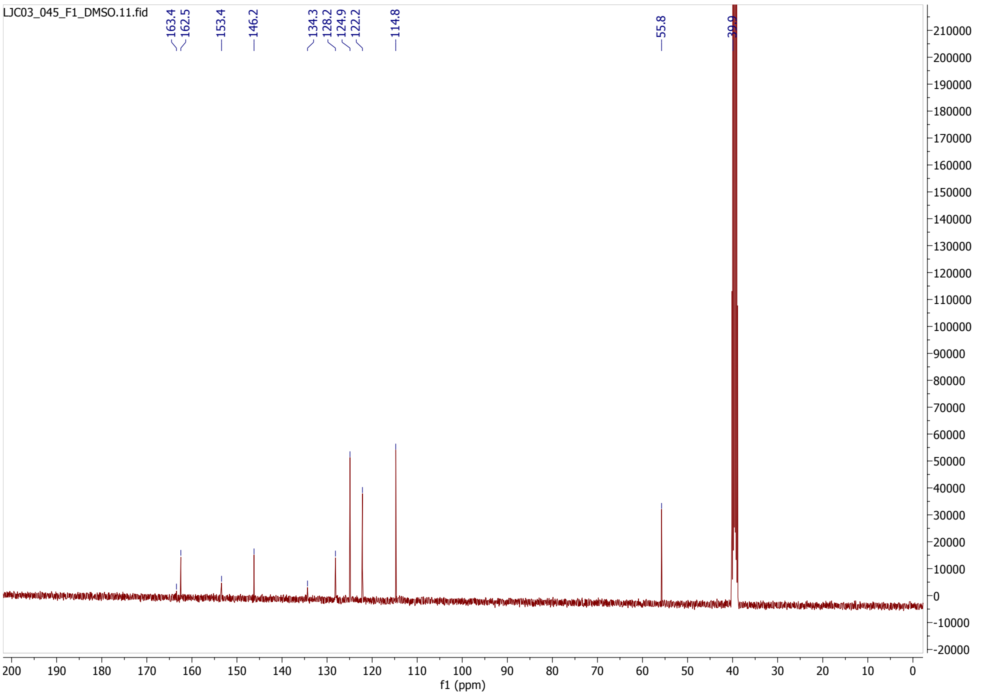

(*E*)-*N*-(2-Aminophenyl)-4-((4-methoxyphenyl)diazenyl)benzamide (18)

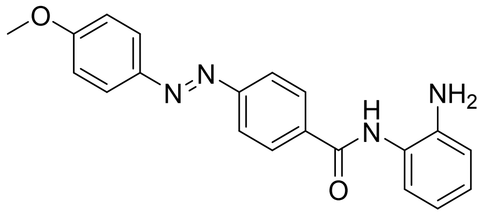

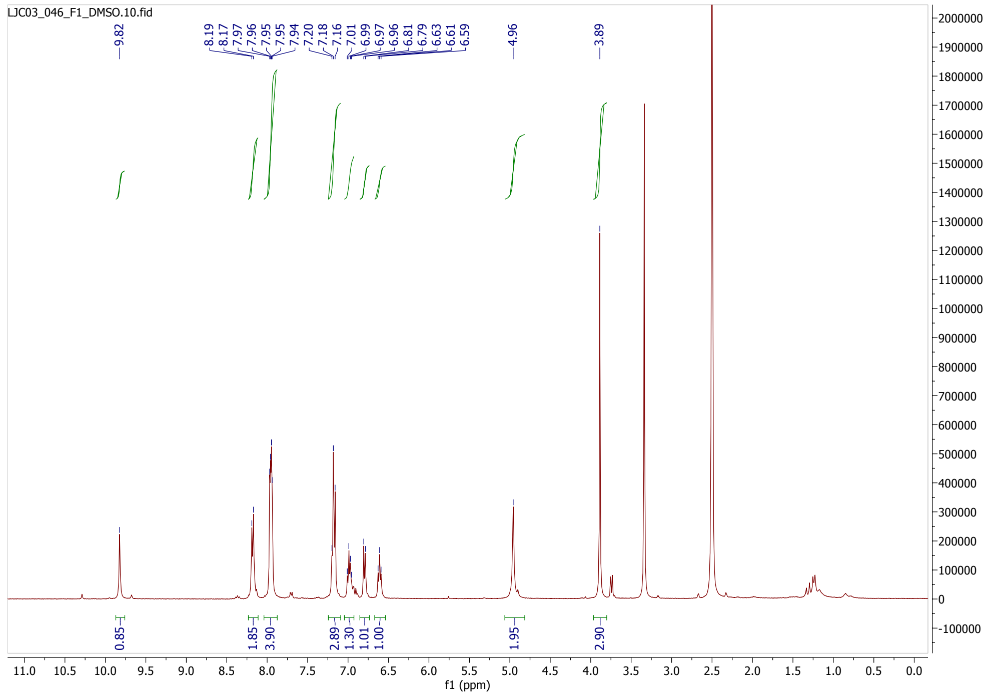

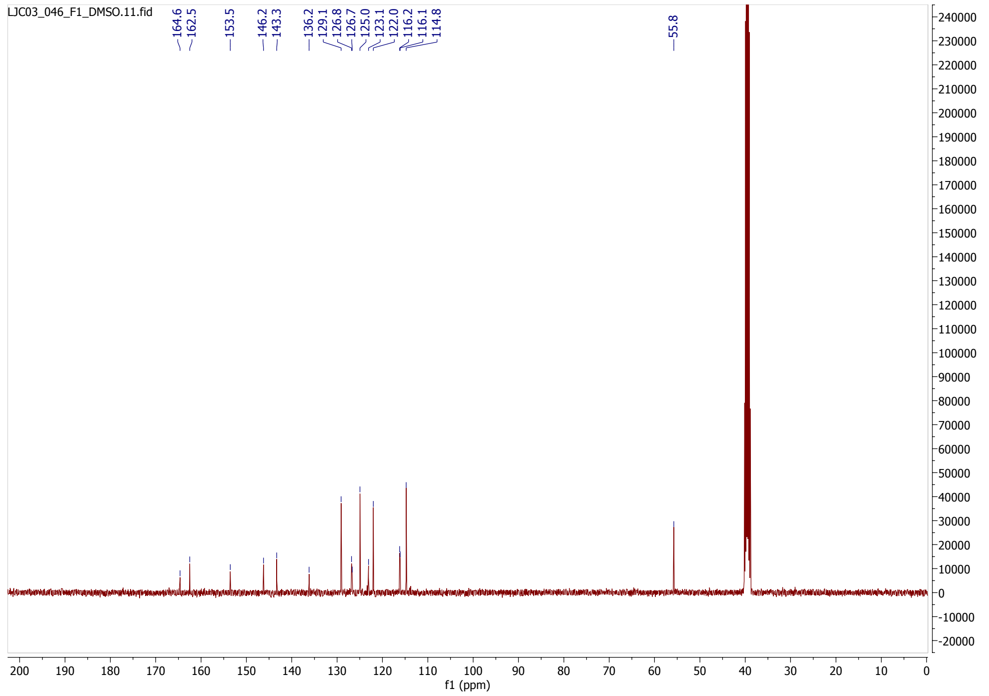

(*E*)-4-((4-(Dimethylamino)phenyl)diazenyl)-*N*-hydroxybenzamide (19)

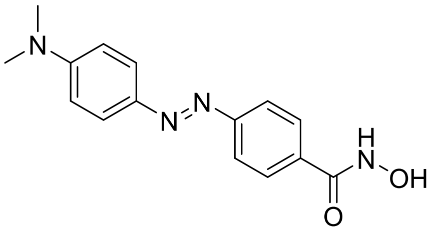

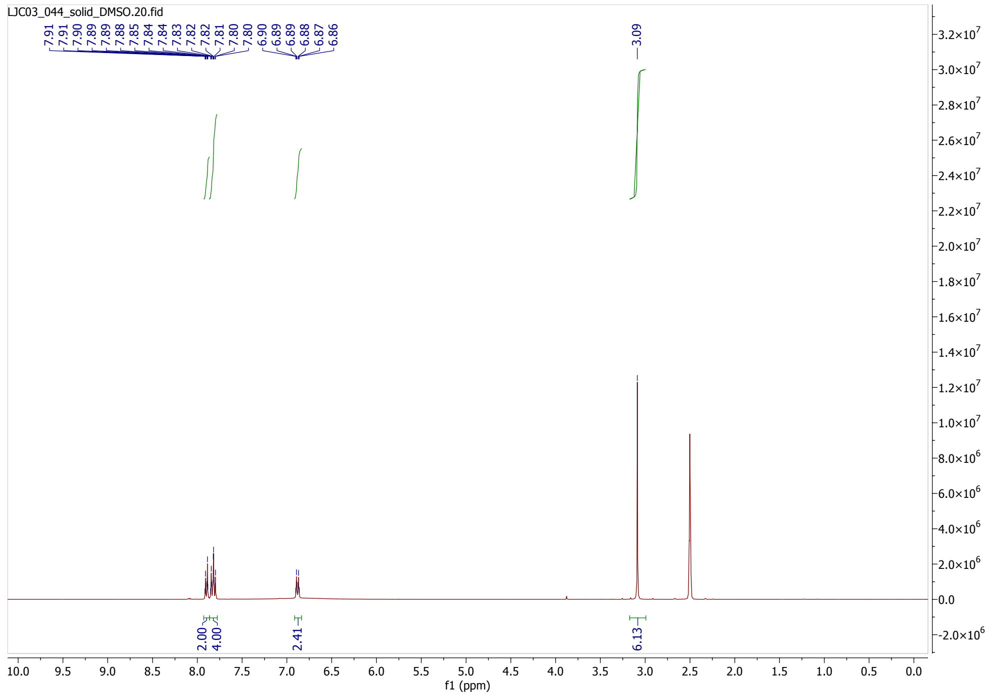

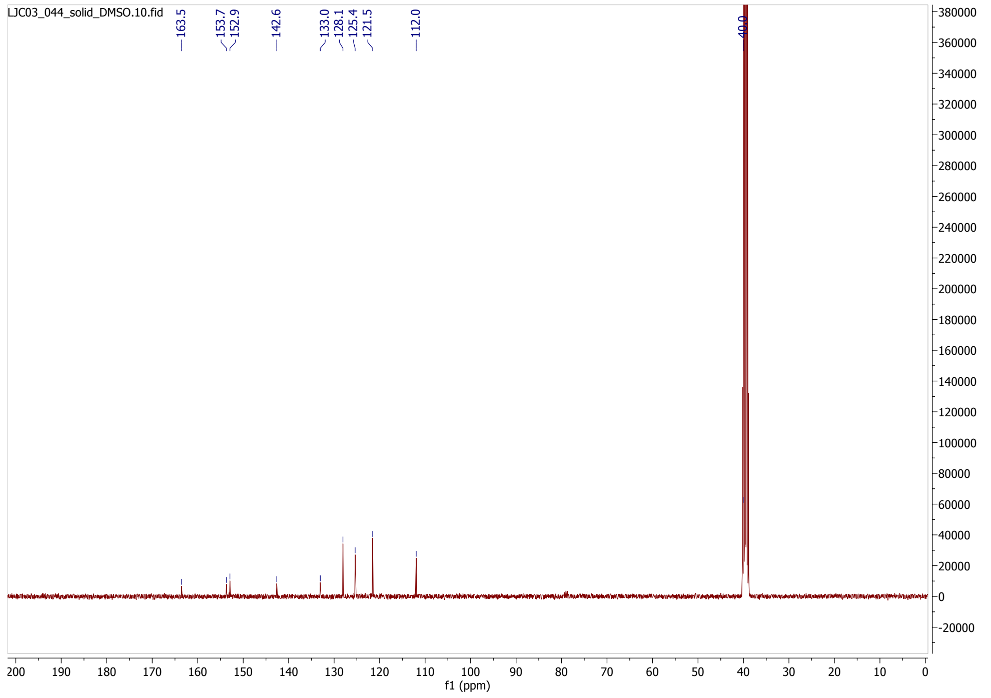

(*E*)-*N*-(2-Amino-5-(thiophen-2-yl)phenyl)-3-(4-((*E*)-(4-methoxyphenyl)diazenyl)phenyl)acrylamide (20)

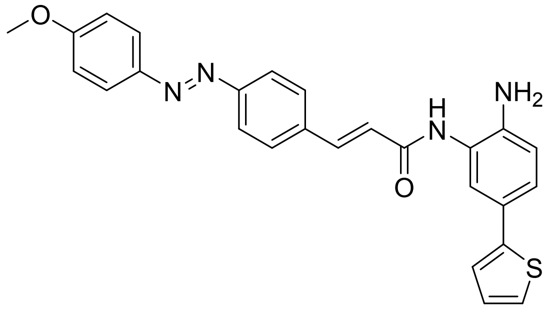

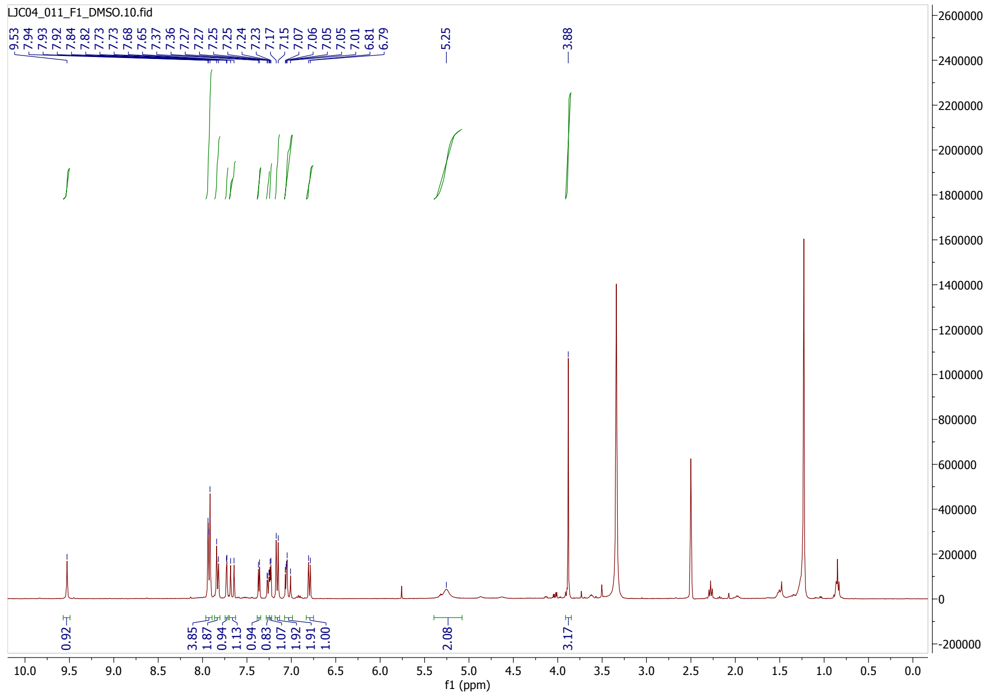

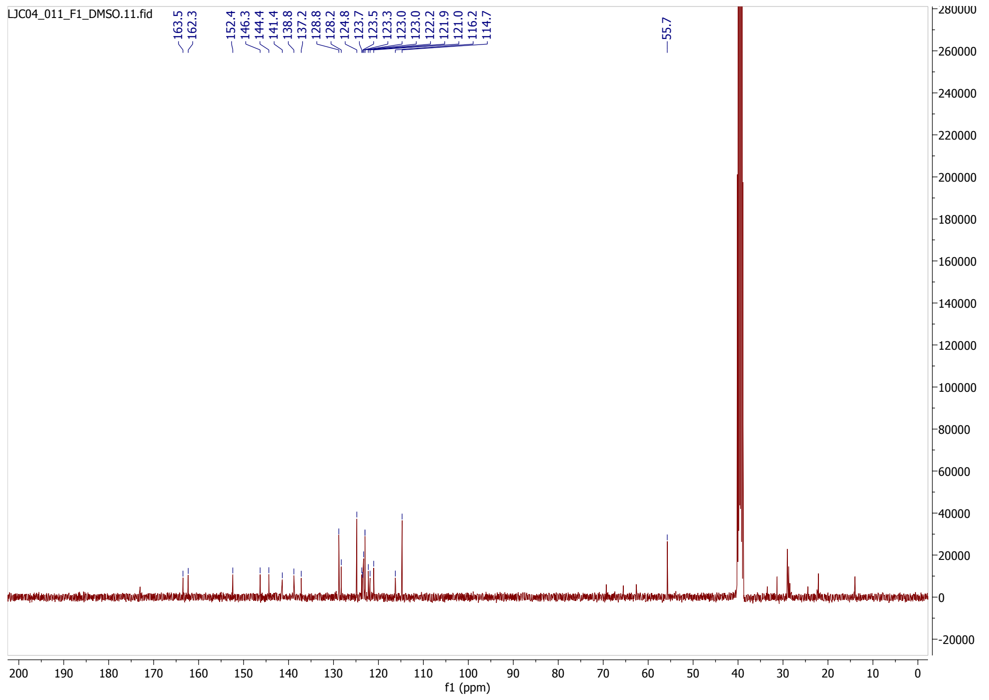

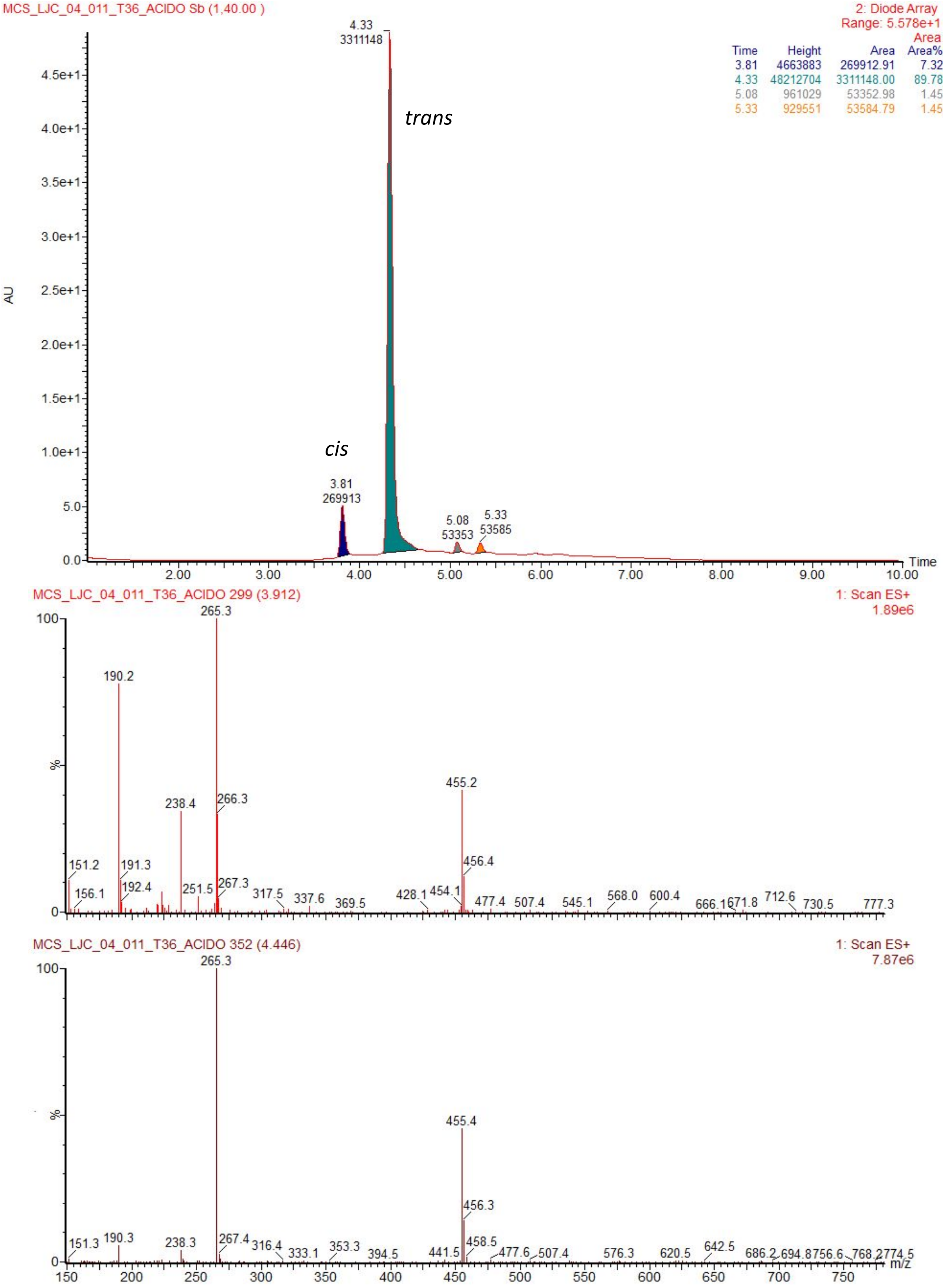
MCS_LJC_04_011_T36_ACIDO Sb (1,40.00)
2: Diode Array
Range: 5.578e+1
Time Height Area Area%
3.81 4663883 269912.91 7.32
4.33 48212704 3311148.00 89.78
5.08 961029 53352.98 1.45
5.33 929551 53584.79 1.45
4.33
3311148
trans
cis
3.81
269913
5.08
53353
5.33
53585
AU
Time
MCS_LJC_04_011_T36_ACIDO 299 (3.912)
1: Scan ES+
1.89e6
265.3
190.2
455.2
238.4
266.3
456.4
MCS_LJC_04_011_T36_ACIDO 352 (4.446)
1: Scan ES+
7.87e6
265.3
455.4
456.3
m/z

(*E*)-*N*-(2-Aminophenyl)-3-(4-((4-methoxyphenyl)diazenyl)phenyl)propanamide (21)

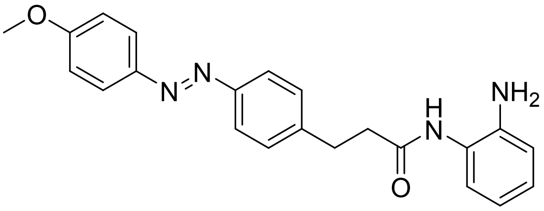

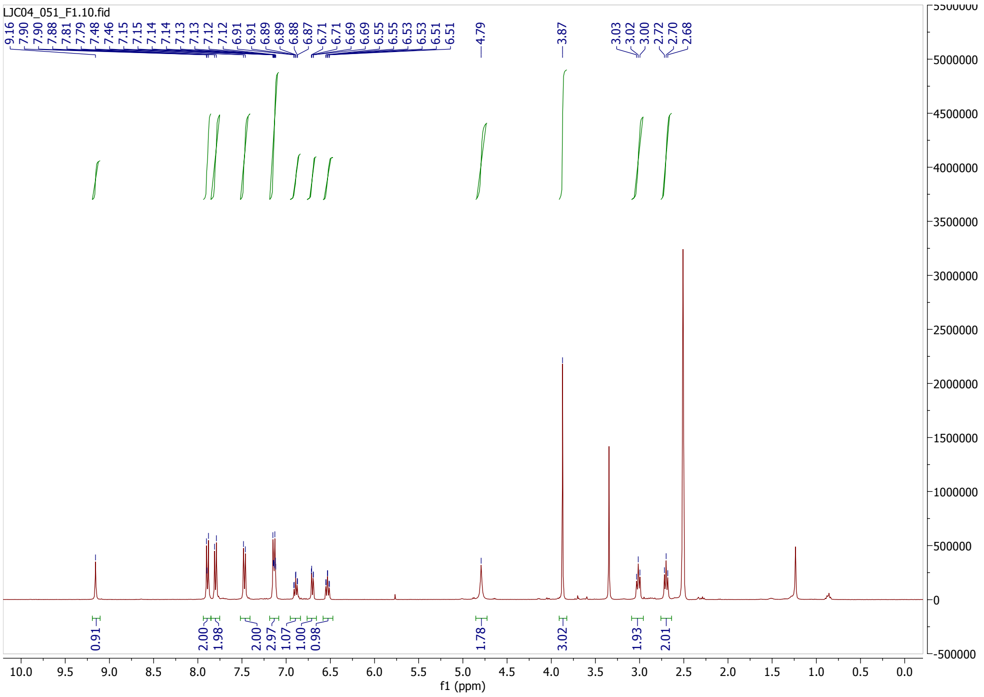

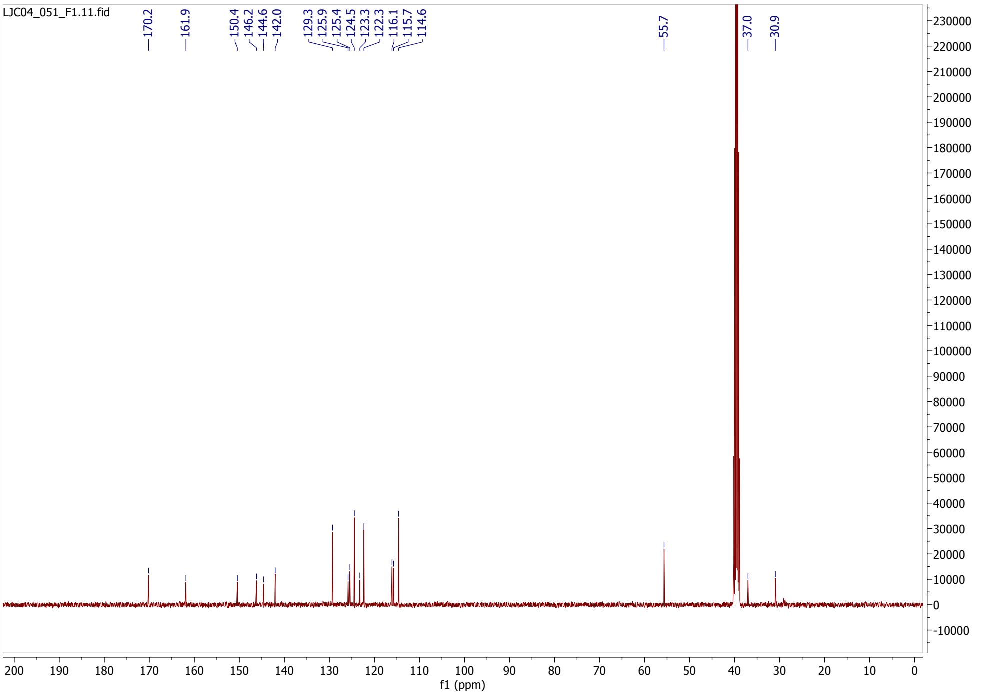

(*E*)-N-(2-Aminophenyl)-3-(4-((*E*)-(2,6-difluoro-4-methoxyphenyl)diazenyl)-3,5-difluorophenyl)acrylamide (32a)

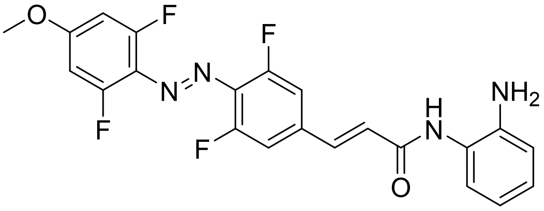

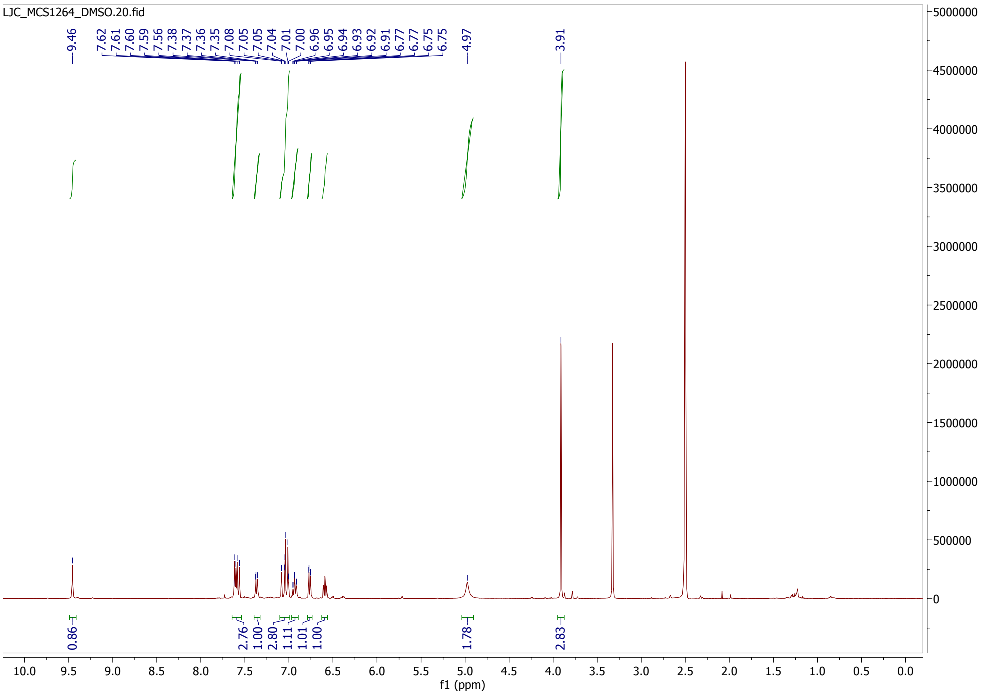

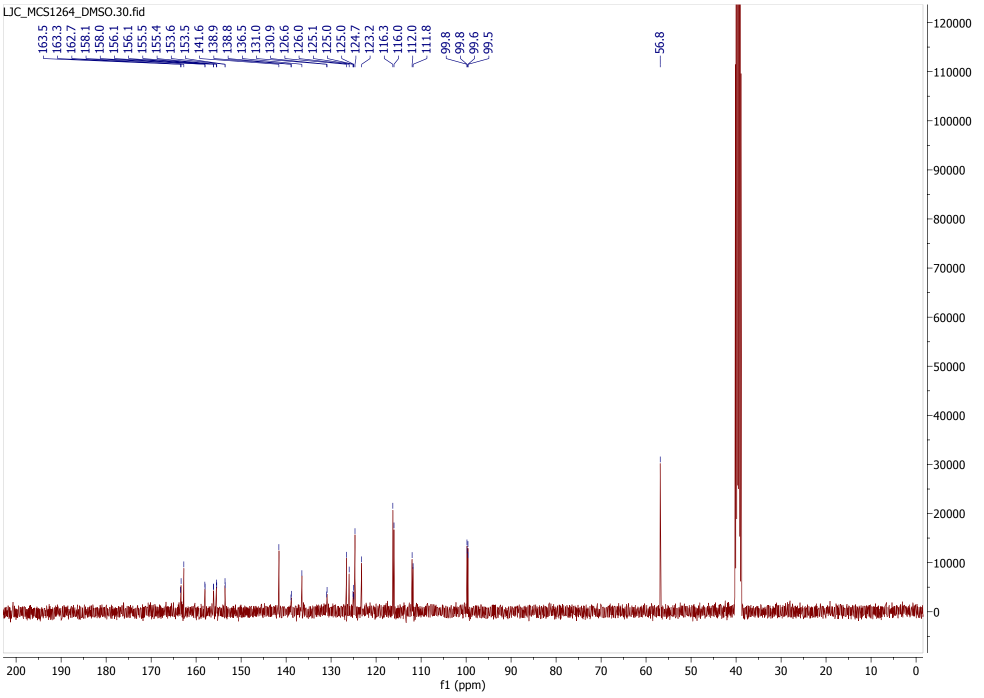

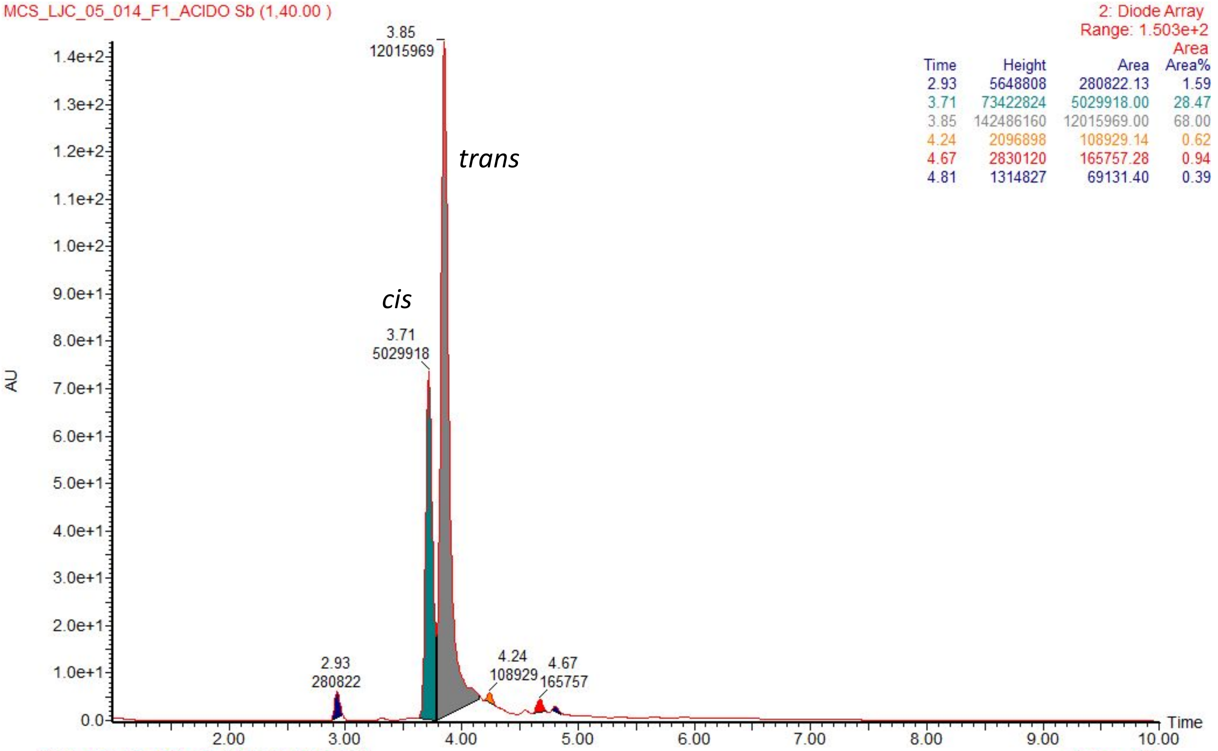
MCS_LJC_05_014_F1_ACIDO Sb (1,40.00)
2: Diode Array
Range: 1.503e+2
Time Height Area Area%
2.93 5648808 280822.13 1.59
3.71 73422824 5029918.00 28.47
3.85 142486160 12015969.00 68.00
4.24 2096898 108929.14 0.62
4.67 2830120 165757.28 0.94
4.81 1314827 69131.40 0.39
3.85 12015969
trans
cis
3.71 5029918
2.93 280822
4.24 108929
4.67 165757
AU
Time

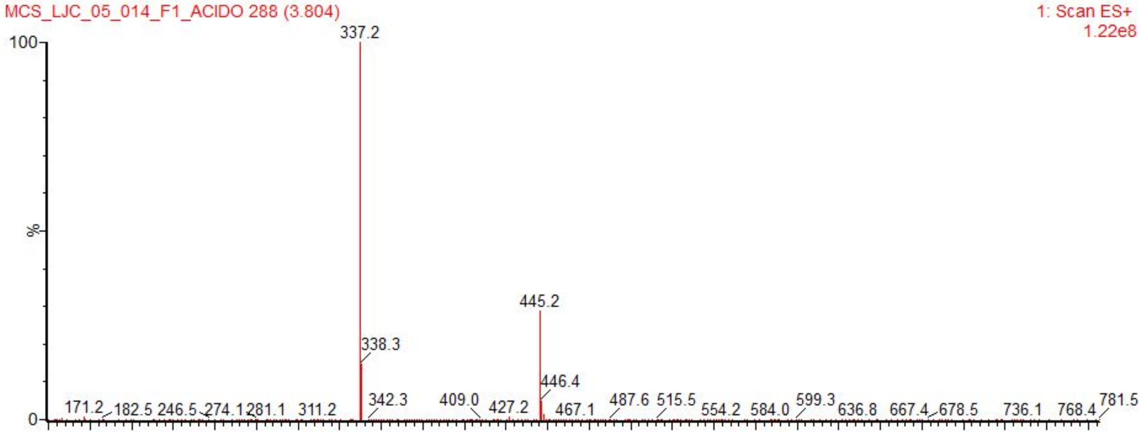
MCS_LJC_05_014_F1_ACIDO 288 (3.804)
1: Scan ES+
1.22e8
337.2
338.3
445.2
446.4
%

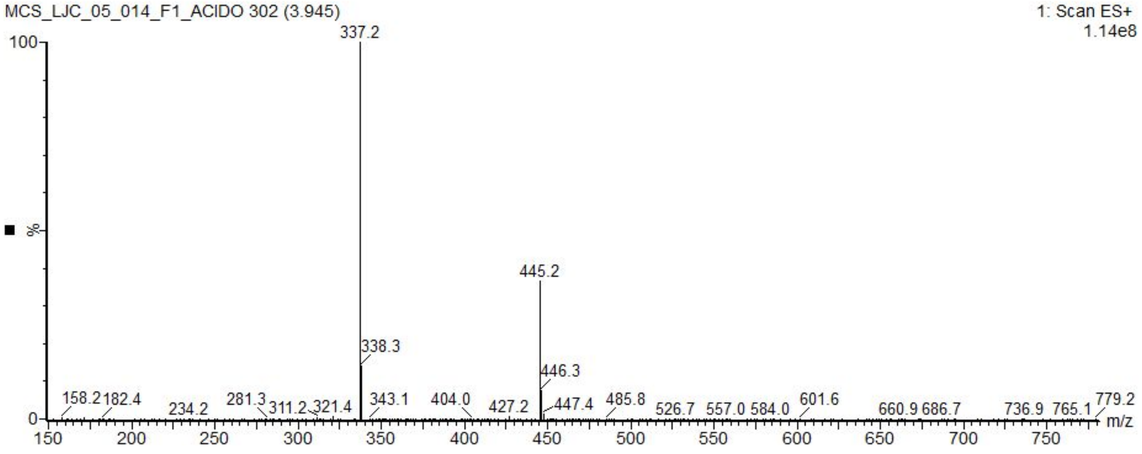
MCS_LJC_05_014_F1_ACIDO 302 (3.945)
1: Scan ES+
1.14e8
337.2
338.3
445.2
446.3
%
m/z

(*E*)-*N*-(2-Aminophenyl)-3-(3,5-dichloro-4-((*E*)-(2,6-dichloro-4-methoxyphenyl)diazenyl)phenyl)acrylamide (32b)

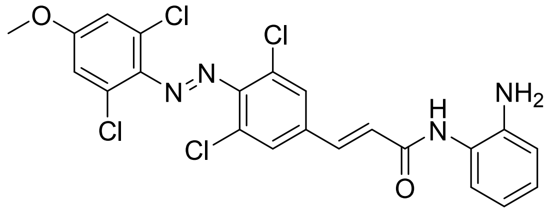

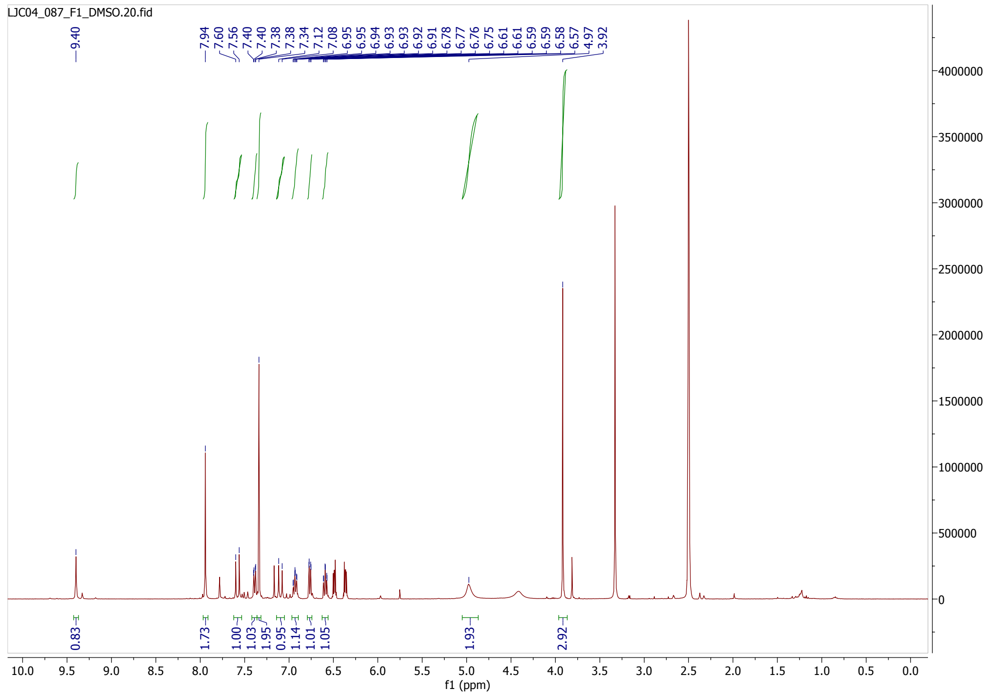

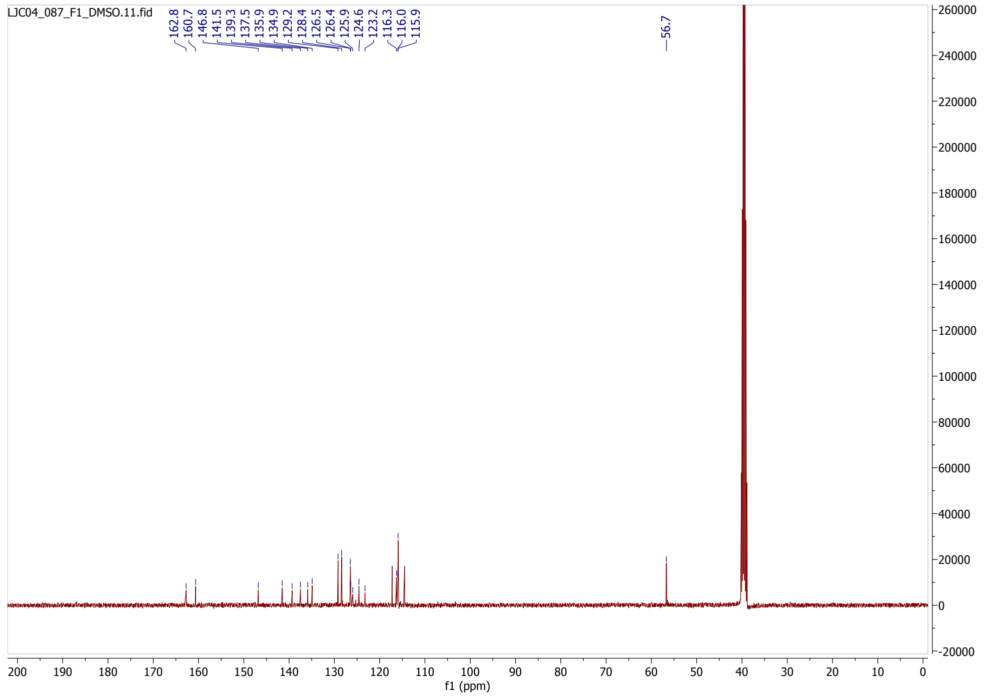

(*E*)-*N*-(2-Aminophenyl)-3-(3-chloro-4-((*E*)-(2-chloro-6-fluoro-4-methoxyphenyl)diazenyl)-5-fluorophenyl)acrylamide (32c)

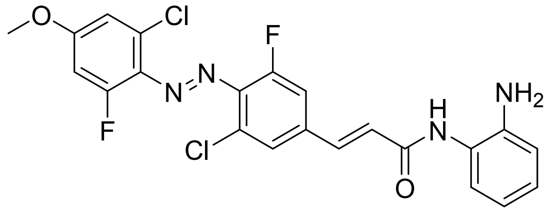

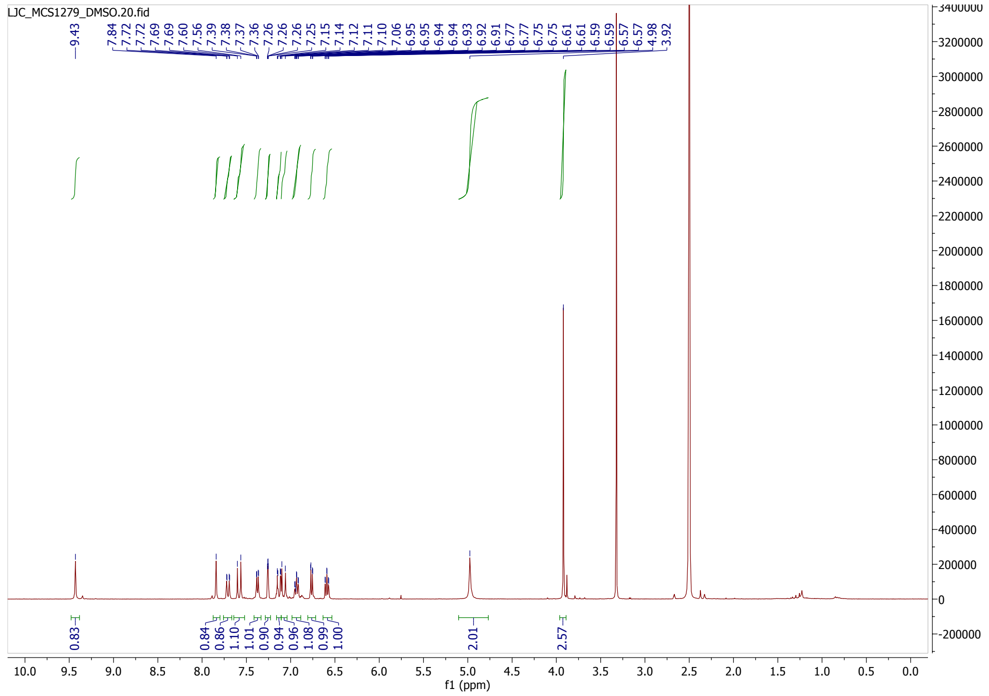

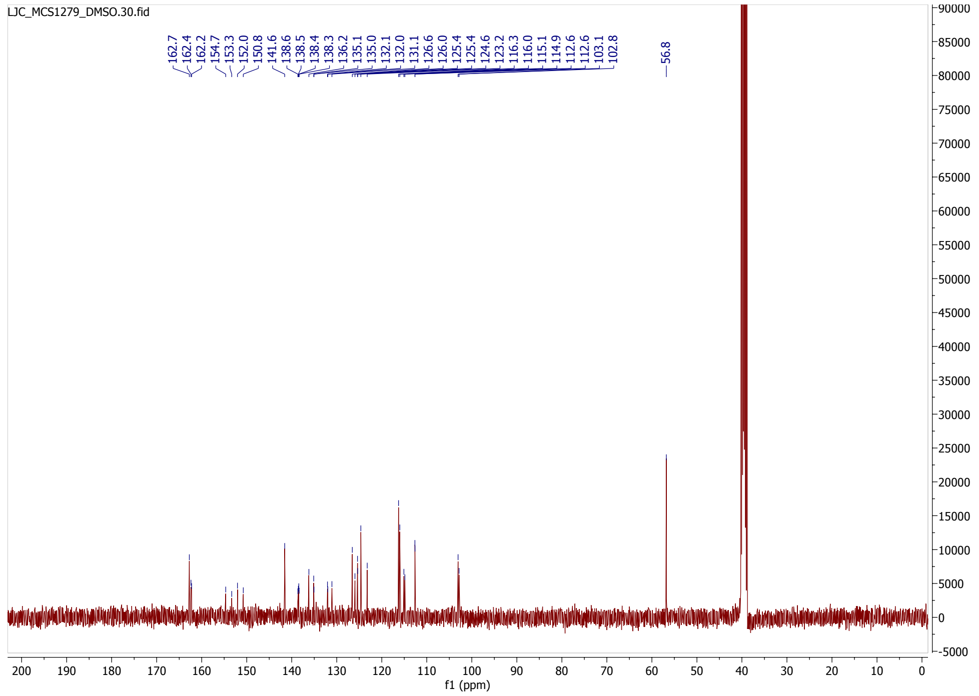

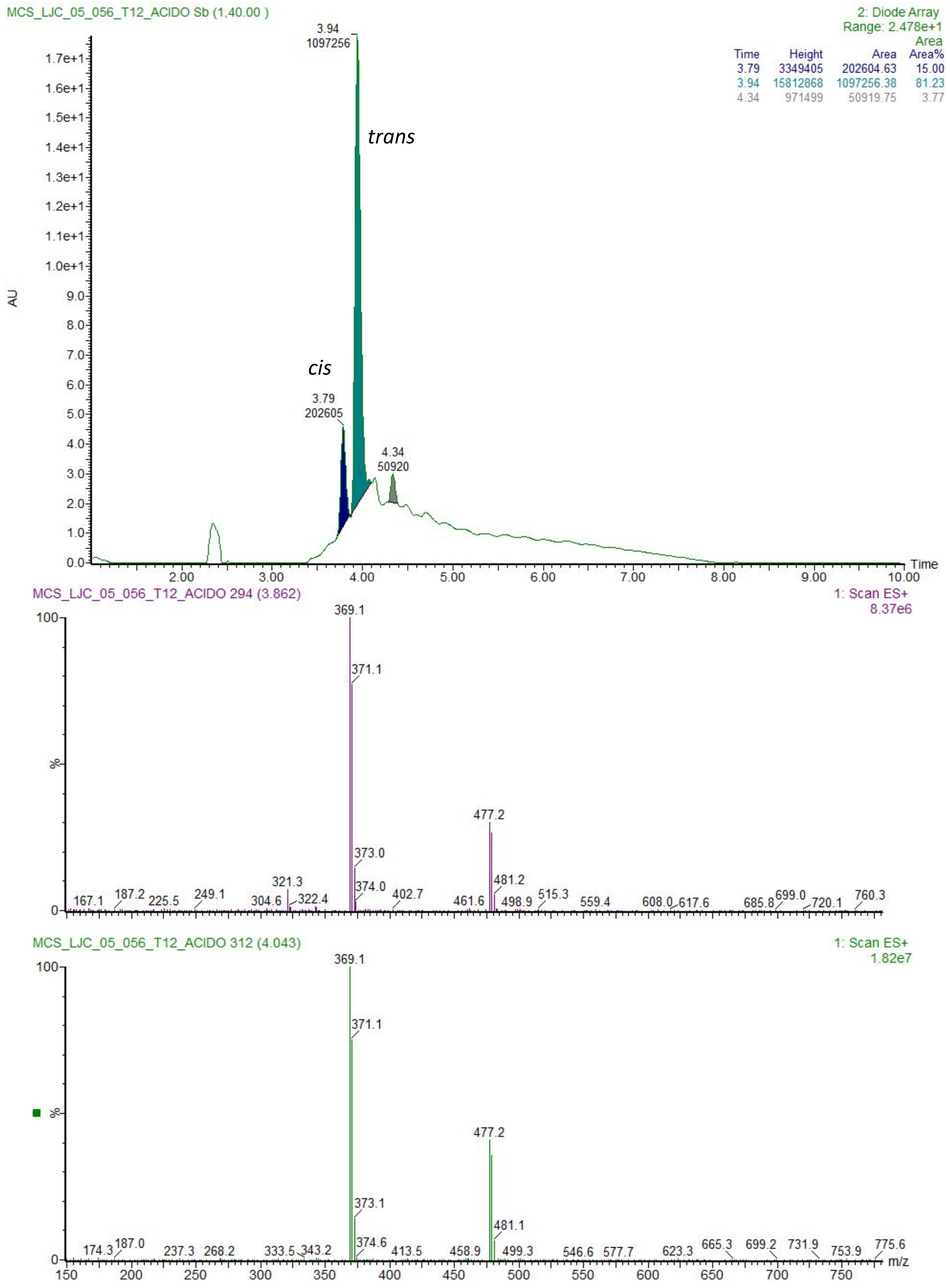
MCS_LJC_05_056_T12_ACIDO Sb (1,40.00)
2: Diode Array
Range: 2.478e+1
Time Height Area Area%
3.79 3349405 202604.63 15.00
3.94 15812868 1097256.38 81.23
4.34 971499 50919.75 3.77
3.94
1097256
trans
cis
3.79
202605
4.34
50920
AU
Time
MCS_LJC_05_056_T12_ACIDO 294 (3.862)
1: Scan ES+
8.37e6
369.1
371.1
477.2
373.0
321.3
481.2
374.0
MCS_LJC_05_056_T12_ACIDO 312 (4.043)
1: Scan ES+
1.82e7
369.1
371.1
477.2
373.1
481.1
374.6
m/z

(*E*)-*N*-(2-Aminophenyl)-3-(3-fluoro-4-((*E*)-(2-fluoro-4-methoxyphenyl)diazenyl)phenyl)acrylamide (32d)

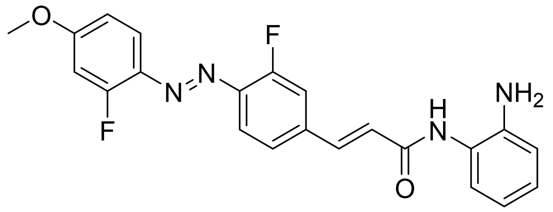

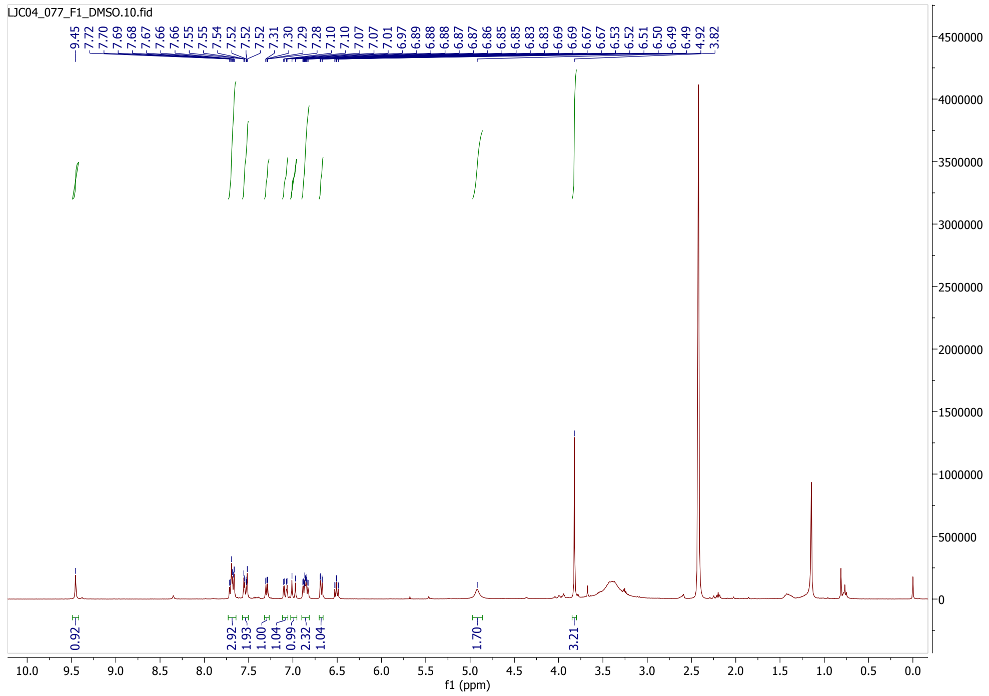

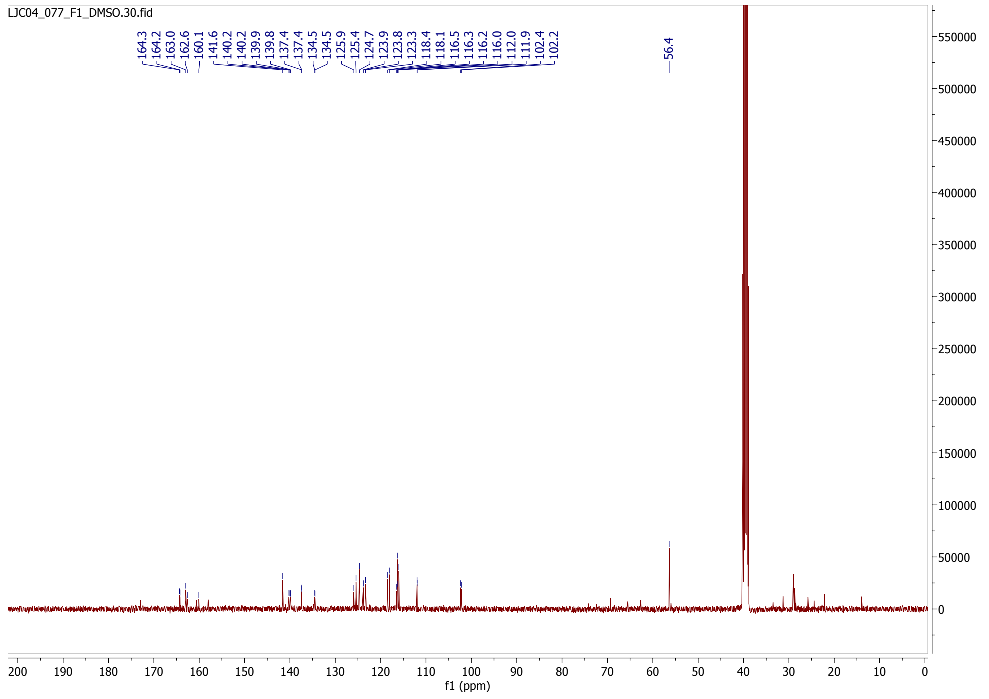

(*E*)-*N*-(2-Aminophenyl)-3-(3-chloro-4-((*E*)-(2-chloro-4-methoxyphenyl)diazenyl)phenyl)acrylamide (32e)

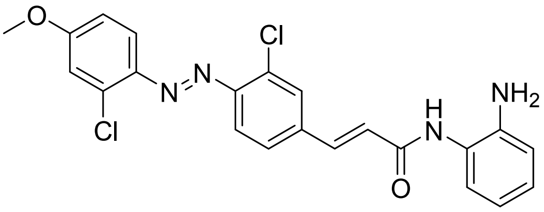

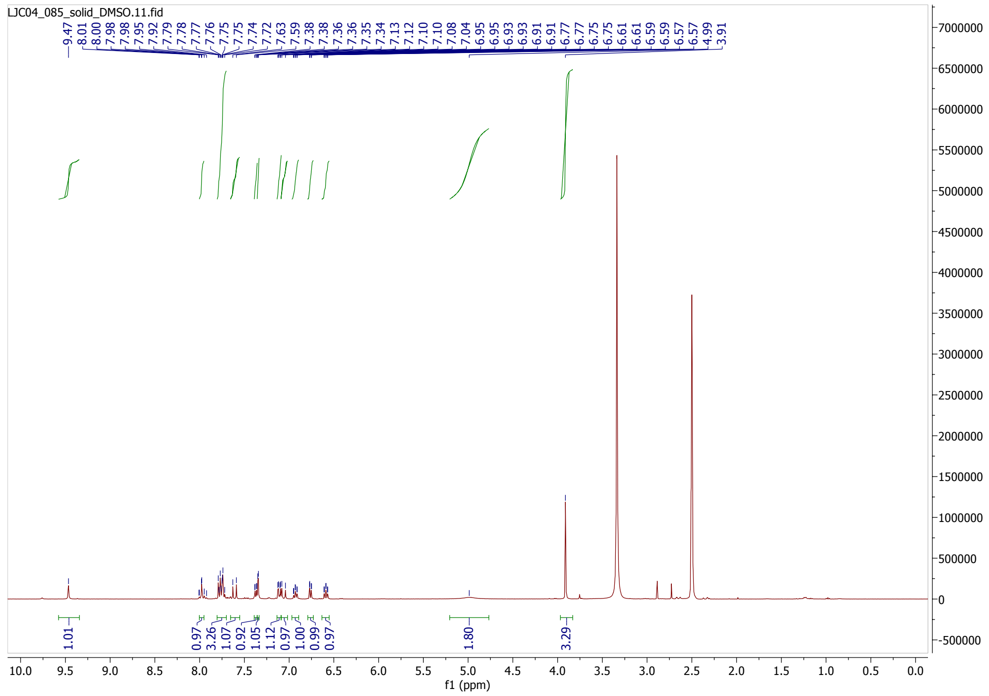

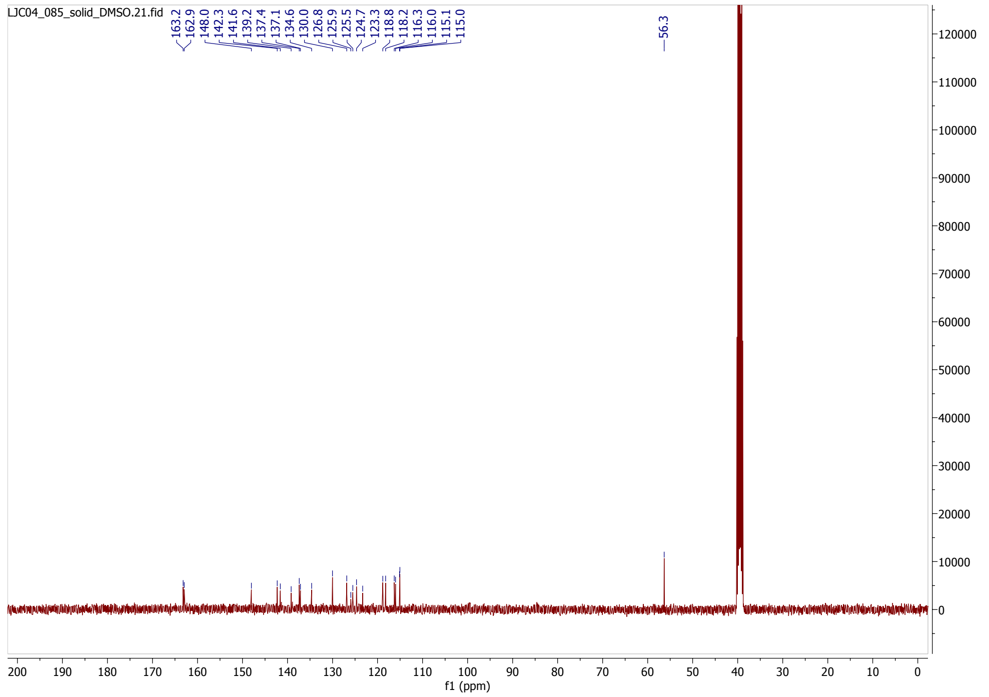

(*E*)-*N*-(2-Aminophenyl)-3-(4-((*E*)-(4-(dimethylamino)-2,6-difluorophenyl)diazenyl)-3,5-difluorophenyl)acrylamide (33a)

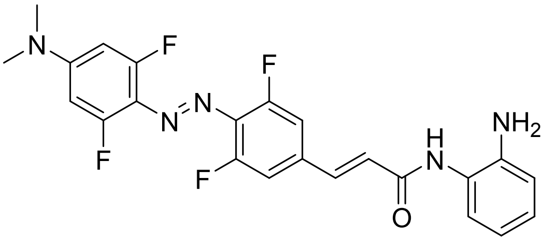

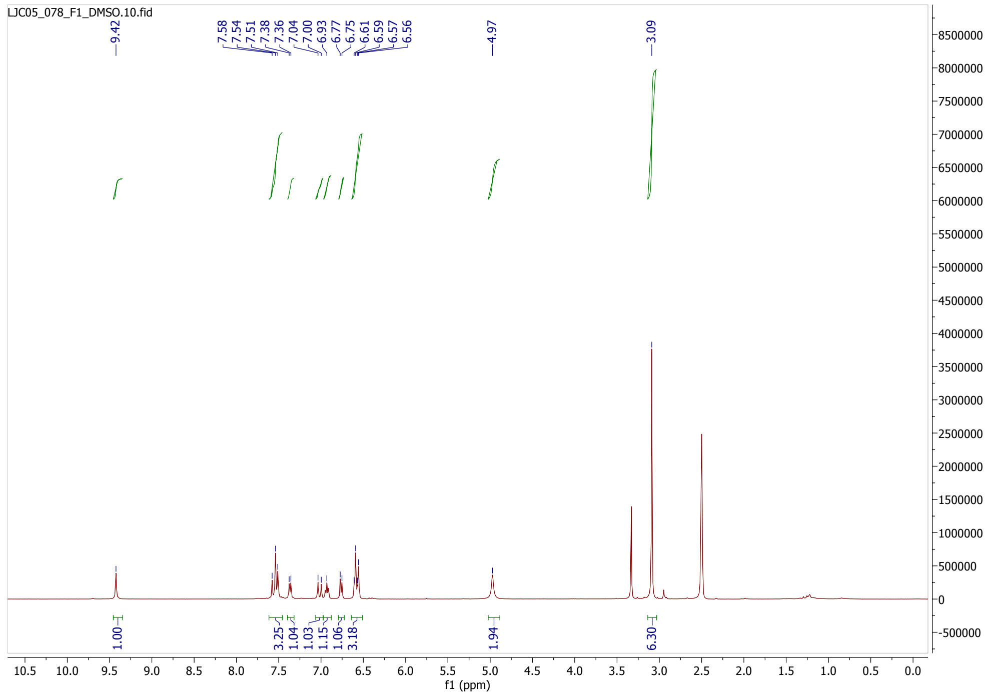

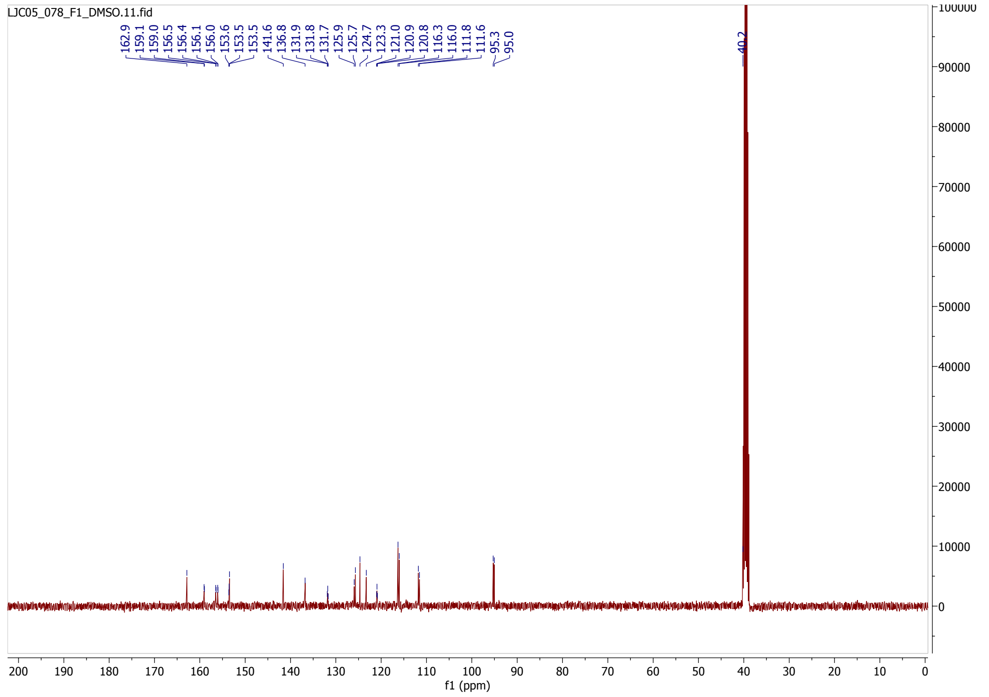

(*E*)-*N*-(2-Aminophenyl)-3-(3,5-dichloro-4-((*E*)-(2,6-dichloro-4-(dimethylamino)phenyl)diazenyl)phenyl)acrylamide (33b)

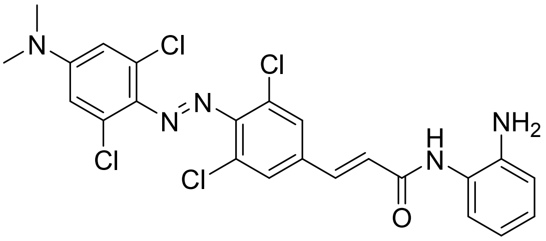

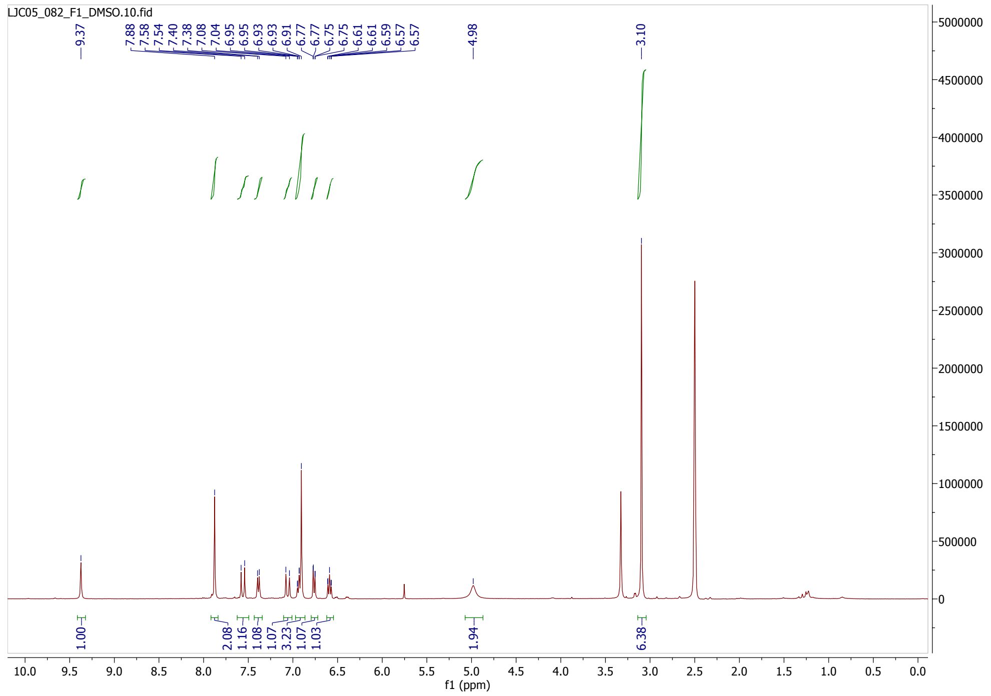

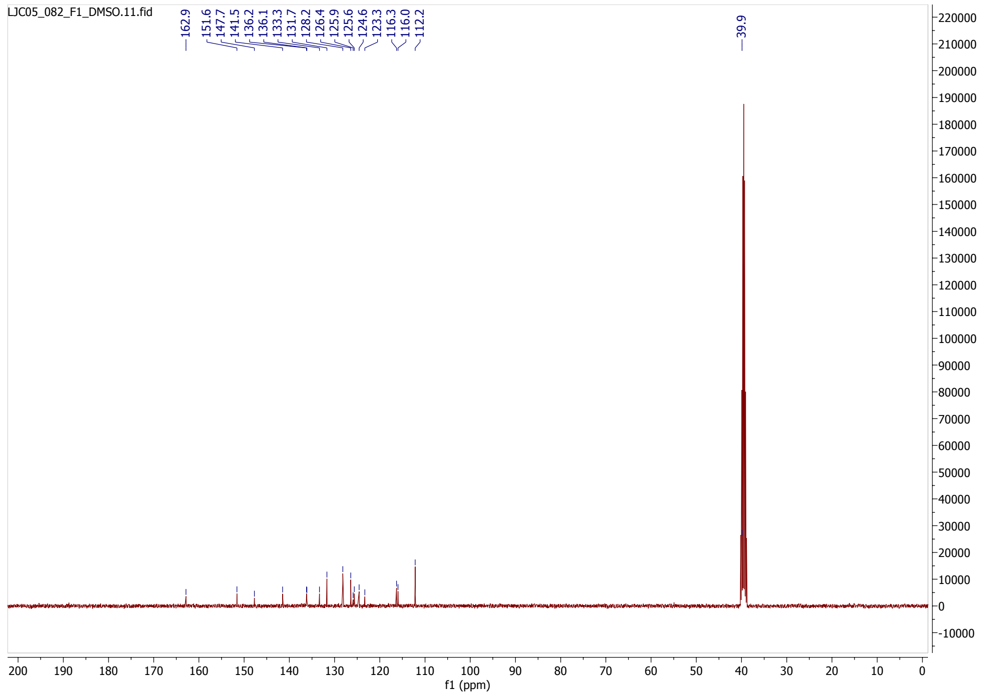

(*E*)-*N*-(2-Aminophenyl)-4-((2,6-difluoro-4-methoxyphenyl)diazenyl)-3,5-difluorobenzamide (38a)

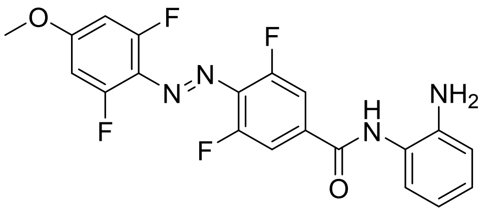

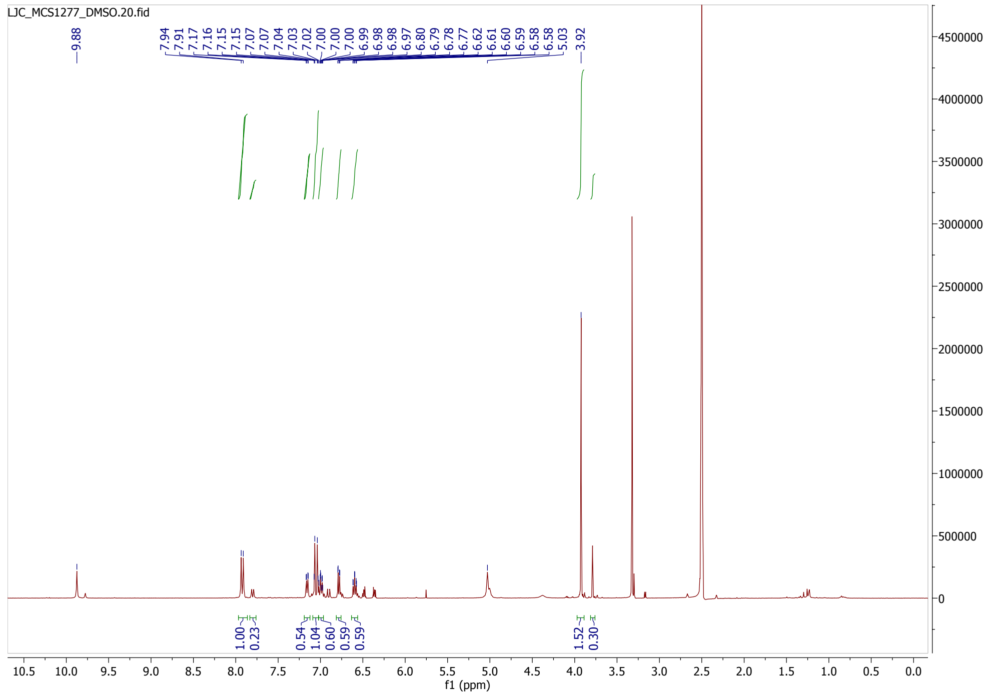

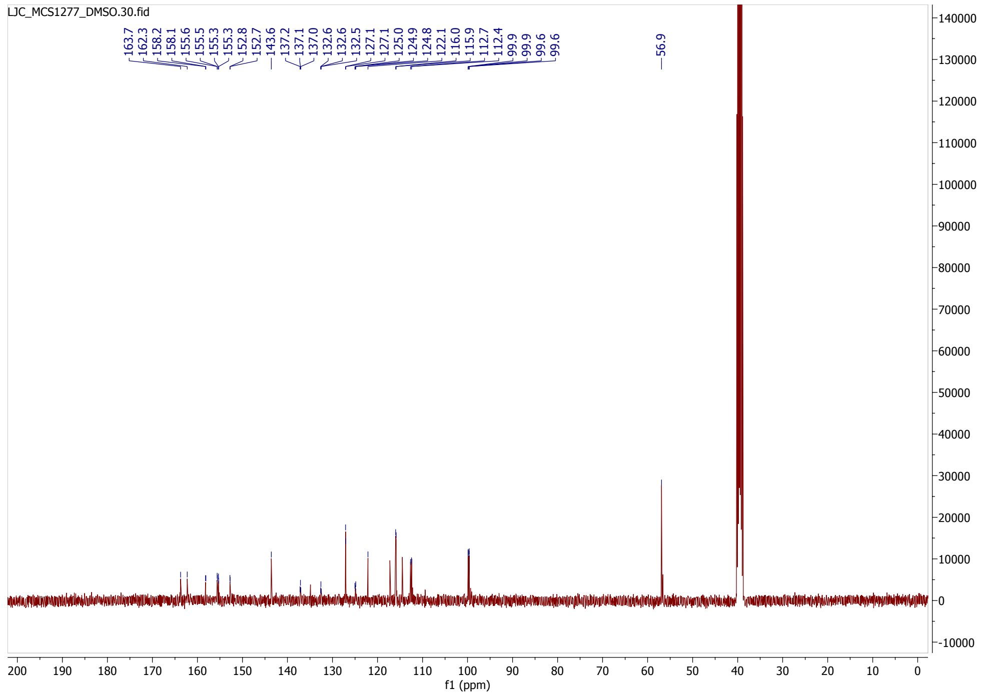

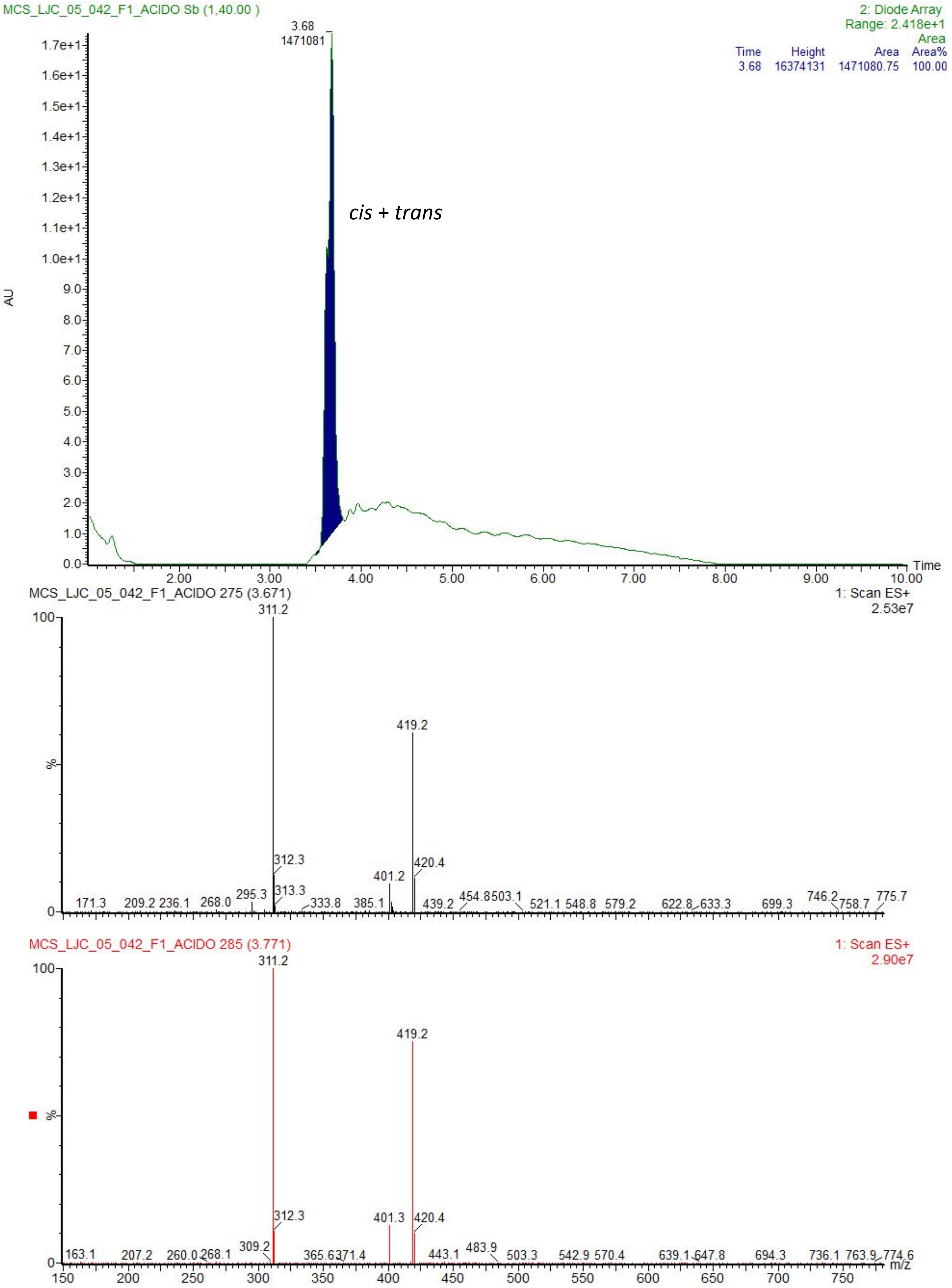
MCS_LJC_05_042_F1_ACIDO Sb (1,40.00)
2: Diode Array
Range: 2.418e+1
Time Height Area Area%
3.68 16374131 1471080.75 100.00
3.68
1471081
cis + trans
AU
Time
MCS_LJC_05_042_F1_ACIDO 275 (3.671)
1: Scan ES+
2.53e7
311.2
419.2
312.3
420.4
401.2
MCS_LJC_05_042_F1_ACIDO 285 (3.771)
1: Scan ES+
2.90e7
311.2
419.2
312.3
401.3
420.4
m/z

(*E*)-*N*-(2-Aminophenyl)-3,5-dichloro-4-((2,6-dichloro-4-methoxyphenyl)diazenyl)benzamide (38b)

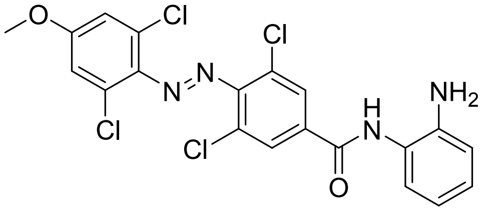

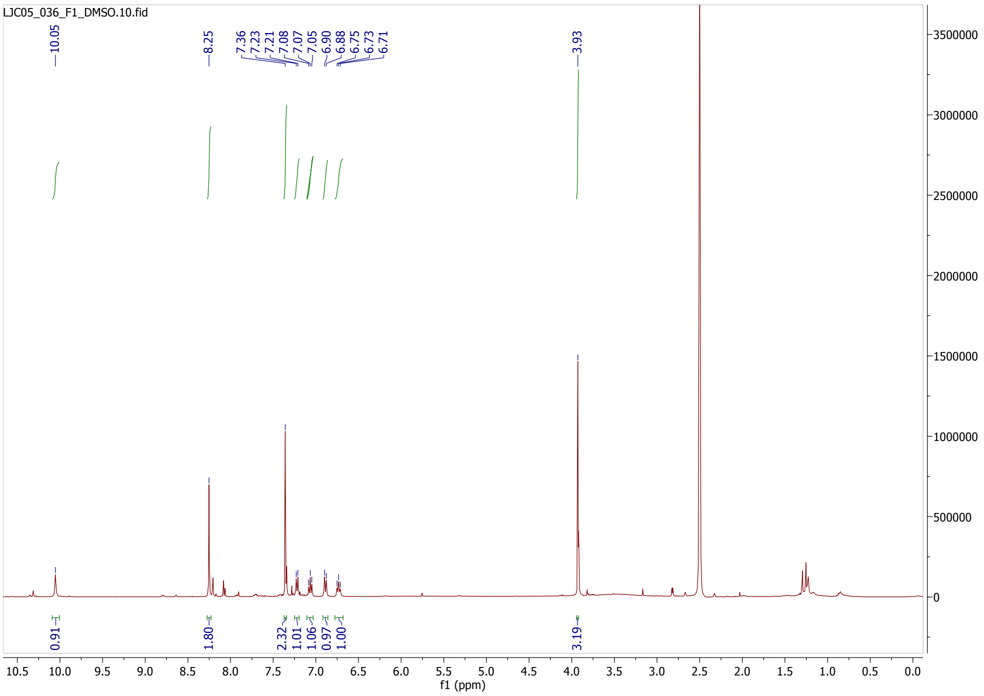

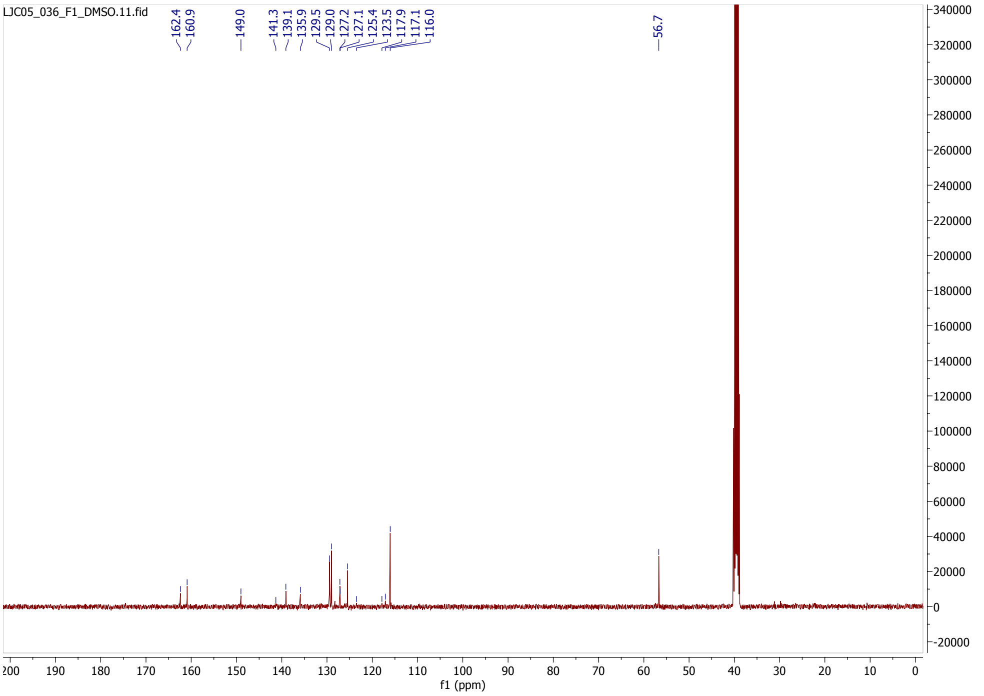

(*E*)-3-(4-((*E*)-(2,6-Difluoro-4-methoxyphenyl)diazenyl)-3,5-difluorophenyl)-*N*-hydroxyacrylamide (39)

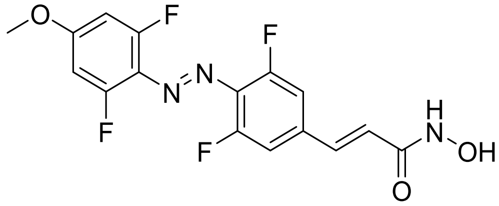

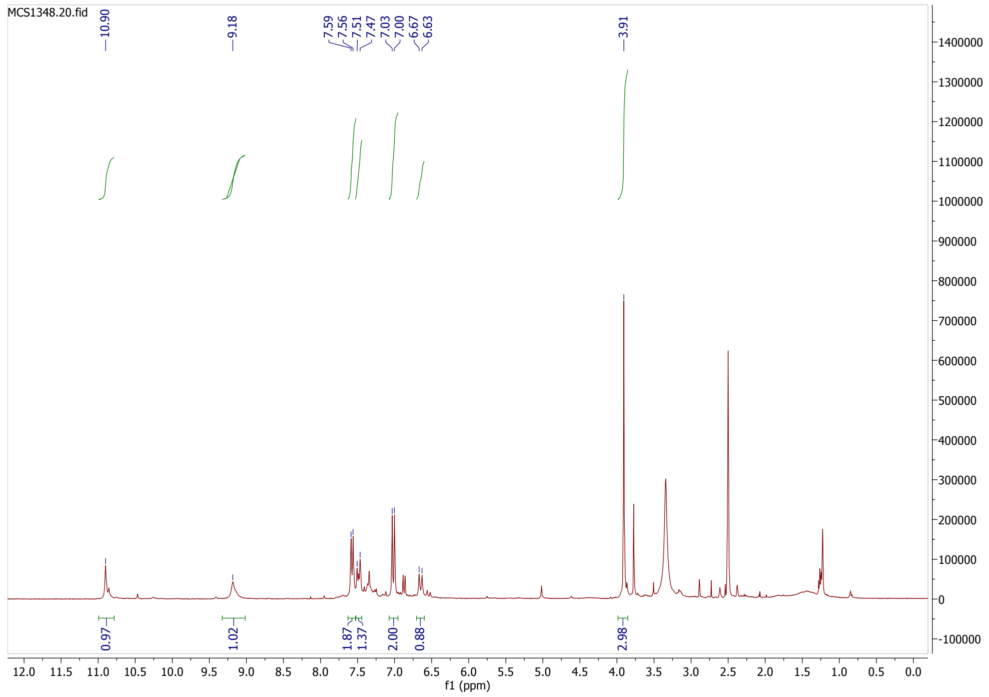

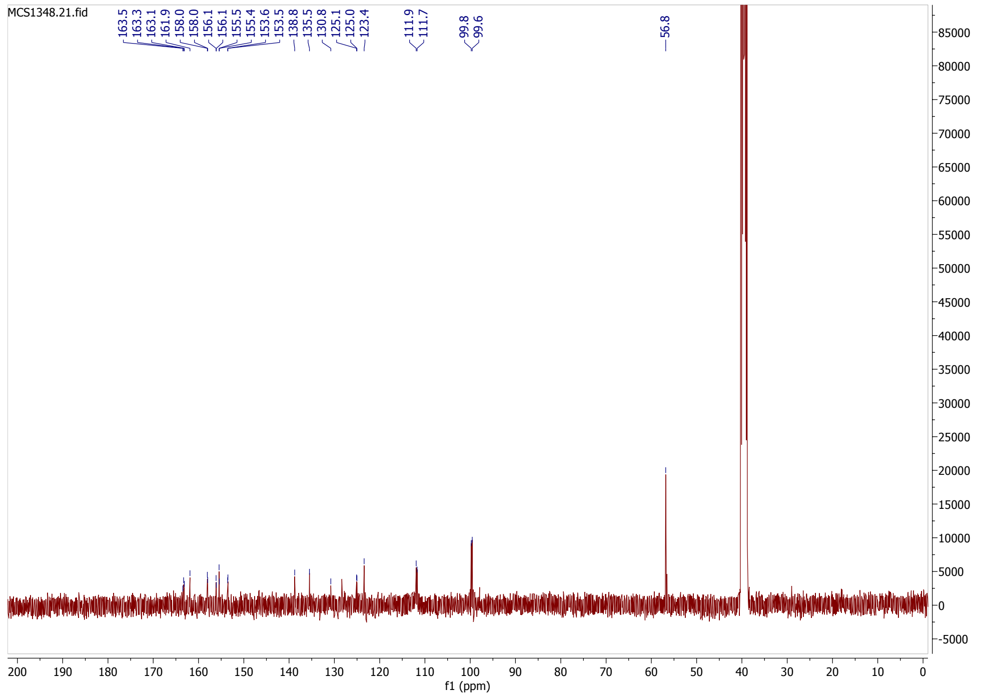

References

1. Methot, J. L. *et al.* Delayed and prolonged histone hyperacetylation with a selective HDAC1/HDAC2 inhibitor. *ACS Med. Chem. Lett.* **5**, 340–345 (2014).
